# Covariate Adjusted Functional Mixed Membership Models

**DOI:** 10.1080/29979676.2025.2566646

**Published:** 2025-10-30

**Authors:** Nicholas Marco, Damla Şentürk, Shafali Jeste, Charlotte DiStefano, Abigail Dickinson, Donatello Telesca

**Affiliations:** a Department of Statistical Science, Duke University, Durham, NC; b Department of Biostatistics, University of California, Los Angeles, CA; c Division of Neurology and Neurological Institute, Children’s Hospital Los Angeles, Los Angeles, CA; d Division of Psychiatry, Children’s Hospital Los Angeles, Los Angeles, CA; e Department of Psychiatry and Biobehavioral Sciences, University of California, Los Angeles, CA

**Keywords:** Clustering, Functional data analysis, Mixed membership models, Neuroimaging

## Abstract

Mixed membership models are a flexible class of models used for unsupervised learning that allow each observation to partially belong to multiple clusters or features. In this article, we extend the framework of functional mixed membership models to allow for covariate-dependent modeling structures. The framework uses a multivariate Karhunen-Loève decomposition, which allows for a scalable and flexible model. Within this framework, we establish a set of sufficient conditions to ensure the identifiability of the mean, covariance, and allocation structure up to a permutation of the labels. This article is primarily motivated by studies on functional brain imaging through electroencephalography (EEG) of children with autism spectrum disorder (ASD). Using the proposed framework, we provide novel insight into the heterogeneity of developmental changes in alpha oscillations and show that individuals with ASD have smaller developmental changes compared to their typically developing counterparts.

## 1. Introduction

Cluster analysis is an unsupervised exploratory task that aims to group similar observations into “clusters” to explain the heterogeneity found in a particular dataset. When an outcome of interest is indexed by covariate information, it is often informative to adjust clustering procedures to account for covariate-dependent patterns of variation. Covariate-dependent clustering has found applications in post hoc analyses of clinical trial data (Müller, Quintana, and Rosner 2011), where conditioning on factors such as dose response, tumor grade, and other clinical factors is often of interest when defining patient response subgroups. In addition to clinical trial settings, covariate-dependent clustering has also become popular in fields such as genetics (Qin and Self 2006), flow cytometry (Hyun et al. 2023), neuroimaging (Binkiewicz, Vogelstein, and Rohe 2017; Guha and Guhaniyogi 2024), and spatial statistics (Page and Quintana 2016).

In statistics and machine learning, covariate-dependent clustering procedures are known by a number of related nomenclatures, including *finite mixture of regressions, mixture of experts*, and *covariate adjusted product partition models*, which tend to refer to specific ways in which covariate information is used in data grouping decisions. The term finite mixture of regressions (Grün et al. 2007; Khalili and Chen 2007; Faria and Soromenho 2010; Devijver 2015; McLachlan, Lee, and Rathnayake 2019; Hyun et al. 2023) refers to the fitting of a mixture model, where the mean structure depends on the covariates of interest through a regression framework. The mixture of experts model (Jordan and Jacobs 1994; Bishop and Svenskn 2002) is similar to a finite mixture of regressions model in that it assumes that the likelihood is a weighted combination of probability distribution functions. However, in the mixture of experts model, the weights are dependent on the covariates of interest, providing an additional layer of flexibility beyond that of traditional finite mixture of regressions models. Although the mixture of experts model was originally designed for supervised learning settings, recent developments have expanded its application to unsupervised settings (Tsai, Li, and Zhu 2020; Kopf et al. 2021). Lastly, covariate adjusted product partition models (Park and Dunson 2010; Müller, Quintana, and Rosner 2011) are a Bayesian nonparametric version of covariate adjusted clustering. Analogous to mixture of experts models, covariate adjusted product partition models allow the cluster partitions to depend on the covariates of interest.

In this article, we extend the class of functional mixed membership models proposed by Marco et al. (2024b) to allow the latent features, representing the different clusters, to depend on covariate information. Mixed membership models (Erosheva, Fienberg, and Lafferty 2004; Heller, Williamson, and Ghahramani 2008; Gruhl and Erosheva 2014; Marco et al. 2024a, 2024b), sometimes referred to as partial membership models, can be thought of as a generalization of finite mixture models, where each observation is allowed to partially belong to multiple clusters or features. Although there have been many advancements in covariate adjusted clustering models for multivariate data, to the best of our knowledge, little work has been done on incorporating covariate information in functional mixed membership models or functional clustering models. Two important exceptions are Yao, Fu, and Lee (2011), who specified a finite mixture of regressions model where the covariates are functional data and the clustered data are scalar, and Gaffney and Smyth (1999) who proposed a functional mixture of regressions model where the function is modeled by a deterministic linear function of the covariates. Here, we consider the case where we have a covariate vector in $R^{p}$ and the outcome we cluster is functional.

Our work is primarily motivated by functional brain imaging studies in children with autism spectrum disorder (ASD), a developmental disorder that is commonly viewed as a spectrum ranging from mild symptoms to severe symptoms. To characterize the differences in brain activity between children with ASD and typically developing children, studies are often conducted using a noninvasive recording method called electroencephalography (EEG). The use of a mixed membership framework is particularly appealing in EEG studies, as EEG spectra consist of a combination of periodic and aperiodic neural activity (Donoghue et al. 2020). Thus, having an unsupervised clustering method that allows partial membership to multiple features is often preferable over traditional clustering methods. Indeed, their usefulness in these types of imaging studies has been demonstrated in Marco et al. (2024b), where they showed that the mixed membership model could separate the aperiodic signal from the periodic signal in the alpha band of frequencies. However, since alpha oscillations are known to change as children develop, the need for an age-dependent mixed membership model is crucial to ensure that shifts in the power spectrum of alpha oscillations do not confound measurements of alpha power (Haegens et al. 2014). In this article, we are interested in characterizing these developmental changes and are interested in learning whether the developmental changes differ between children with ASD and typically developing children. Therefore, in our proposed model, we will assume that each individual’s partial membership does not change with age, allowing us to infer how alpha oscillations change as children age on an individual level.

This article starts with a brief review of functional clustering and covariate-dependent clustering frameworks, such as the mixture of experts model and the finite mixture of regressions model. Using these previous frameworks as a reference, we derive the general form of our covariate adjusted mixed membership model. In Section 2.2 we review the multivariate Karhunen-Loève (KL) theorem, which allows us to have a concise representation of the *K* latent functional features. In Section 2.3, we leverage the KL decomposition to fully specify the covariate adjusted functional mixed membership model. A review of the identifiability issues that occur in mixed membership models, as well as a set of sufficient conditions to ensure identifiability in a covariate adjusted mixed membership model can be found in Section 2.4. Section 3 covers two simulation studies that explore the inferential accuracy of the proposed model and evaluate the performance of various information criteria in choosing the number of features in a covariate adjusted functional mixed membership model. Section 4 illustrates the usefulness of the covariate adjusted functional mixed membership model by analyzing EEG data from children with ASD and TD children. Lastly, we conclude this article with a discussion on some of the challenges of fitting these models, the connection between the proposed model and function-on-scalar regression, as well as possible theoretical challenges when working with covariate adjusted mixed membership models.

## 2. Covariate Adjusted Functional Mixed Membership Model

Functional data analysis (FDA) focuses on the statistical analysis of sample paths from a continuous stochastic process f:𝒯→R, where 𝒯 is a compact subset of $R^{d}$ (Wang, Chiou, and Müller 2016). In FDA, one commonly assumes that the random functions are elements of a Hilbert space, or more specifically that the random functions are square-integrable functions ($f\in L^{2}$ or $\int_{𝒯}|f(t){|}^{2}dt<\infty$). In this article, we assume that the observed sample paths are generated from a Gaussian process (GP), fully characterized by a mean function, μ(t)=E{f(t)}, and a covariance function, C(s,t)=cov{f(s),f(t)}, for t,s∈𝒯. Since mixed membership models can be considered a generalization of finite mixture models, we will show how the finite mixture of regressions and mixture of experts models relate to our proposed mixed membership models. We relate our contribution to covariate adjusted product partition models only superficially, due to the significant differences in both theory and implementation for this model class (Park and Dunson 2010; Müller, Quintana, and Rosner 2011). For the theoretical developments discussed in this section, we will assume that the number of clusters or features, K, is known a-priori. Although the number of clusters or features is often unknown, Section 3.1 shows that the use of information criteria or simple heuristic methods such as the “elbow” method can be informative in choosing the number of features.

### 2.1. Mixture of Regressions, Mixture of Experts and Covariate Adjusted Mixed Membership

Functional clustering generally assumes that each sample path is drawn from one of the K underlying cluster-specific sub-processes (James and Sugar 2003; Chiou and Li 2007; Jacques and Preda 2014). In the GP framework, assuming that $f^{\left(1\right)},\ldots ,f^{\left(K\right)}$ are the K underlying cluster-specific sub-processes with corresponding mean $\left(\mu^{\left(1\right)},\ldots \mu^{\left(K\right)}\right)$ and covariance functions ($C^{\left(1\right)},\ldots ,C^{\left(K\right)}$), the general form of a GP finite mixture model is typically expressed as

(1) $$P\left(f_{i}|\rho^{\left(1:K\right)},\mu^{\left(1:K\right)},C^{\left(1:K\right)}\right)=\overset{K}{\underset{k=1}{\sum }}\rho^{\left(k\right)}𝒢𝒫\left(f_{i}|\mu^{\left(k\right)},C^{\left(k\right)}\right),$$

where $\rho^{\left(k\right)}(\sum_{k=1}^{K}\rho^{\left(k\right)}=1)$ are the mixing proportions and $f_{i}$ are the sample paths for i=1,…,N. Let $x_{i}={\left(X_{i1}\ldots X_{iR}\right)}^{\prime }\in R^{R}:=𝒳$ be the covariates of interest associated with the *i*th observation. Extending the multivariate finite mixture of regressions model (Grün et al. 2007; Khalili and Chen 2007; Faria and Soromenho 2010; Devijver 2015; McLachlan, Lee, and Rathnayake 2019; Hyun et al. 2023) to a functional setting, the covariate adjustments would be encoded through the mean by defining a set of cluster-specific regression functions $\mu^{\left(k\right)}\left(x_{i}\right):𝒳\times 𝒯\to R$. The mixture of GPs in (1) would then be generalized to include covariate information as follows:

(2) $$P\left(f_{i}|x_{i},\rho^{\left(1:K\right)},\mu^{\left(1:K\right)},C^{\left(1:K\right)}\right)\;\;=\overset{K}{\underset{k=1}{\sum }}\rho^{\left(k\right)}𝒢𝒫\left(f_{i}|\mu^{\left(k\right)}\left(x_{i}\right),C^{\left(k\right)}\right),$$

where cluster-specific GPs are now assumed to depend on mean functions $\mu^{\left(k\right)}(x)$, which are allowed to vary over the covariate space. Alternative representations of covariate adjusted mixtures have been proposed to allow the mixing proportions to depend on covariate information through generalized regression functions $\rho^{\left(k\right)}\left(x_{i}\right)$ (Jordan and Jacobs 1994) or implicitly defining covariate adjusted partitions through a cohesion prior (Park and Dunson 2010).

In both the finite mixture of regressions and mixture of experts settings, covariate adjustment does not change the overall interpretation of the finite mixture modeling framework, and the underlying assumption is that a function $f_{i}$ exhibits uncertain membership to *only one of* K well-defined subprocesses $f^{\left(1:K\right)}$. Following Marco et al. (2024b), we contrast this representation of functional data with one where a sample path $f_{i}$ is allowed mixed membership to *one or more* well-defined sub-processes $f^{\left(1:K\right)}$. In the mixed membership framework, the underlying subprocesses $f^{\left(1:K\right)}$ are best interpreted as latent functional features, which may partially manifest in a specific path $f_{i}$.

Our representation of a functional mixed membership model hinges on the introduction of a new set of latent variables $z_{i}={\left(Z_{i1},\ldots ,Z_{iK}\right)}^{⊤}$, where $Z_{ik}\in [0,1]$ and $\sum_{k=1}^{K}Z_{ik}=1$. We will refer to these latent variables as *allocation parameters*. Given the latent allocation parameters $z_{i}$, each sample path is assumed to follow the following sampling structure:

(3) $$f_{i}|z_{1},\ldots ,z_{N}{=}_{d}\overset{K}{\underset{k=1}{\sum }}Z_{ik}f^{\left(k\right)}.$$

Thus, we can see that under the functional mixed membership model, each sample path is assumed to come from a convex combination of the underlying GPs, $f^{\left(k\right)}$. To avoid unwarranted restrictions on the implied covariance structure, the functional mixed membership model does not assume that the underlying GPs are mutually independent. Thus, we will let $C^{\left(k,j\right)}$ represent the cross-covariance function between the *k*th GP and the *j*th GP, for 1≤k≠j≤K. Letting 𝓒 be the collection of covariance and cross-covariance functions, we can specify the sampling model of the functional mixed membership model as

(4) $$f_{i}|z_{i},\mu^{\left(1:K\right)},𝓒∼𝒢𝒫(\overset{K}{\underset{k=1}{\sum }}Z_{ik}\mu^{\left(k\right)},\overset{K}{\underset{k=1}{\sum }}Z_{ik}^{2}C^{\left(k\right)}\;\;+\overset{K}{\underset{k=1}{\sum }}\underset{k^{\prime }\neq k}{\sum }Z_{ik}Z_{ik^{\prime }}C^{\left(k,k^{\prime }\right)}).$$

Leveraging the mixture of regressions framework in (2), the proposed covariate adjusted functional mixed membership model becomes:

(5) $$f_{i}|X,z_{1},\ldots ,z_{N},\mu^{\left(1:K\right)}\left(x_{i}\right),𝓒\;\;∼𝒢𝒫\left(\overset{K}{\underset{k=1}{\sum }}Z_{ik}\mu^{\left(k\right)}\left(x_{i}\right),\overset{K}{\underset{k=1}{\sum }}Z_{ik}^{2}C^{\left(k\right)}+\overset{K}{\underset{k=1}{\sum }}\underset{k^{\prime }\neq k}{\sum }Z_{ik}Z_{ik^{\prime }}C^{\left(k,k^{\prime }\right)}\right).$$

In Section 2.2, we review the multivariate Karhunen-Loève (KL) construction (Ramsay and Silverman 2005b; Happ and Greven 2018), which allows us to have a joint approximation of the covariance structure of the *K* underlying GPs. Using the joint approximation, we are able to concisely represent the *K* GPs, facilitating inference on higher-dimensional functions such as surfaces. Using the KL decomposition, we are able to fully specify our proposed covariate adjusted functional mixed membership model (5) in Section 2.3.

### 2.2. Multivariate Karhunen-Loève Characterization

The proposed modeling framework in (5) depends on a set of mean and covariance functions, which are naturally represented by infinite-dimensional parameters. Thus, in order to work in finite dimensions, we assume that the underlying GPs are smooth and lie in the P-dimensional subspace, $𝓢\subset L^{2}(𝒯)$, spanned by a set of P linearly independent square-integrable basis functions, $\left\{b_{1},\ldots ,b_{P}\right\}\left(b_{p}:𝒯\to R\right)$. Although the choice of basis functions is user-defined, the basis functions must be uniformly continuous in order to satisfy Lemma 2.2 of Marco et al. (2024b). In this article, we will use B-splines for all case studies and simulation studies.

The assumption that $f^{\left(k\right)}\in 𝓢$ allows us to turn an infinite-dimensional problem into a finite-dimensional problem, making traditional inference tractable. Although tractable, accounting for covariance and cross-covariance functions separately leads to a model that needs $𝒪\left(K^{2}P^{2}\right)$ parameters to represent the overall covariance structure. While the number of clusters, K, supported in applications is typically small, the number of basis functions, P, tends to be proportional to the observational grid resolution and may therefore be quite large. This is particularly true when one considers higher-dimensional functional data, such as surfaces, which necessitate a substantial number of basis functions, making it computationally expensive to fit models. Thus, we will use the multivariate KL decomposition (Ramsay and Silverman 2005b; Happ and Greven 2018) to reduce the number of parameters needed to estimate the covariance surface to 𝒪(KPM), where M is the number of eigenfunctions used to approximate the covariance structure.

To achieve a concise joint representation of the *K* latent GPs, we define a multivariate GP, which we denote by **f**(**t**), such that

$$f(t):=\left\{f^{\left(1\right)}\left(t^{\left(1\right)}\right),f^{\left(2\right)}\left(t^{\left(2\right)}\right),\ldots ,f^{\left(K\right)}\left(t^{\left(K\right)}\right)\right\},$$

where $t=\left(t^{\left(1\right)},t^{\left(2\right)},\ldots ,t^{\left(K\right)}\right)$ and $t^{\left(1\right)},t^{\left(2\right)},\ldots ,t^{\left(K\right)}\in 𝒯$. Since $𝓢\subset L^{2}$ is a Hilbert space, with the inner product defined as the $L^{2}$ inner product, we can see that $f\in 𝓗:=𝓢\times 𝓢\times \cdots \times 𝓢:={𝓢}^{K}$, where 𝓗 is defined as the direct sum of the Hilbert spaces 𝓢, making 𝓗 also a Hilbert space. Since 𝓗 is a Hilbert space and the covariance operator of f, denoted 𝓚, is a positive, bounded, self-adjoint, and compact operator, we know that there exists a complete set of eigenfunctions $\Psi_{1},\ldots ,\Psi_{KP}\in 𝓗$ and the corresponding eigenvalues $\lambda_{1}\geq \cdots \geq \lambda_{KP}\geq 0$ such that $𝓚\Psi_{p}=\lambda_{p}\Psi_{p}$, for p=1,…,KP. Using the eigenpairs of the covariance operator, we can rewrite $f^{\left(k\right)}$ as

$$f^{\left(k\right)}(t)=\mu^{\left(k\right)}(x,t)+\overset{KP}{\underset{m=1}{\sum }}\chi_{m}\left(\sqrt{\lambda_{m}}{\text{Ψ}}_{m}^{\left(k\right)}(t)\right),$$

where $\chi_{m}∼𝒩(0,1)$ and $\Psi_{m}^{\left(k\right)}(t)$ is the *k*th element of $\Psi_{m}(t)$. Since $\Psi_{m}^{\left(k\right)}(t)\in 𝓢$, we know that there exists $\phi_{km}\in R^{P}$ such that $\sqrt{\lambda_{m}}\Psi_{m}^{\left(k\right)}(t)=\phi_{km}^{⊤}B(t)$, where $B^{⊤}(t)\;:=\left[b_{1}(t),b_{2}(t),\ldots ,b_{P}(t)\right]$. Similarly, since $\mu^{\left(k\right)}(x,t)\in 𝓢$, we can introduce a mapping $g\;:\;R^{R}\to R^{P}$, such that $\mu^{\left(k\right)}(x,t)=g_{k}(x{)}^{⊤}B(t)$. Therefore, we arrive at the general form of our decomposition:

(6) $$f^{\left(k\right)}(t)=g_{k}{\left(x\right)}^{⊤}B(t)+\overset{KP}{\underset{m=1}{\sum }}\chi_{m}\phi_{km}^{⊤}B(t).$$

Using this decomposition, our covariance and mean structures can be recovered such that $C^{\left(k,k^{\prime }\right)}(s,t)=B^{⊤}(s)\left(\sum_{m=1}^{KP}\phi_{km}\phi_{k^{\prime }m}^{⊤}\right)B(t)$ and $\mu^{\left(k\right)}(x,t)=g_{k}{\left(x\right)}^{⊤}B(t)$, for $1\leq k,k^{\prime }\leq K$ and s,t∈𝒯. To reduce the dimensionality of the problem, we will only use the first M eigenpairs to approximate the K stochastic processes, where 1≤M≤KP. Although traditional functional principal component analysis (FPCA) will choose the number of eigenfunctions based on the proportion of variance explained, the same strategy cannot be employed in functional mixed membership models because the allocation parameters of the model are typically not known. Therefore, we suggest selecting a large value of M, and then proceeding with stable estimation through prior regularization.

### 2.3. Model and Prior Specification

In this section, we fully specify the covariate adjusted functional mixed membership model using a truncated version of the KL decomposition, specified in (6). We start by first specifying how the covariates of interest influence the mean function. Under the standard function-on-scalar regression framework (Faraway 1997; Brumback and Rice 1998), we have $\mu^{\left(k\right)}\left(x_{i},t\right)=\beta_{k0}+\sum_{r=1}^{R}X_{ir}\beta_{kr}(t)$ for k=1,…,K. Since we assumed that $\mu^{\left(k\right)}\left(x_{i},t\right)\in 𝓢$ for k=1,…,K, we know that $\beta_{k0},\ldots ,\beta_{kR}\in 𝓢$. Therefore, there exist $v_{k}\in R^{P}$ and $\eta_{k}\in R^{P\times R}$ such that

(7) $$\mu^{\left(k\right)}\left(x_{i},t\right)=v_{k}^{⊤}B(t)+{\left(\eta_{k}x_{i}^{⊤}\right)}^{⊤}B(t).$$

Under a standardized set of covariates, $v_{k}$ specifies the population-level mean of the *k*th feature and $\eta_{k}$ encodes the covariate dependence of the kth feature. Thus, assuming the mean structure specified in (7) and using a truncated version of the KL decomposition specified in (6), we can specify the sampling model of our covariate adjusted functional mixed membership model.

Let ${\left\{Y_{i}(.)\right\}}_{i=1}^{N}$ be the observed sample paths that we want to model to the K features, $f^{\left(k\right)}$, conditionally on the covariates of interest, $x_{i}$. Since the observed sample paths are observed at only a finite number of points, we will let $t_{i}={\left[t_{i1},\ldots ,t_{in_{i}}\right]}^{⊤}$ denote the time points at which the *i*th function was observed. Without loss of generality, we will define the sampling distribution over the finite-dimensional marginals of $Y_{i}\left(t_{i}\right)$, representing the ith observed sample path evaluated at time points $t_{i}$. Using the general form of our proposed model defined in (5), we have

(8) $$Y_{i}\left(t_{i}\right)|\Theta ,X∼𝒩\{\overset{K}{\underset{k=1}{\sum }}Z_{ik}(S^{⊤}\left(t_{i}\right)\left(v_{k}+\eta_{k}x_{i}^{⊤}\right)\;\;+\overset{M}{\underset{m=1}{\sum }}\chi_{im}S^{⊤}\left(t_{i}\right)\phi_{km}),\sigma^{2}I_{n_{i}}\},$$

where $S\left(t_{i}\right)=\left[B\left(t_{1}\right)\cdots B\left(t_{n_{i}}\right)\right]\in R^{P\times n_{i}}$ and Θ is the collection of the model parameters. As defined in Section 2.1, $Z_{ik}$ are variables that lie on the unit simplex, such that $Z_{ik}\in (0,1)$ and $\sum_{k=1}^{K}Z_{ik}=1$. From this characterization, we can see that each observation is modeled as a convex combination of realizations from the K features with additional Gaussian noise, represented by $\sigma^{2}$. If we integrate out the $\chi_{\mathrm{im}}$ variables, for i=1,…,N and m=1,…,M, we arrive at the following likelihood:

(9) $$Y_{i}\left(t_{i}\right)|\Theta_{-\chi },X∼𝒩\{\overset{K}{\underset{k=1}{\sum }}Z_{ik}S^{⊤}\left(t_{i}\right)\left(v_{k}+\eta_{k}x_{i}^{⊤}\right),\;\;\times V\left(t_{i},z_{i}\right)+\sigma^{2}I_{n_{i}}\},$$

where $\Theta_{-\chi }$ is the collection of the model parameters excluding the $\chi_{\text{im}}$ parameters (i=1,…,N and m=1,…,M and $V\left(t_{i},z_{i}\right)=\sum_{k=1}^{K}\sum_{k^{\prime }=1}^{K}Z_{ik}Z_{ik^{\prime }}\left\{S^{⊤}\left(t_{i}\right)\sum_{m=1}^{M}\left(\phi_{km}\phi_{k^{\prime }m}^{⊤}\right)S\left(t_{i}\right)\right\}$. Equation (9) illustrates that the proposed covariate adjusted functional mixed membership model can be expressed as an additive model; the mean structure is a convex combination of the feature-specific means, while the covariance can be written as a weighted sum of covariance functions and cross-covariance functions.

To have an adequately expressive and scalable model, we approximate the covariance surface of the K features using M scaled *pseudo-eigenfunctions*. In this framework, orthogonality will not be imposed on the $\phi_{km}^{⊤}B(t)$ parameters, making them pseudo-eigenfunctions instead of the eigenfunctions described in Section 2.2. From a modeling perspective, this allows us to sample on an unconstrained space, facilitating better Markov chain mixing and easier sampling schemes. Although direct inference on the eigenfunctions is no longer available, a formal analysis can still be conducted by reconstructing the posterior samples of the covariance surface and calculating eigenfunctions from the posterior samples. To avoid overfitting of the covariance functions, we follow Marco et al. (2024b) by using the multiplicative gamma process shrinkage prior proposed by Bhattacharya and Dunson (2011) to achieve adaptive regularized estimation of the covariance structure. Therefore, letting $\phi_{kpm}$ be the *p*th element of $\phi_{km}$, we have the following:

$$\phi_{kpm}|\gamma_{kpm},{\tilde{\tau }}_{mk}∼𝒩\left(0,\gamma_{kpm}^{-1}{\tilde{\tau }}_{mk}^{-1}\right),\;\;\gamma_{kpm}∼\text{Γ}\left(v_{\gamma }/2,v_{\gamma }/2\right),\;\;{\tilde{\tau }}_{mk}=\overset{m}{\underset{n=1}{\prod }}\delta_{nk},$$

$$\delta_{1k}|a_{1k}∼\text{Γ}\left(a_{1k},1\right),\;\delta_{jk}|a_{2k}∼\text{Γ}\left(a_{2k},1\right),\;\;a_{1k}∼\text{Γ}\left(\alpha_{1},\beta_{1}\right),\;a_{2k}∼\text{Γ}\left(\alpha_{2},\beta_{2}\right),$$

where 1≤k≤K,1≤p≤P,1≤m≤M, and 2≤j≤M. By letting $\alpha_{2}>\beta_{2}$, we can show that $E\left({\tilde{\tau }}_{mk}\right)>E\left({\tilde{\tau }}_{m^{\prime }k}\right)$ for $1\leq m<m^{\prime }\leq M$, leading to the prior on $\phi_{kpm}$ having stochastically decreasing variance as m increases. This will have a regularizing effect on the posterior draws of $\phi_{kpm}$, making $\phi_{kpm}$ more likely to be close to zero as m increases.

In functional data analysis, we often desire smooth mean functions to protect against overfit models. Therefore, we use a first-order random walk penalty proposed by Lang and Brezger (2004) on the $v_{k}$ parameters and each column of the $\eta_{k}$ parameters to promote adaptive smoothing of the mean functions of each feature in our model. Therefore, we have that $P\left(v_{k}|\tau_{v_{k}}\right)\propto exp\left(-\frac{\tau_{v_{k}}}{2}\sum_{p=1}^{P-1}{\left(v_{pk}-v_{(p+1)k}\right)}^{2}\right)$, for k=1,…,K, where $\tau_{v_{k}}∼\Gamma \left(\alpha_{v},\beta_{v}\right)$ and $\nu_{pk}$ is the *p*th element of $v_{k}$. Similarly, we have that $P({\left\{\eta_{prk}\right\}}_{p=1}^{P}|\tau_{\eta_{rk}})\propto exp\left(-\frac{\tau_{\eta_{rk}}}{2}\sum_{p=1}^{P-1}{\left(\eta_{prk}-\eta_{(p+1)rk}\right)}^{2}\right)$, for k=1,…,K and r=1,…,R, where $\tau_{\eta_{rk}}∼\Gamma (\alpha_{\eta },\beta_{\eta })$ and $\eta_{prk}$ is the pth row and rth column of $\eta_{k}$. Following previous mixed membership models (Heller, Williamson, and Ghahramani 2008; Marco et al. 2024b,a), we use a hierarchical Dirichlet prior, such that $z_{i}|\pi ,\alpha_{3}{∼}_{\mathrm{iid}}Dir\left(\alpha_{3}\pi \right),\pi ∼\mathrm{Dir}\left(c_{\pi }\right)$, and $\alpha_{3}∼Exp(b)$. Lastly, we assume that $\sigma^{2}∼IG\left(\alpha_{0},\beta_{0}\right)$. The posterior distributions of the parameters in our model, as well as a sampling scheme with tempered transitions, can be found in Section 2 of the supplementary materials. A covariate adjusted model where the covariance is also dependent on the covariates of interest can be found in Section 4 of the supplementary materials.

### 2.4. Model Identifiability

Mixed membership models face multiple identifiability problems due to their increased flexibility over traditional clustering models (Chen et al. 2022; Marco et al. 2024a, 2024b). Like traditional clustering models, mixed membership models also face the common *label switching* problem, where an equivalent model can be formulated by permuting the labels or allocation parameters. Although this is one source of non-identifiability, relabelling algorithms can be formulated from the work of Stephens (2000). More complex identifiability problems arise since the allocation parameters are now continuous variables on the unit simplex, rather than binary variables like in clustering. Specifically, an equivalent model can be constructed by rotating and/or changing the volume of the convex polytope constructed by the allocation parameters through linear transformations of the allocation parameters (Chen et al. 2022). Therefore, geometric assumptions must be made on the allocation parameters to ensure that the mixed membership models are identifiable.

One way to ensure that we have an identifiable model up to a permutation of the labels is to assume that the *separability condition* holds (Pettit 1990; Azar et al. 2001; Donoho and Stodden 2003; Arora, Ge, and Moitra 2012; Chen et al. 2022). The separability condition assumes that at least one observation belongs completely to each of the *K* features. These observations that belong only to one feature act as “anchors”, ensuring that the allocation parameters are identifiable up to a permutation of the labels. Although the separability condition is conceptually simple and relatively easy to implement, it makes strong geometric assumptions on the data-generating process for mixed membership models with three or more features. Weaker geometric assumptions, known as the *sufficiently scattered* condition (Chen et al. 2022), which ensure an identifiable model in mixed membership models with 3 or more features are discussed in Chen et al. (2022). Although the geometric assumptions are relatively weak, implementing these constraints is nontrivial (Marco et al. 2024b). When extending multivariate mixed membership models to functional data and introducing a covariate-dependent mean structure, ensuring an identifiable mean and covariance structure requires further assumptions.

*Lemma 2.1.* Consider a K-feature covariate adjusted functional mixed membership model as specified in (9). The parameters $v_{k},\eta_{k},Z_{ik},\sum_{m=1}^{M}\left(\phi_{km}\phi_{k^{\prime }m}^{⊤}\right)$, and $\sigma^{2}$ are identifiable up to a permutation of the labels (i.e., label switching), for $k,k^{\prime }=1,\ldots ,K,i=1,\ldots ,N$, and m=1,…,M, given the following assumptions:

1. X is full column rank with 1 not in the column space of X.
2. The separability condition or the sufficiently scattered condition holds on the allocation matrix. Moreover, there exist at least $\frac{K^{2}+K}{2}$ observations ($N\geq \frac{K^{2}+K}{2}$), with allocation parameters such that the matrix $C\in R^{N\times \frac{K^{2}+K}{2}}$ is full column rank, where the *i*th row of C is specified as $c_{i}=\left[Z_{i1}^{2},\ldots ,Z_{iK}^{2},2Z_{i1}Z_{i2},\ldots ,2Z_{i1}Z_{iK},2Z_{i2}Z_{i3},\ldots ,2Z_{i(K-1)}Z_{iK}\right]$.
3. The sample paths $Y_{i}\left(t_{i}\right)$ are regularly sampled so that $n_{i}>P$. Moreover, we will assume that the basis functions are B-splines with equidistant knots.

Lemma 2.1 states a set of sufficient conditions that lead to an identifiable mean and covariance structure up to a permutation of the labels. The proof of Lemma 2.1 can be found in Section 1 of the supplementary materials. The first assumption is similar to the assumption needed in the linear regression setting to ensure identifiability; however, the intercept term is not included in the design matrix, as the $v_{k}$ parameters already account for the intercept. In the second assumption, we assume geometric assumptions on the allocation parameters, provide a lower bound for the number of functional observations, and assume that the matrix **C** is full column rank. The matrix **C** is constructed using the allocation parameters and, although the condition on **C** may seem restrictive, it often holds in practice. We note that this condition may not hold if most of your allocation parameters lie on a lower-dimensional subspace of the unit (*K* − 1)-simplex. Although the individual $\phi_{\mathrm{km}}$ parameters are not identifiable in our model, an eigen analysis can still be performed by constructing posterior draws of the covariance structure and calculating the eigenvalues and eigenfunctions of the posterior draws. Section 3 provides empirical evidence that the mean and covariance structure converge to the truth as more observations are accrued.

## 3. Simulation Studies

In this section, we will discuss the results of two simulation studies. The first simulation study explores the inferential accuracy of the mean, covariance, and allocation structures of the proposed covariate adjusted functional mixed membership model under various sample sizes. Along with correctly specified models, this simulation study also studies the convergence properties of over-specified and under-specified models. The second simulation study explores the use of information criteria in choosing the number of features in a covariate adjusted functional mixed membership model.

### 3.1. Simulation Study 1: Structure Recovery

In the first simulation study, we explore the inferential accuracy of the proposed covariate adjusted functional mixed membership models to see how well the proposed framework can recover the true mean, covariance, and allocation structures. Here, we generate 50 different datasets from our proposed model for each of the nine scenarios considered. The nine scenarios consist of data generated from the proposed model with two covariates, one covariate, and no covariates (R=0,1,2), each with three different sample sizes. Each sample path was generated with 25 observed time points ($n_{i}=25$) that were observed on a regular grid. In addition to evaluating the inferential accuracy under the correctly specified model, we also were interested in studying how models with incorrectly specified design matrices performed. Specifically, for the data generated from only one covariate, we fit a functional mixed membership model with no covariates, and for the data generated using no covariates, we fit a model that used one randomly generated covariate. By studying this, we could get a sense of the statistical efficiency lost due to adding additional covariates, as well as insight into how well we can recover the marginal structures when key covariates are not included in the design matrix. To evaluate how well we can recover the mean, covariance, and cross-covariance functions, we calculate the relative mean integrated square error (R-MISE), which is defined as $\mathrm{R-MISE}=\frac{\int \{f(t)-\overset{ˆ}{f}(t){\}}^{2}dt}{\int f(t{)}^{2}dt}\times 100\%$ or $\mathrm{R-MISE}=\frac{\int_{t}\int_{x}\{f(t,x)-\overset{ˆ}{f}(t,x){\}}^{2}dxdt}{\int_{t}\int_{x}f(t,x{)}^{2}dxdt}\times 100\%$ in the case of a covariate adjusted model. In this simulation study, $\overset{ˆ}{f}(t)$ will be the posterior median obtained from the posterior samples. To measure how well we recover the allocation structure, $Z_{ik}$, we calculated the root-mean-square error (RMSE).

**Figure 1.**
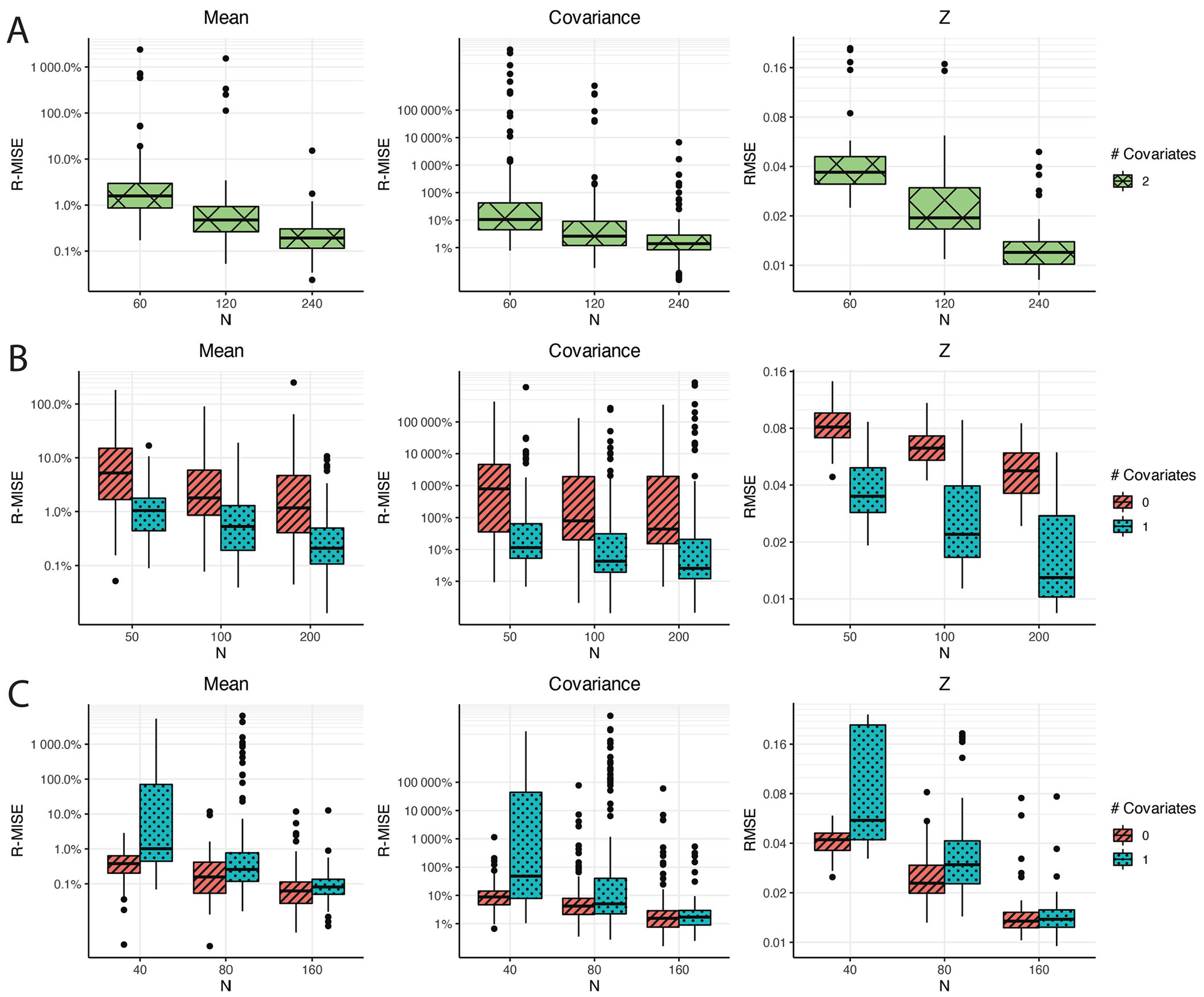
Recovery of the mean, covariance, and allocation structure under the following three data-generating scenarios: (1) data generated from a two-feature covariate adjusted functional mixed membership model with two covariates (Row A), (2) data generated from a two-feature covariate adjusted functional mixed membership model with one covariate (Row B), and (3) data generated from a two-feature functional mixed membership model with no covariates (Row C).

Figure 1 contains a visualization of the performance metrics from each of the scenarios considered in this simulation study. We can see that when the model is specified correctly, it does a good job in recovering the mean structure, even with relatively few observations. On the other hand, a relatively large number of observations are needed to recover the covariance structure even when we have a correctly specified model. This simulation study also shows that we pay a penalty in terms of statistical efficiency when we over-specify a model; however, the over-specified model still shows signs of convergence to the true mean, covariance, and allocation structures. When we do not include key covariates that were used to generate the model, we can still see signs that our estimation of the marginal mean structure, the marginal covariance structure, and the allocation structure improves as we get more data. However, we can see that the R-MISE decreases much slower compared to a correctly specified model.Additional details on how the simulation was conducted, as well as more detailed visualizations of the simulation results, can be found in Section 3.1 of the supplementary materials.

### 3.2. Simulation Study 2: Information Criteria

When considering the use of a mixed membership model to gain insight into the heterogeneity found in a dataset, a practitioner must specify the number of features to use in the mixed membership model. Correctly specifying the number of features is crucial, as an over-specification will lead to challenges in interpretability of the results, and an under-specification will lead to a model that is not flexible enough to model the heterogeneity in the data. Although information criteria such as BIC and heuristics such as the elbow-method have been shown to be informative in choosing the number of parameters in mixed membership models (Marco et al. 2024a, 2024b), it is unclear whether these results extend to covariate adjusted models. In this case, we consider the performance of AIC (Akaike 1974), BIC (Schwarz 1978), DIC (Spiegelhalter et al. 2002; Celeux et al. 2006), and the elbow-method in choosing the number of features in a covariate adjusted functional mixed membership model with one covariate. The information criteria used in this section are specified in Section 3.2 of the supplementary materials. As specified, the optimal model should have the smallest AIC, the largest BIC, and the smallest DIC.

**Figure 2.**
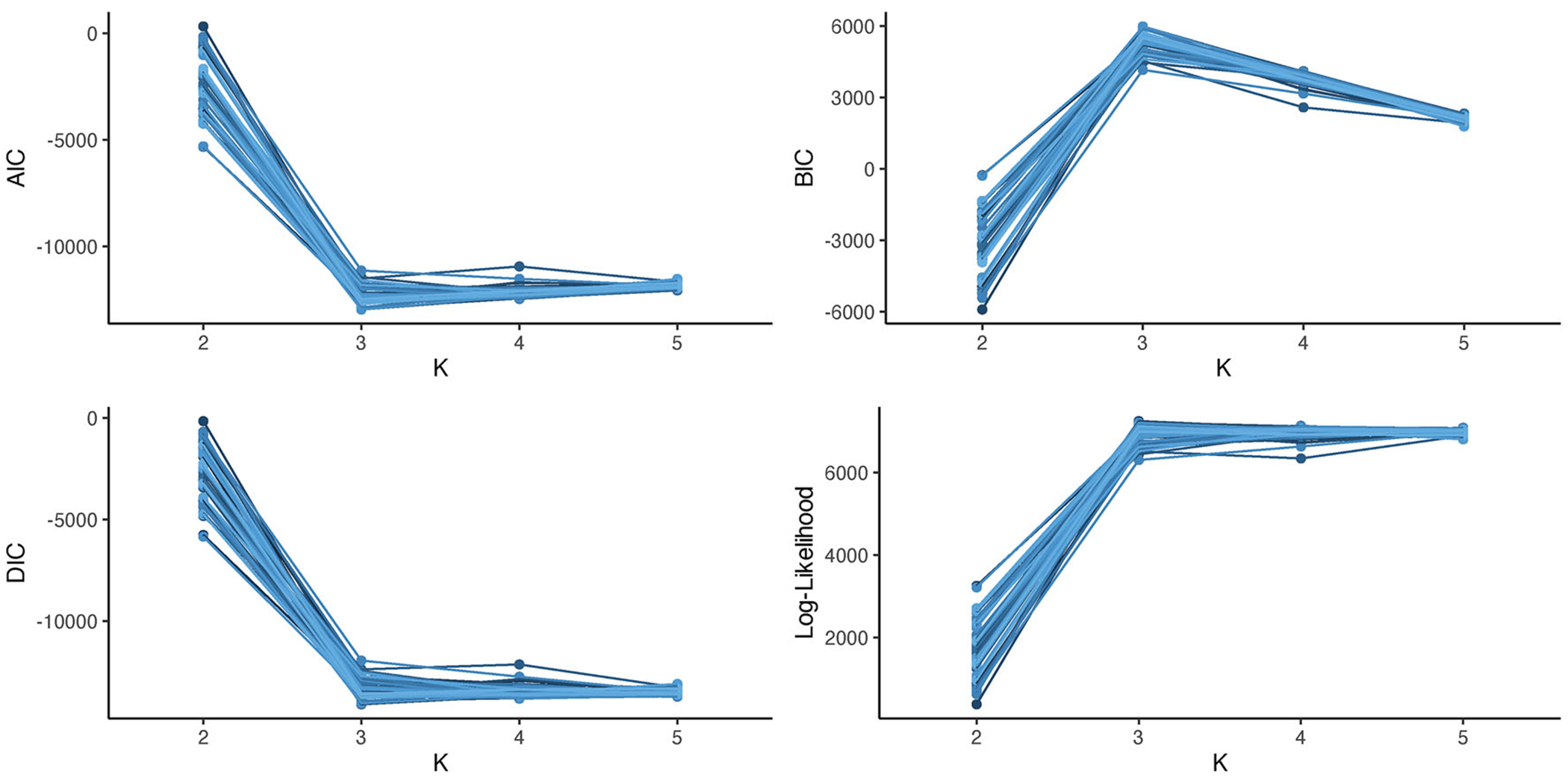
Estimated values of AIC, BIC, DIC, and the log-likelihood for the 50 simulated datasets. The true datasets were generated from a covariate adjusted functional mixed membership with three features.

To evaluate the performance of the information criteria and the elbow method, 50 datasets were generated from a covariate adjusted functional mixed membership model with three features (K = 3) and one covariate (R = 1). Each dataset was analyzed using four covariate adjusted functional mixed membership models; each with a single covariate and a varying number of features (*K* = 2, 3, 4, 5). Figure 2 shows the performance of the three information criteria. Similarly to Marco et al. (2024b) and Marco et al. (2024a), we can see that BIC had the best performance, picking the three-feature model all 50 times. From the simulation study, we found that AIC and DIC tended to choose more complex models, with AIC and DIC choosing the correctly specified model 68% and 26% of the time, respectively. Looking at the log-likelihood plot, we can see that there is a distinct elbow at *K* = 3, meaning that using heuristic methods such as the elbow method tends to be informative in choosing the number of features in covariate adjusted functional mixed membership models. Thus, we recommend using BIC and the elbow-method in conjunction to choose the number of features, as even though BIC correctly identified the model every time in the simulation, there were times when the BIC between the three-feature model and the four-feature model were similar. Additional details on how this simulation study was conducted can be found in Section 3.2 of the supplementary materials.

## 4. A Case Study in EEG Imaging for Autism Spectrum Disorder

**Figure 3.**
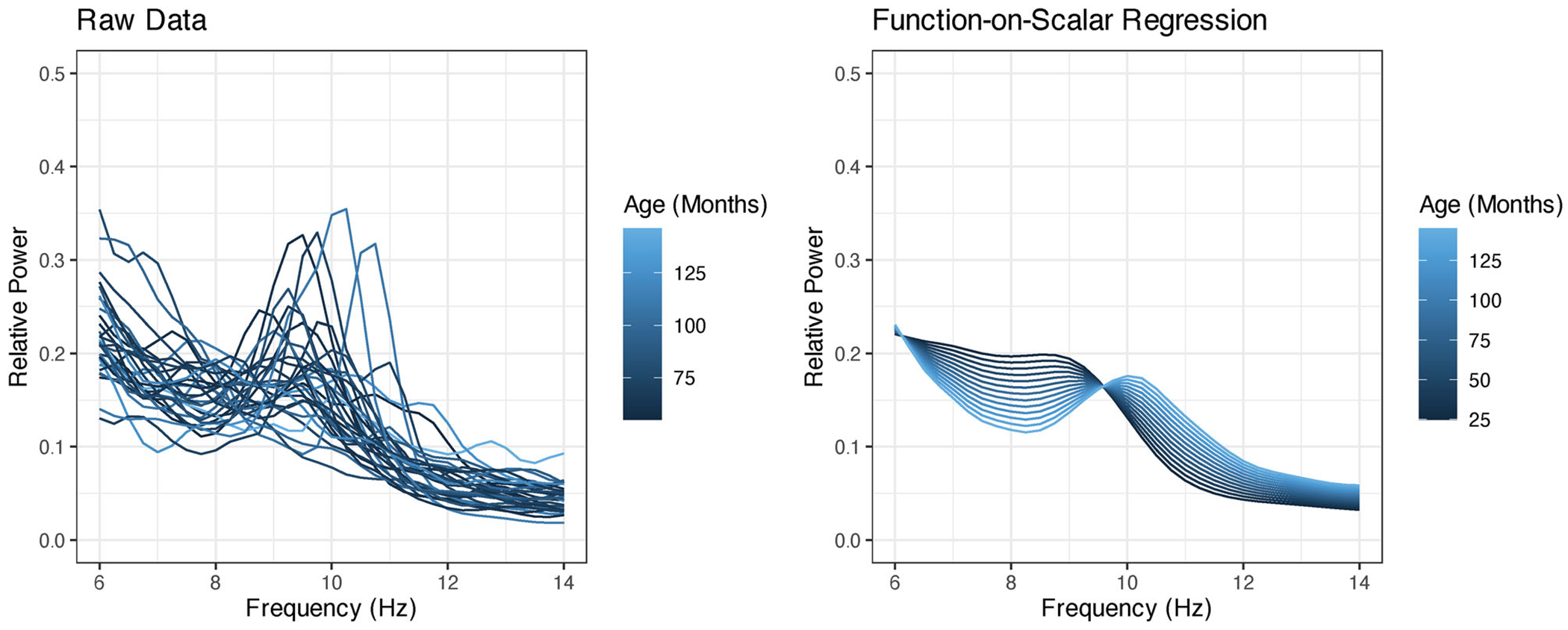
(Left Panel) Alpha frequency patterns averaged over the 25 channels for a sample of EEG recordings from 30 individuals (ASD and TD) with varying ages. (Right Panel) Estimated affects of age on alpha oscillations obtained by fitting a function-on-scalar model.

Autism spectrum disorder (ASD) is a developmental disorder characterized by social communication deficits and restrictive and/or repetitive behaviors (Association et al. 2013). Although once more narrowly defined, autism is now seen as a spectrum, with some individuals having very mild symptoms, to others that require lifelong support (Lord et al. 2018). In this case study, we analyze electroencephalogram (EEG) data that were obtained in a resting-state EEG study conducted by Dickinson et al. (2018). The study consisted of 58 children who have been diagnosed with ASD between the ages of 2 and 12 years of age, and 39 age-matched typically developing (TD) children, or children who have never been diagnosed with ASD. The children were instructed to view bubbles on a monitor in a dark, sound-attenuated room for 2 min, while EEG recordings were taken. The EEG recordings were obtained using a 128-channel HydroCel Geodesic Sensory net, and then interpolated to match the international 10–20 system 25-channel montage. The data were filtered using a band pass of 0.1–100 Hz, and then transformed into the frequency domain using a fast Fourier transform. To obtain the relative power, the functions were scaled so that they integrate to 1. Lastly, the relative power was averaged across the 25 channels to obtain a measure of the average relative power. Visualizations of the functional data that characterize the average relative power in the alpha band of frequencies can be seen in Figure 3.

In this case study, we are primarily interested in learning how alpha oscillations, which have been shown to play a role in neural coordination and communication between distributed brain regions (Fries 2005; Klimesch, Sauseng, and Hanslmayr 2007), differ between children with ASD and TD children. Due to the diverse age range of the children involved in the study, we define the alpha band as neural activity between 6Hz and 14Hz, which encompasses the lower frequency brain activity typically regarded as part of the alpha band in younger children (McLaughlin et al. 2011; Oberman, Ramachandran, and Pineda 2008).

Alpha oscillations are composed of periodic and aperiodic neural activity patterns that coexist in the EEG spectra. Neuroscientists are primarily interested in periodic signals, specifically in the location of a single prominent peak in the spectral density located in the alpha band of frequencies, called *peak alpha frequency* (PAF), which has been shown to be a biomarker of neural development in typically developing children (Rodríguez-Martínez et al. 2017). Studies have shown that the alpha peak becomes more prominent and shifts to a higher frequency within the first years of life for TD children (Rodríguez-Martínez et al. 2017; Scheffler et al. 2019). Compared to TD children, children with ASD tend to have a less attenuated alpha peak and a smaller developmental shift in the location of the alpha peak (Dickinson et al. 2018; Scheffler et al. 2019). However, these differences were found at the *population* level, leading to questions on whether (a) all TD children followed a similar developmental trajectory and (b) whether all children diagnosed with ASD have an atypical developmental trajectory compared to TD children. To answer these two questions, we first start by fitting a covariate adjusted functional mixed membership model in Section 4.1 with the log transformation of age as the only covariate. This model provides insight into how alpha frequencies change as children age, regardless of the diagnostic group. Additionally, the use of a covariate adjusted functional mixed membership model allows us to directly compare the age-adjusted relative strength of the PAF biomarker between individuals in the study, providing insight into how heterogeneous the developmental trajectories are between diagnostic groups. This analysis is extended in Section 4.2 where we fit a covariate adjusted functional mixed membership model using the log transformation of age, diagnostic group, and an interaction between the log transformation of age and diagnostic group as covariates. This analysis provides novel insight into whether there are differences in developmental trajectories between children with ASD and TD children. Although these two models may be considered nested models, each model provides unique insight into the data.

### 4.1. Modeling Alpha Oscillations Conditionally on Age

In the first part of this case study, we are primarily interested in gaining insight into the heterogeneity of developmental trajectories for both TD children and children with ASD. Although Marco et al. (2024b) provided some insight into the heterogeneity of alpha oscillations in children through the use of a functional mixed membership model, the model failed to account for developmental differences such as shifts in PAF, which can confound measurements of alpha power (Haegens et al. 2014). To gain this insight, we fit a covariate adjusted functional mixed membership model using the log transformation of age as the covariate. In this subsection, we present the results from a two-feature (*K* = 2) covariate adjusted functional mixed membership model, which was found to be the optimal number of features using AIC and BIC. Using conditional predictive ordinates and comparing the pseudomarginal likelihoods, we found that a log transformation of age provided a better fitting model than using a model with age untransformed. Although this model does not use information on the diagnostic group, we are also interested in characterizing the differences in the observed alpha oscillations between individuals across the diagnostic groups.

**Figure 4.**
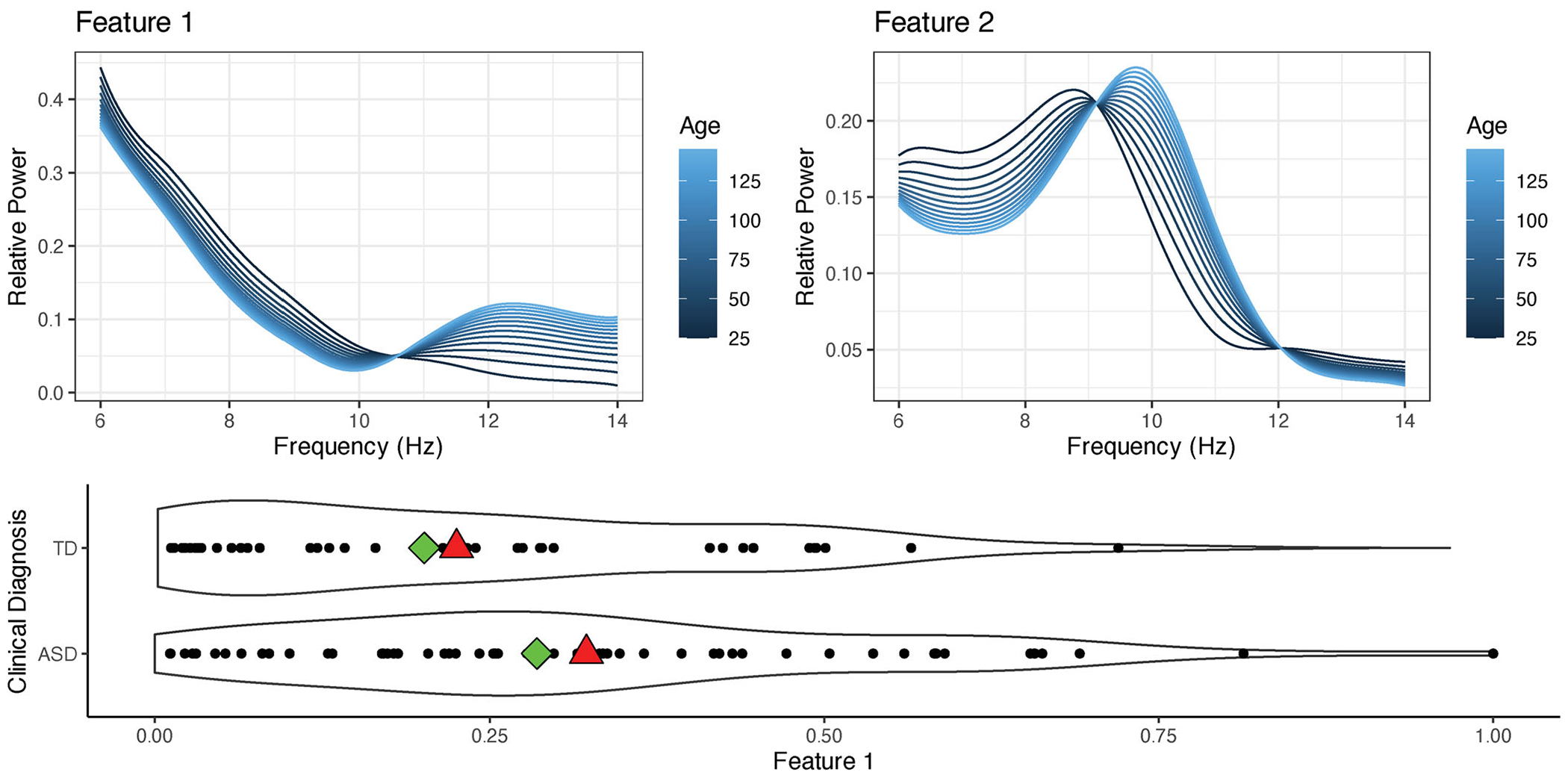
(Top Panels) Estimates of the mean functions of the two functional features conditional on Age. (Bottom Panel) Estimates of the allocation parameters found by fitting a covariate adjusted functional mixed membership model. Diagnostic group level means and medians of the allocation to the first feature is depicted as red triangles and green diamonds, respectively.

From Figure 4, we can see that the first feature consists mainly of aperiodic neural activity patterns, which are commonly referred to as a 1/*f* trend or pink noise, which stays consistently present as child develops. On the other hand, the second feature can be interpreted as a distinct alpha peak. For children that heavily load on the second feature, we can see that as they age, the alpha peak increases in magnitude and the PAF shifts to a higher frequency, which has been observed in many other studies (Haegens et al. 2014; Rodríguez-Martínez et al. 2017; Scheffler et al. 2019). As observed in previous studies (Dickinson et al. 2018; Scheffler et al. 2019), we can see that on average, TD children tend to load more heavily on feature 2 compared to children with ASD, which means that they tend to have a more attenuated alpha peak and a larger developmental shift in PAF. However, the allocation parameters also provide novel insight into the variability of developmental trajectories. Although there is more heterogeneity in the allocation parameters and, therefore, in the developmental trajectories of children with ASD, we can see that there is significant overlap in the distribution of allocation parameters between the two diagnostic groups. Therefore, there are some children with ASD that exhibit distinct alpha peaks comparable to most of their typically developing peers. Conversely, there are some TD children do not display any noticeable alpha peak; illustrating the considerable heterogeneity in developmental trajectories among both diagnostic groups. Although other variables such as gender, verbal IQ, and non-verbal IQ were not included as covariates due to the limited sample size, Section 3.3 of the supplementary materials contains visualizations of the association between the allocation parameters and these variables.

The flexibility of a covariate adjusted functional mixed membership model is particularly appealing in this case study, as it enables us to view individuals on a spectrum defined by the allocation parameters. Although a finite mixture of regressions model is similar to our proposed model, the model assumes that each individual belongs to only one cluster. However, scientifically, we know that alpha oscillations consist of a continuous mixture of periodic and aperiodic neural activity that coexists in the EEG spectra, making the mixed membership framework more appealing from a modeling perspective. The results of fitting a finite mixture of regressions model on this dataset can be found in Section 3.3 of the supplementary materials.

One key feature of this model is that it allows us to directly compare individuals in the TD group and the ASD group on the same spectrum. However, to do this, we had to assume that the alpha oscillations of children with ASD and TD children can be represented as a continuous mixture of the same two features shown in Figure 4. Just as developmental shifts in PAF can confound the measures of alpha power (Haegens et al. 2014), the assumption that the alpha oscillations of children with ASD and TD children can be represented by the same two features can also confound the results found in this section if the assumption is shown to be incorrect. In Section 4.2, we relax this assumption and allow the functional features to differ according to the diagnostic group.

### 4.2. Modeling Alpha Oscillations Conditionally on Age and Diagnostic Group

Previous studies have shown that, on average, the emergence of alpha peaks and the developmental shifts in frequency are atypical in children with ASD (Dickinson et al. 2018; Scheffler et al. 2019). Therefore, it may not be realistic to assume that the alpha oscillations of children with ASD and TD children can be represented by the same two features. In this section, we fit a covariate adjusted functional mixed membership model using the log transformation of age, clinical diagnosis, and an interaction between the log transformation of age and clinical diagnosis as covariates of interest. By including the interaction between the log transformation of age and the diagnostic group, we allow for differences in the developmental changes of the alpha oscillations between the diagnostic groups.

**Figure 5.**
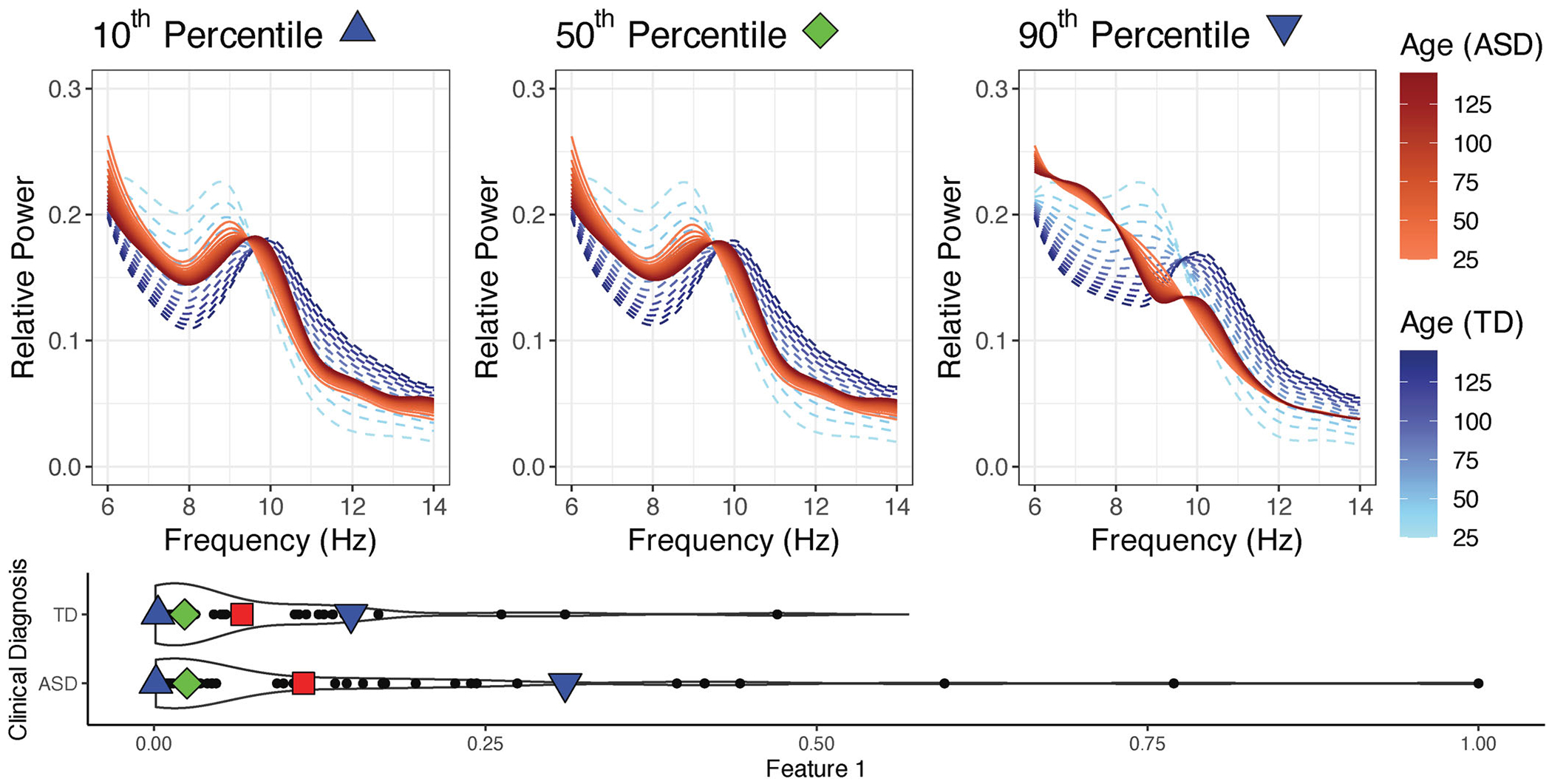
(Top Panels) Estimates of the developmental trajectories conditional on percentiles of the estimated allocation parameters by clinical diagnosis. (Bottom Panel) Estimates of the allocation parameters stratified by clinical diagnosis, with red squares representing the group-specific means, green diamonds representing the group-specific medians, blue triangles representing the group-specific 10th percentiles, and inverted blue triangles representing the group-specific 90th percentiles.

Compared to the model used in Section 4.1, this model is more flexible; it allows different features for each diagnostic group and different developmental trajectories for each diagnostic group. However, the comparison of individuals across diagnostic groups is no longer straightforward. The model in Section 4.1 did not use any diagnostic group information, which allowed us to easily compare TD children with ASD children as the distribution of the developmental trajectory depended only on the allocation parameters. By including diagnostic group as a covariate, we can no longer compare individuals across diagnostic groups by simply comparing the inferred allocation parameters, as the underlying features are group-specific. Specifically, two individuals with the same allocation parameters that are in different diagnostic groups will likely have completely different developmental trajectories. In addition, direct interpretation of the group-specific features is challenging, as the group-specific features can no longer be interpreted as the extreme cases within each diagnostic group. Indeed, from Figure 5, we can see that no individual in the TD group has allocation parameters that are more than 50% in feature 1. Therefore, the TD-specific feature 1 is challenging to interpret as it no longer characterizes the developmental trajectory of any subject in our study. Since we are interested in characterizing the differences in developmental trajectories of individuals conditionally on diagnostic group, we will focus on looking at the estimated trajectories of individuals conditionally on empirical percentiles of the estimated allocation structure. Here, we focus on visualizing the median trajectories for each diagnostic group, as well as the trajectory of individuals that fall in the first and last deciles. Although percentiles work well in the case where there are only two features (*K* = 2), one can employ a similar strategy when there are more features by evaluating the model at allocation parameters that summarize the distribution of the allocation parameters. Visualizations of the mean structure for the two features can be found in Section 3.3 of the supplementary materials.

Figure 5, shows the estimated developmental trajectories of TD children and children with ASD, conditional on different empirical percentiles of the estimated allocation structure. The results show that within each diagnostic group, the developmental trajectories of the individuals in the bottom 50th percentile are very similar. In particular, we can see that ASD children and TD children both have a discernible alpha peak. However, the alpha peak was more pronounced in TD children, and we see a larger shift in PAF for TD children compared to children with ASD. Alternatively, when looking at the 90th percentile for children diagnosed with ASD, we can see that there is no discernible alpha peak when the child is young, and an alpha peak never clearly develops as the child ages. Conversely, when looking at the 90th percentile of TD children, we can see that the child still develops a discernible alpha peak and that the PAF still shifts to the right as they age. However, compared to the 10th and 50th percentile, TD children in the 90th percentile have more aperiodic signal (1/*f* trend) present in their alpha oscillations. Similarly to Section 4.1, we can see that the distribution of allocation parameters for TD children has a long tail, indicating that there are still a few TD individuals that have primarily aperiodic signal and no discernible alpha peak. In general, these results are consistent with previous results (Rodríguez-Martínez et al. 2017; Dickinson et al. 2018; Scheffler et al. 2019) that for most TD children, the alpha peak becomes more prominent and the PAF shifts to higher frequencies as children age. However, these results provide new insight, suggesting that children with ASD in general appear to have smaller developmental changes in alpha oscillations as they age when compared to TD children.

**Figure 6.**
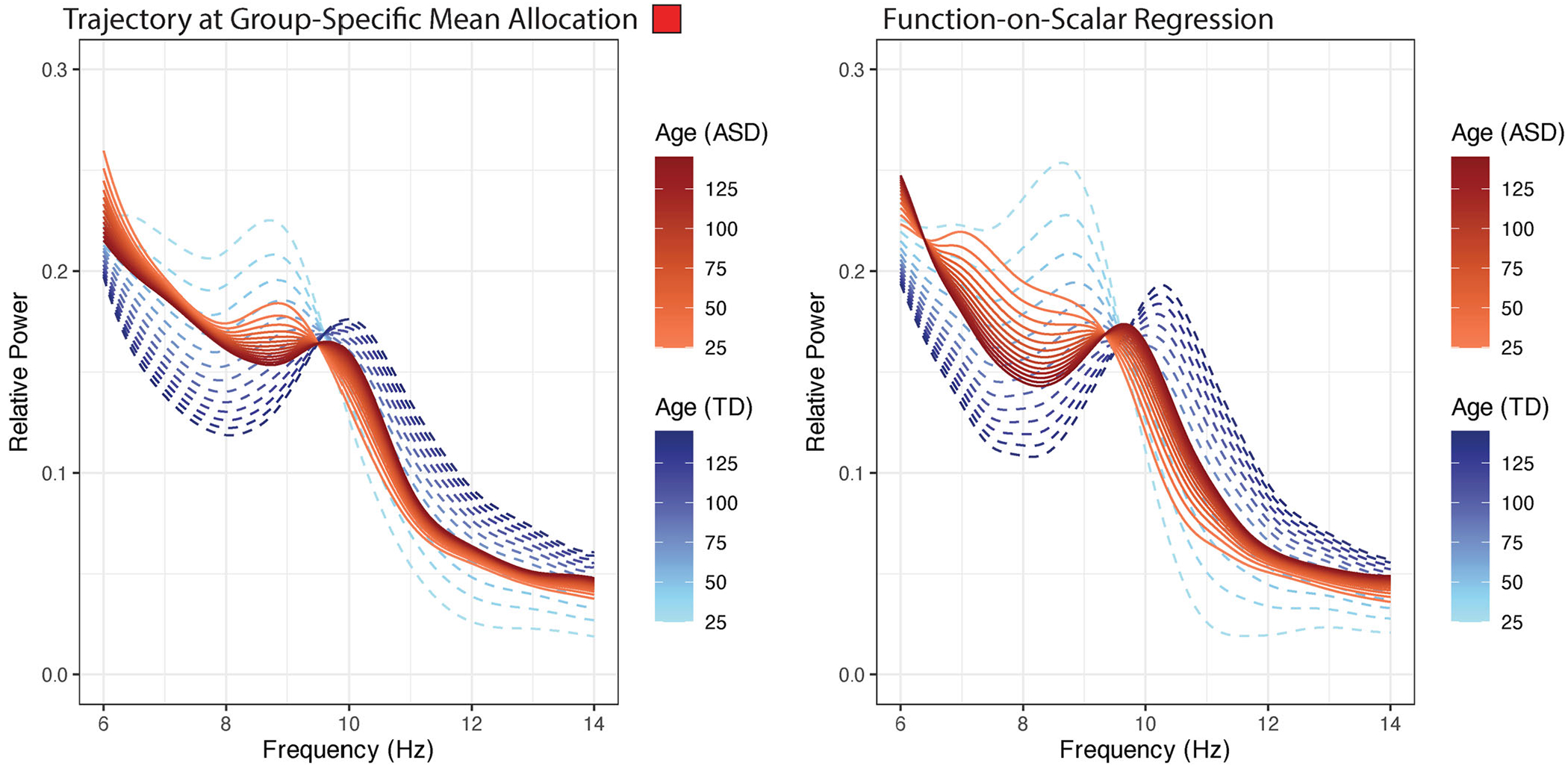
(Left Panel) Estimates of the developmental trajectories stratified by diagnostic group, conditional on the group-specific mean allocation (red squares in Figure 5) (Right panel) Estimated population-level development trajectories stratified by diagnostic group, obtained by fitting a function-on-scalar regression model.

The proposed covariate adjusted mixed membership model can be thought of as a generalization of function-on-scalar regression, where the covariate effects are allowed to vary based on the allocation parameters. Figure 6 contains the estimated developmental trajectories obtained by fitting a function-on-scalar regression model using the package “refund” package in R (Goldsmith et al. 2016). The results of the function-on-scalar regression generally coincide with the estimated developmental trajectory of the alpha oscillations obtained from our covariate adjusted mixed membership model, conditional on the group-specific mean allocation (represented by squares in the bottom panel of Figure 5). The major difference between the two results is the level of developmental changes between the two models, with function-on-scalar regression estimating larger developmental changes. This could possibly be due to the fact that function-on-scalar regression assumes a common mean for all individuals, controlling for age and diagnostic group. This is a relatively strong assumption in this case, as ASD is relatively broadly defined; with diagnosed individuals having varying degrees of symptoms. Indeed, from Figure 5, we can see that there is heterogeneity in the developmental trajectories in both diagnostic groups, with some individuals having large differences in developmental trajectories as evident by the long tails in the distribution of the allocation parameters. Function-on-scalar regression cannot account for this type of heterogeneity and is only capable of making population-level inference; providing no insight into the heterogeneity of the developmental trajectories of alpha oscillations for individual children. Technical details on how the models for Sections 4.1 and 4.2 were fit can be found in Section 3.3 of the supplementary materials. Section 3.4 of the supplementary materials also discusses model comparison using conditional predictive ordinates (Pettit 1990; Chen, Shao, and Ibrahim 2012; Lewis et al. 2014) and pseudomarginal likelihood.

## 5. Discussion

In this article, we extend the framework of the functional mixed membership model proposed by Marco et al. (2024b) to allow for covariate dependence. This work was primarily motivated by a neurodevelopmental EEG study on alpha oscillations, where alpha oscillations are known to change as children age. Although mixed membership models provided a novel way to quantify the emergence and presence of a developmental biomarker known as an alpha peak (Marco et al. 2024a, 2024b), it has been shown that not accounting for developmental shifts in the alpha peak can confound measures of peak alpha frequency (Haegens et al. 2014), leading to the need for a covariate adjusted functional mixed membership model. Using our proposed framework, we were able to confirm that, as children with ASD age, the emergence of alpha peaks and the developmental shifts in frequency are less apparent in children with ASD when compared to their typically developing counterparts (Dickinson et al. 2018; Scheffler et al. 2019). However, compared to regression based frameworks or finite mixture frameworks, we were able to get additional insight into the level of heterogeneity of the developmental changes. As illustrated in Figure 5, we saw that individuals with ASD had smaller developmental changes compared to their typically developing counterpart. Specifically, children with ASD that have discernible alpha peaks tended to have them from a young age with very little change in alpha power as the child ages. Alternatively, children with ASD who did not have a discernible alpha peak never seemed to develop an alpha peak as they age. Compared to typical regression models, the covariate adjusted mixed membership models allowed us to capture heterogeneity in the covariate effects, while not making a strong assumption that each observation belongs to a single cluster, as is typically done in finite mixture models. Additionally, the Bayesian framework allows us to make use of posterior predictive distributions, allowing us to infer the future developmental changes in alpha frequencies for any individual.

Although the proposed model was introduced for an arbitrary number of features, and Lemma 2.1 holds for an arbitrary number of features, there are computational challenges to working with models with more than three features. As discussed in Section 2.4, we typically have to assume the separability condition or the sufficiently scattered condition. In a two-feature model, these two assumptions are equivalent and can be enforced by post-processing the Markov chain constructed through MCMC. When considering a model with three or more features, the separability condition becomes a relatively strong geometric assumption on the generating process, making the sufficiently scattered condition more theoretically appealing. For a three-feature model, we can similarly post-process the Markov chain using the work of Pârvu and Gilbert (2016) to arrive at posterior samples that satisfy the sufficiently scattered assumption. However, ensuring that the sufficiently scattered assumption holds for models with four or more features is still an open problem. Similarly, more work is needed to derive a sampler that obeys the geometric constraints set forth by the sufficiently scattered condition.

The proposed covariate adjusted functional mixed membership model is closely related to function-on-scalar regression (Ramsay and Silverman 2005a; Morris 2015) and finite mixture of regressions. Specifically, the covariate adjusted functional mixed membership model can be viewed as a generalization of a functional finite mixture of regressions model, which itself can be viewed as a generalization of function-on-scalar regression. The simplest model, function-on-scalar regression, assumes a common mean and covariance structure across all functional observations conditionally on the covariates of interest, allowing for *population*-level inference. The functional finite mixture of regressions model can be viewed as a more granular version of function-on-scalar regression, permitting *subpopulation*-level inference. The finite mixture of regressions framework assumes that observations in the same cluster share a common mean and covariance structure conditionally on the covariates of interest, but allow the mean and covariance structure to differ across clusters. The covariate adjusted functional mixed membership model can be viewed as the most granular model among the three frameworks, allowing for *observation*-level inference. Specifically, the proposed model allows for the covariate effects, mean, and covariance structures to differ across observations depending on the allocation parameters. The model assumes that each functional observation can be represented by a convex combination of dependent latent features. Although both the finite mixture of regressions framework and covariate adjusted mixed membership framework rely on the assumption of *K* underlying features, the continuous nature of the allocation parameters, along with dependent features in the covariate adjusted mixed membership models, provide a significantly more flexible model. A comprehensive review of function-on-scalar regression, as well as its relationship with the proposed functional covariate adjusted mixed membership model, can be found in Section 5 of the supplementary materials.

Although functional-on-scalar regression can typically handle a large number of covariates, with regularized function-on-scalar regression (Fan and Reimherr 2017; Kowal and Bourgeois 2020) becoming more popular, the model proposed in this article is most appropriate when interest centers on low-dimensional covariate information. Specifically, as seen in Section 3.1, we need significantly more observations as we add covariates and lose statistical efficiency when fitting an over-specified model. While one may be tempted to use information criteria to perform variable selection, we urge caution, as the addition of additional covariates can greatly impact the interpretation of the allocation parameters, as seen in Section 4.2. Thus, the incorporation of a covariate in the model should be decided based on the scientific goals, prior knowledge of how the covariates affect the observed functions, and computational/statistical efficiency considerations. The R package BayesFMMM is available on Github to fit the proposed covariate adjusted functional mixed membership model.

## Supplementary Material

Supp 1

Supplementary materials for this article are available online. Please go to www.tandfonline.com/r/JSDI.

**Supplementary Materials:** Contains proofs for all lemmas in the article, details on posterior computation, additional details for the case studies and simulation studies, and a covariate adjusted model where the mean and covariance structures depend on the covariates of interest.

**BayesFMMM:** The R package to fit the proposed covariate adjusted functional mixed membership model can be found on GitHub at https://github.com/ndmarco/BayesFMMM.

**Simulation Studies and Case Studies:** The R scripts for the simulation and case studies can be found on GitHub at https://github.com/ndmarco/BFMMM_CA_Functional_Sims. Unfortunately, the data for the EEG case study cannot be made publicly available.
