# Supplementary material for: Covariate Adjusted Functional Mixed Membership Models
Source: Stat Data Sci Imaging. Author manuscript; Available in PMC 2025 Nov 13. (PMC12610336; doi:10.1080/29979676.2025.2566646)
Supplement: Supp 1 [file NIHMS2121187-supplement-Supp_1.pdf]

Supplementary Materials for “Covariate Adjusted Functional Mixed Membership Models”

September 22, 2025

Abstract

Section 1 of the Supplementary Materials contains the proof for Lemma 2.1. Section 2 contains details on the posterior distributions, as well as the outline for a tempered transition MCMC scheme. Section 3 contains additional details on the simulation studies and case studies presented in the main manuscript. Section 4 contains details for a covariate adjusted model where the mean and covariance structure depend on the covariates of interest. Lastly, Section 5 contains a detailed discussion on the relationship of the proposed covariate adjusted functional mixed membership and function-on-scalar regression.

1 Proof of Lemma 2.1

We will start by defining identifiability and defining some of the notation used in this section. Let $\boldsymbol{\omega} = \{\boldsymbol{\nu}_1, \dots, \boldsymbol{\nu}_K, \boldsymbol{\eta}_1, \dots, \boldsymbol{\eta}_K, \{Z_{j1}, \dots, Z_{jK}\}_{j=1}^N, \{\boldsymbol{\Sigma}_{jk}\}_{1\le j\le k\le K}, \sigma^2\}$, where $\boldsymbol{\Sigma}_{jk} = \sum_{m=1}^M \left(\boldsymbol{\phi}_{jm}\boldsymbol{\phi}_{km}^\intercal\right)$. We will say that the parameters $\boldsymbol{\omega}$ are unidentifiable if there exists at least one $\boldsymbol{\omega}^* \neq \boldsymbol{\omega}$ such that $\mathcal{L}(\mathbf{Y}_i(\mathbf{t}_i) \mid \boldsymbol{\omega}, \mathbf{x}_i) = \mathcal{L}(\mathbf{Y}_i(\mathbf{t}_i) \mid \boldsymbol{\omega}^*, \mathbf{x}_i)$ for all sets of observations $\{\mathbf{Y}_i(\mathbf{t}_i)\}_{i=1}^N$ following Assumptions (1)-(3). Otherwise, the parameters $\boldsymbol{\omega}$ are called identifiable. In this case, $\mathcal{L}(\mathbf{Y}_i(\mathbf{t}_i) \mid \boldsymbol{\omega}, \mathbf{x}_i)$ is the likelihood specified in Equation 9 in the main text.

From Equation 9 in the main text, we have that

$$\mathcal{L}\left(\mathbf{Y}_i(\mathbf{t}_i) \mid \boldsymbol{\omega}, \mathbf{x}_i\right) \propto \exp\left\{-\tfrac{1}{2}\left(\mathbf{Y}_i(\mathbf{t}_i) - \mu_i(\mathbf{x}_i, \mathbf{t}_i)\right)^\intercal \left(\mathbf{V}(\mathbf{t}_i, \mathbf{z}_i) + \sigma^2\mathbf{I}_{n_i}\right)^{-1} \left(\mathbf{Y}_i(\mathbf{t}_i) - \mu_i(\mathbf{x}_i, \mathbf{t}_i)\right)\right\}, \quad (1)$$

where

$$\mu_i(\mathbf{x}_i, \mathbf{t}_i) = \sum_{k=1}^K Z_{ik}\mathbf{S}^\intercal(\mathbf{t}_i)\left(\boldsymbol{\nu}_k + \boldsymbol{\eta}_k\mathbf{x}_i^\intercal\right)$$

and

$$\mathbf{V}(\mathbf{t}_i, \mathbf{z}_i) = \sum_{k=1}^K\sum_{k'=1}^K Z_{ik}Z_{ik'}\left\{\mathbf{S}(\mathbf{t}_i)\sum_{m=1}^M\left(\boldsymbol{\phi}_{km}\boldsymbol{\phi}_{k'm}^\intercal\right)\mathbf{S}(\mathbf{t}_i)\right\}.$$

Assume that $\mathcal{L}\left(\mathbf{Y}_i(\mathbf{t}_i) \mid \boldsymbol{\omega}, \mathbf{x}_i\right) = \mathcal{L}\left(\mathbf{Y}_i(\mathbf{t}_i) \mid \boldsymbol{\omega}^*, \mathbf{x}_i\right)$ for all sets of observations $\{\mathbf{Y}_i(\mathbf{t}_i)\}_{i=1}^N$ that follow Assumptions (1)-(3) . Thus we would like to prove that $\boldsymbol{\omega}^* = \boldsymbol{\omega}$ must necessarily be true. Since $\mathcal{L}\left(\mathbf{Y}_i(\mathbf{t}_i) \mid \boldsymbol{\omega}, \mathbf{x}_i\right)$ is written as a quadratic form in $\mathbf{Y}_i(\mathbf{t}_i)$ and $(\mathbf{V}(\mathbf{t}_i, \mathbf{z}_i) + \sigma^2\mathbf{I}_{n_i})$ is full rank, we see that the following must necessarily be true:

1. $\mu_i^*(\mathbf{x}_i, \mathbf{t}_i) = \mu_i(\mathbf{x}_i, \mathbf{t}_i)$,

2. $\mathbf{V}^*(\mathbf{t}_i, \mathbf{z}_i^*) + (\sigma^2)^*\mathbf{I}_{n_i} = \mathbf{V}(\mathbf{t}_i, \mathbf{z}_i) + \sigma^2\mathbf{I}_{n_i}$,

for $i = 1, \dots, N$. By (1), we have that

$$\sum_{k=1}^K Z_{ik}\mathbf{S}^\intercal(\mathbf{t}_i)\left(\boldsymbol{\nu}_k + \boldsymbol{\eta}_k\mathbf{x}_i^\intercal\right) = \sum_{k=1}^K Z_{ik}^*\mathbf{S}^\intercal(\mathbf{t}_i)\left(\boldsymbol{\nu}_k^* + \boldsymbol{\eta}_k^*\mathbf{x}_i^\intercal\right) \quad (i = 1, \dots, N).$$

Letting $\boldsymbol{\mu}_k = [\boldsymbol{\nu}_k\ \boldsymbol{\eta}_k] \in \mathbb{R}^{P\times(R+1)}$ and $\tilde{\mathbf{x}}_i = [1\ \mathbf{x}_i]$ ($\tilde{\mathbf{X}} \in \mathbb{R}^{N\times(R+1)}$ is the design matrix with the ith

row as $\tilde{\mathbf{x}}$), we have

$$\sum_{k=1}^{K} Z_{ik}\mathbf{S}^{\intercal}(\mathbf{t}_i)\boldsymbol{\mu}_k\tilde{\mathbf{x}}_i^{\intercal} = \sum_{k=1}^{K} Z_{ik}^{*}\mathbf{S}^{\intercal}(\mathbf{t}_i)\boldsymbol{\mu}_k^{*}\tilde{\mathbf{x}}_i^{\intercal} \quad (i = 1,\dots,N)$$

$$\iff \sum_{k=1}^{K} Z_{ik}\boldsymbol{\mu}_k\tilde{\mathbf{x}}_i^{\intercal} = \sum_{k=1}^{K} Z_{ik}^{*}\boldsymbol{\mu}_k^{*}\tilde{\mathbf{x}}_i^{\intercal} \quad (i = 1,\dots,N), \tag{2}$$

since $n_i \geq P$ by Assumption (3). Since $\tilde{\mathbf{X}}$ is full column rank from Assumption (1), we have that

$$\sum_{k=1}^{K} Z_{ik}\boldsymbol{\mu}_k = \sum_{k=1}^{K} Z_{ik}^{*}\boldsymbol{\mu}_k^{*} \quad (i = 1,\dots,N). \tag{3}$$

It is important to note that if Assumption (1) does not hold and $\tilde{\mathbf{X}}$ is not full column rank, we could add any vector in the nullspace of $\tilde{\mathbf{X}}$ to any row of $\boldsymbol{\mu}_k^{*}$ $(k = 1,\dots,K)$ and Equation 2 would still hold.

We can rewrite Equation 3 in matrix form such that

$$\mathbf{Z}\tilde{\boldsymbol{\mu}} = \mathbf{Z}^{*}\tilde{\boldsymbol{\mu}}^{*} \tag{4}$$

where $\tilde{\boldsymbol{\mu}}_r = [\text{vec}(\boldsymbol{\mu}_1),\dots,\text{vec}(\boldsymbol{\mu}_K)]^{\intercal} \in \mathbb{R}^{K\times P(R+1)}$ and $\mathbf{Z} \in \mathbb{R}^{N\times K}$ is the matrix of allocation parameters with $\mathbf{z}_i$ as the ith row of $\mathbf{Z}$. From this we can directly apply the results of Chen et al. (2022), to show that $\mathbf{Z} = \mathbf{Z}^{*}$ and $\tilde{\boldsymbol{\mu}} = \tilde{\boldsymbol{\mu}}^{*}$ up to a permutation of the labels. Specifically, if the separability condition holds, then Proposition 1 of Chen et al. (2022) shows that $\mathbf{Z} = \mathbf{Z}^{*}$ and $\tilde{\boldsymbol{\mu}} = \tilde{\boldsymbol{\mu}}^{*}$ up to a permutation of the labels. If the sufficiently scattered condition holds, then Theorem 2 of Chen et al. (2022) shows that $\mathbf{Z} = \mathbf{Z}^{*}$ and $\tilde{\boldsymbol{\mu}} = \tilde{\boldsymbol{\mu}}^{*}$ up to a permutation of the labels. Therefore, assuming that Assumptions (1) - (3) hold, we have $Z_{ik} = Z_{ik}^{*}$, $\boldsymbol{\nu}_k = \boldsymbol{\nu}_k^{*}$, and $\boldsymbol{\eta}_k = \boldsymbol{\eta}_k^{*}$ up to the permutation of the labels, for $k = 1, 2$ and $i = 1,\dots,N$.

From (2), we have that

$$\mathbf{V}^{*}(\mathbf{t}_i, \mathbf{z}_i^{*}) + (\sigma^2)^{*}\mathbf{I}_{n_i} = \mathbf{V}(\mathbf{t}_i, \mathbf{z}_i) + \sigma^2\mathbf{I}_{n_i}$$

$$\iff \mathbf{V}^{*}(\mathbf{t}_i, \mathbf{z}_i^{*}) - \mathbf{V}(\mathbf{t}_i, \mathbf{z}_i) = ((\sigma^2)^{*} - \sigma^2)\mathbf{I}_{n_i}.$$

Suppose that $((\sigma^2)^{*} - \sigma^2) \neq 0$, then we have that

$$\text{rank}\left(\mathbf{V}^{*}(\mathbf{t}_i, \mathbf{z}_i^{*}) - \mathbf{V}(\mathbf{t}_i, \mathbf{z}_i)\right) = \text{rank}\left(((\sigma^2)^{*} - \sigma^2)\mathbf{I}_{n_i}\right) > P,$$

by Assumption (3) $(n_i > P)$. Writing $\mathbf{V}(\mathbf{t}_i, \mathbf{z}_i)$ such that

$$\mathbf{V}(\mathbf{t}_i, \mathbf{z}_i) = \mathbf{S}^\intercal(\mathbf{t}_i)\left\{\sum_{k=1}^{K}\sum_{k'=1}^{K} Z_{ik}Z_{ik'}\sum_{m=1}^{M}\left(\boldsymbol{\phi}_{km}\boldsymbol{\phi}_{k'm}^\intercal\right)\right\}\mathbf{S}(\mathbf{t}_i),$$

we can see that $\text{rank}(\mathbf{V}(\mathbf{t}_i, \mathbf{z}_i)) \leq P$, which implies that $\text{rank}\left(\mathbf{V}^*(\mathbf{t}_i, \mathbf{z}_i^*) - \mathbf{V}(\mathbf{t}_i, \mathbf{z}_i)\right) \leq P$, leading to a contradiction. Therefore, we have $(\sigma^2)^* = \sigma^2$ and $\mathbf{V}^*(\mathbf{t}_i, \mathbf{z}_i^*) = \mathbf{V}(\mathbf{t}_i, \mathbf{z}_i)$ up to a permutation of the labels. Assuming no permutation of the labels ($\mathbf{Z} = \mathbf{Z}^*$), we have that

$$\mathbf{V}(\mathbf{t}_i, \mathbf{z}_i) = \mathbf{V}^*(\mathbf{t}_i, \mathbf{z}_i^*) \iff \sum_{k=1}^{K}\sum_{k'=1}^{K} Z_{ik}Z_{ik'}\boldsymbol{\Sigma}_{kk'} = \sum_{k=1}^{K}\sum_{k'=1}^{K} Z_{ik}Z_{ik'}\boldsymbol{\Sigma}_{kk'}^*,$$

since $n_i > P$ for all i. Therefore, we have the following system of equations

$$\sum_{k=1}^{K} Z_{ik}^2\left(\boldsymbol{\Sigma}_{kk} - \boldsymbol{\Sigma}_{kk}^*\right) + 2\sum_{k=1}^{K}\sum_{k'>k} Z_{ik}Z_{ik'}\left(\boldsymbol{\Sigma}_{kk'} - \boldsymbol{\Sigma}_{kk'}^*\right) = \mathbf{0},$$

for $i = 1, \dots, N$. Equality can be proved if we have $\frac{K^2+K}{2}$ linearly independent equations in our system of equations. Thus, if the coefficient matrix has rank $\frac{K^2+K}{2}$, then we have $\frac{K^2+K}{2}$ linearly independent equations in our system of equations. We can see that Assumption (2) gives us that the coefficient matrix, denoted $\mathbf{C}$, has full column rank, meaning that we have $\frac{K^2+K}{2}$ linearly independent equations in our system of equations. Therefore, we have that $\boldsymbol{\Sigma}_{kk'} = \boldsymbol{\Sigma}_{kk'}^*$ for $1 \leq k, k' \leq K$, up to a permutation of the labels. Therefore, we have that for $i = 1, \dots, N$ and $k, k' = 1, \dots, K$, the parameters $\boldsymbol{\nu}_k$, $\boldsymbol{\eta}_k$, Z_{ik}, $\sum_{m=1}^{M}\left(\boldsymbol{\phi}_{km}\boldsymbol{\phi}_{k'm}^\intercal\right)$, and σ^2 are identifiable up to a permutation of the labels given Assumptions (1)-(3).

2 Computation

2.1 Posterior Distributions

In this subsection, we will specify the posterior distributions specifically for the functional covariate adjusted mixed membership model proposed in the main manuscript. We will first start with the $\boldsymbol{\phi}_{km}$ parameters, for $j = 1, \dots, K$ and $m = 1, \dots, M$. Let $\mathbf{D}_{\phi_{jm}} = \tilde{\tau}_{\phi_{mj}}^{-1} diag\left(\gamma_{\phi_{j1m}}^{-1}, \dots, \gamma_{\phi_{jPm}}^{-1}\right)$. By

letting

$$\mathbf{m}_{\phi_{jm}} = \frac{1}{\sigma^2}\sum_{i=1}^{N}\sum_{l=1}^{n_i}\Bigg(B(t_{il})\chi_{im}\Bigg(y_i(t_{il})Z_{ij} - Z_{ij}^2\left(\boldsymbol{\nu}_j + \boldsymbol{\eta}_j\mathbf{x}_i^\intercal\right)^\intercal B(t_{il}) - Z_{ij}^2\sum_{n\neq m}\chi_{in}\boldsymbol{\phi}_{jn}^\intercal B(t_{il})$$
$$- \sum_{k\neq j} Z_{ij}Z_{ik}\left[(\boldsymbol{\nu}_k + \boldsymbol{\eta}_k\mathbf{x}_i^\intercal)^\intercal B(t_{il}) + \sum_{n=1}^{M}\chi_{in}\boldsymbol{\phi}_{kn}^\intercal B(t_{il})\right]\Bigg)\Bigg),$$

and

$$\mathbf{M}_{\phi_{jm}}^{-1} = \frac{1}{\sigma^2}\sum_{i=1}^{N}\sum_{l=1}^{n_i}\left(Z_{ij}^2\chi_{im}^2 B(t_{il})B^\intercal(t_{il})\right) + \mathbf{D}_{\phi_{jm}}^{-1},$$

we have that

$$\boldsymbol{\phi}_{jm}|\boldsymbol{\Theta}_{-\phi_{jm}}, \mathbf{Y}_1, \ldots, \mathbf{Y}_N, \mathbf{X} \sim \mathcal{N}\left(\mathbf{M}_{\phi_{jm}}\mathbf{m}_{\phi_{jm}}, \mathbf{M}_{\phi_{jm}}\right).$$

The posterior distribution of δ_{1k}, for $k = 1, \ldots, K$, is

$$\delta_{1k}|\boldsymbol{\Theta}_{-\delta_{1k}}, \mathbf{Y}_1, \ldots, \mathbf{Y}_N, \mathbf{X} \sim \Gamma\Bigg(a_{1k} + (PM/2), 1 + \frac{1}{2}\sum_{r=1}^{P}\gamma_{k,r,1}\phi_{k,r,1}^2$$
$$+ \frac{1}{2}\sum_{m=2}^{M}\sum_{r=1}^{P}\gamma_{k,r,m}\phi_{k,r,m}^2\left(\prod_{j=2}^{m}\delta_{jk}\right)\Bigg).$$

The posterior distribution for δ_{ik}, for $i = 2, \ldots, M$ and $k = 1, \ldots, K$, is

$$\delta_{ik}|\boldsymbol{\Theta}_{-\delta_{ik}}, \mathbf{Y}_1, \ldots, \mathbf{Y}_N, \mathbf{X} \sim \Gamma\Bigg(a_{2k} + (P(M-i+1)/2), 1$$
$$+ \frac{1}{2}\sum_{m=i}^{M}\sum_{r=1}^{P}\gamma_{\boldsymbol{\xi}_{k,r,m}}\phi_{k,r,m}^2\left(\prod_{j=1;j\neq i}^{m}\delta_{jk}\right)\Bigg).$$

The posterior distribution for a_{1k} $(k = 1, \ldots, K)$ is not a commonly known distribution, however we have that

$$P(a_{1k}|\boldsymbol{\Theta}_{-a_{1k}}, \mathbf{Y}_1, \ldots, \mathbf{Y}_N, \mathbf{X}) \propto \frac{1}{\Gamma(a_{1k})}\delta_{1k}^{a_{1k}-1}a_{1k}^{\alpha_1 - 1}exp\left\{-a_{1k}\beta_1\right\}.$$

Since this is not a known kernel of a distribution, we will have to use Metropolis-Hastings algorithm. Consider the proposal distribution $Q(a_{1k}'|a_{1k}) = \mathcal{N}\left(a_{1k}, \epsilon_1\beta_1^{-1}, 0, +\infty\right)$ (Truncated Normal) for some small $\epsilon_1 > 0$. Thus the probability of accepting any step is

$$A(a_{1k}', a_{1k}) = \min\left\{1, \frac{P\left(a_{1k}'|\boldsymbol{\Theta}_{-a_{1k}'}, \mathbf{Y}_1, \ldots, \mathbf{Y}_N, \mathbf{X}\right)}{P\left(a_{1k}|\boldsymbol{\Theta}_{-a_{1k}}, \mathbf{Y}_1, \ldots, \mathbf{Y}_N, \mathbf{X}\right)}\frac{Q\left(a_{1k}|a_{1k}'\right)}{Q\left(a_{1k}'|a_{1k}\right)}\right\}.$$

Similarly for a_{2k} $(k = 1, \dots, K)$, we have

$$P(a_{2k}|\mathbf{\Theta}_{-a_{2k}}, \mathbf{Y}_1, \dots, \mathbf{Y}_N, \mathbf{X}) \propto \frac{1}{\Gamma(a_{2k})^{M-1}} \left(\prod_{i=2}^{M} \delta_{ik}^{a_{2k}-1}\right) a_{2k}^{\alpha_{2k}-1} exp\left\{-a_{2k}\beta_2\right\}.$$

We will use a similar proposal distribution, such that $Q(a'_{2k}|a_{2k}) = \mathcal{N}\left(a_{2k}, \epsilon_2\beta_2^{-1}, 0, +\infty\right)$ for some small $\epsilon_2 > 0$. Thus, the probability of accepting any step is

$$A(a'_{2k}, a_{2k}) = \min\left\{1, \frac{P\left(a'_{2k}|\mathbf{\Theta}_{-a'_{2k}}, \mathbf{Y}_1, \dots, \mathbf{Y}_N, \mathbf{X}\right)}{P\left(a_{2k}|\mathbf{\Theta}_{-a_{2k}}, \mathbf{Y}_1, \dots, \mathbf{Y}_N, \mathbf{X}\right)} \frac{Q\left(a_{2k}|a'_{2k}\right)}{Q\left(a'_{2k}|a_{2k}\right)}\right\}.$$

The posterior distribution for the $\mathbf{z}_i$ parameters are not a commonly known distribution, so we will use the Metropolis-Hastings algorithm. We know that

$$\begin{aligned} p(\mathbf{z}_i|\mathbf{\Theta}_{-\mathbf{z}_i}, \mathbf{Y}_1, \dots, \mathbf{Y}_N, \mathbf{X}) \propto & \prod_{k=1}^{K} Z_{ik}^{\alpha_3\pi_k-1} \\ & \times \prod_{l=1}^{n_i} exp\left\{-\frac{1}{2\sigma^2}\left(y_i(t_{il}) - \sum_{k=1}^{K} Z_{ik}\left((\boldsymbol{\nu}_k + \boldsymbol{\eta}_k\mathbf{x}_i^\intercal)^\intercal B(t_{il})\right.\right.\right. \\ & \left.\left.\left. + \sum_{m=1}^{M}\chi_{im}\boldsymbol{\phi}_{km}^\intercal B(t_{il})\right)\right)^2\right\}. \end{aligned}$$

We will use $Q(\mathbf{z}'_i|\mathbf{z}_i) = Dir(a_{\mathbf{z}}\mathbf{z}_i)$ for some large $a_{\mathbf{z}} \in \mathbb{R}^+$ as the proposal distribution. Thus the probability of accepting a proposed step is

$$A(\mathbf{z}'_i, \mathbf{z}_i) = \min\left\{1, \frac{P\left(\mathbf{z}'_i|\mathbf{\Theta}_{-\mathbf{z}_i}, \mathbf{Y}_1, \dots, \mathbf{Y}_N, \mathbf{X}\right)}{P\left(\mathbf{z}_i|\mathbf{\Theta}_{-\mathbf{z}_i}, \mathbf{Y}_1, \dots, \mathbf{Y}_N, \mathbf{X}\right)} \frac{Q\left(\mathbf{z}_i|\mathbf{z}'_i\right)}{Q\left(\mathbf{z}'_i|\mathbf{z}_i\right)}\right\}.$$

Similarly, a Gibbs update is not available for an update of the $\boldsymbol{\pi}$ parameters. We have that

$$\begin{aligned} p(\boldsymbol{\pi}|\mathbf{\Theta}_{-\boldsymbol{\pi}}, \mathbf{Y}_1, \dots, \mathbf{Y}_N, \mathbf{X}) \propto & \prod_{k=1}^{K} \pi_k^{c_k-1} \\ & \times \prod_{i=1}^{N} \frac{1}{B(\alpha_3\boldsymbol{\pi})} \prod_{k=1}^{K} Z_{ik}^{\alpha_3\pi_k-1}. \end{aligned}$$

Letting our proposal distribution be such that $Q(\boldsymbol{\pi}'|\boldsymbol{\pi}) = Dir(a_{\boldsymbol{\pi}}\boldsymbol{\pi})$, for some large $a_{\boldsymbol{\pi}} \in \mathbb{R}^+$, we have that our probability of accepting any proposal is

$$A(\boldsymbol{\pi}', \boldsymbol{\pi}) = \min\left\{1, \frac{P\left(\boldsymbol{\pi}'|\mathbf{\Theta}_{-\boldsymbol{\pi}'}, \mathbf{Y}_1, \dots, \mathbf{Y}_N, \mathbf{X}\right)}{P\left(\boldsymbol{\pi}|\mathbf{\Theta}_{-\boldsymbol{\pi}}, \mathbf{Y}_1, \dots, \mathbf{Y}_N, \mathbf{X}\right)} \frac{Q\left(\boldsymbol{\pi}|\boldsymbol{\pi}'\right)}{Q\left(\boldsymbol{\pi}'|\boldsymbol{\pi}\right)}\right\}.$$

The posterior distribution of α_3 is also not a commonly known distribution, so we will use the Metropolis-Hastings algorithm to sample from the posterior distribution. We have that

$$p(\alpha_3|\boldsymbol{\Theta}_{-\alpha_3}, \mathbf{Y}_1, \dots, \mathbf{Y}_N, \mathbf{X}) \propto e^{-b\alpha_3} \times \prod_{i=1}^{N} \frac{1}{B(\alpha_3 \boldsymbol{\pi})} \prod_{k=1}^{K} Z_{ik}^{\alpha_3 \pi_k - 1}.$$

Using a proposal distribution such that $Q(\alpha_3'|\alpha_3) = \mathcal{N}(\alpha_3, \sigma^2_{\alpha_3}, 0, +\infty)$ (Truncated Normal), we are left with the probability of accepting a proposed state as

$$A(\alpha_3', \alpha_3) = \min\left\{1, \frac{P\left(\alpha_3'|\boldsymbol{\Theta}_{-\alpha_3'}, \mathbf{Y}_1, \dots, \mathbf{Y}_N, \mathbf{X}\right)}{P\left(\alpha_3|\boldsymbol{\Theta}_{-\alpha_3}, \mathbf{Y}_1, \dots, \mathbf{Y}_N, \mathbf{X}\right)} \frac{Q\left(\alpha_3|\alpha_3'\right)}{Q\left(\alpha_3'|\alpha_3\right)}\right\}.$$

Let $\mathbf{P}$ be the following tridiagonal matrix:

$$\mathbf{P} = \begin{bmatrix} 1 & -1 & 0 & & \\ -1 & 2 & -1 & & \\ & \ddots & \ddots & \ddots & \\ & & -1 & 2 & -1 \\ & & 0 & -1 & 1 \end{bmatrix}.$$

Thus, letting

$$\mathbf{B}_{\boldsymbol{\nu}_j} = \left(\tau_{\boldsymbol{\nu}_j}\mathbf{P} + \frac{1}{\sigma^2}\sum_{i=1}^{N}\sum_{l=1}^{n_i} Z_{ij}^2 B(t_{il}) B^\intercal(t_{il})\right)^{-1}$$

and

$$\mathbf{b}_{\boldsymbol{\nu}_j} = \frac{1}{\sigma^2}\sum_{i=1}^{N}\sum_{l=1}^{n_i} Z_{ij} B(t_{il}) \left[y_i(t_{il}) - \left(\sum_{k\neq j} Z_{ik}\boldsymbol{\nu}_k^\intercal B(t_{il})\right) - \left(\sum_{k=1}^{K} Z_{ik}\left[\mathbf{x}_i \boldsymbol{\eta}_k^\intercal B(t_{il}) + \sum_{m=1}^{M} \chi_{im} \boldsymbol{\phi}_{kn}^\intercal B(t_{il})\right]\right)\right],$$

we have that

$$\boldsymbol{\nu}_j|\boldsymbol{\Theta}_{-\boldsymbol{\nu}_j}, \mathbf{Y}_1, \dots, \mathbf{Y}_N, \mathbf{X} \sim \mathcal{N}\left(\mathbf{B}_{\boldsymbol{\nu}_j}\mathbf{b}_{\boldsymbol{\nu}_j}, \mathbf{B}_{\boldsymbol{\nu}_j}\right).$$

Let $\boldsymbol{\eta}_{jd}$ denote the dth column of the matrix $\boldsymbol{\eta}_j$. Thus, letting

$$\mathbf{B}_{\boldsymbol{\eta}_{jd}} = \left(\tau_{\boldsymbol{\eta}_{jd}}\mathbf{P} + \frac{1}{\sigma^2}\sum_{i=1}^{N}\sum_{l=1}^{n_i} Z_{ij}^2 x_{id}^2 B(t_{il}) B^\intercal(t_{il})\right)^{-1}$$

and

$$\begin{aligned}\mathbf{b}_{\boldsymbol{\eta}_{jd}} =&\frac{1}{\sigma^2}\sum_{i=1}^{N}\sum_{l=1}^{n_i} Z_{ij}x_{id}B(t_{il})\left[y_i(t_{il}) - \left(\sum_{r\neq d} Z_{ij}x_{ir}\boldsymbol{\eta}_{jr}^\intercal B(t_{il})\right) - \left(\sum_{k\neq j} Z_{ik}\mathbf{x}_i\boldsymbol{\eta}_k^\intercal B(t_{il})\right)\right.\\ &\left. - \left(\sum_{k=1}^{K} Z_{ik}\left[\boldsymbol{\nu}_k^\intercal B(t_{il}) + \sum_{m=1}^{M}\chi_{im}\boldsymbol{\phi}_{kn}^\intercal B(t_{il})\right]\right)\right],\end{aligned}$$

we have that

$$\boldsymbol{\eta}_{jd}|\boldsymbol{\Theta}_{-\boldsymbol{\eta}_{jd}}, \mathbf{Y}_1,\dots,\mathbf{Y}_N,\mathbf{X} \sim \mathcal{N}\left(\mathbf{B}_{\boldsymbol{\eta}_{jd}}\mathbf{b}_{\boldsymbol{\eta}_{jd}}, \mathbf{B}_{\boldsymbol{\eta}_{jd}}\right).$$

Thus we can see that we can draw samples from the posterior of the parameters controlling the mean structure using a Gibbs sampler. Similarly, we can use a Gibbs sampler to draw samples from the posterior distribution of $\tau_{\boldsymbol{\eta}_{jd}}$ and $\tau_{\boldsymbol{\nu}_j}$. We have that the posterior distributions are

$$\tau_{\boldsymbol{\nu}_j}|\boldsymbol{\Theta}_{-\tau_{\boldsymbol{\nu}_j}}, \mathbf{Y}_1,\dots,\mathbf{Y}_N,\mathbf{X} \sim \Gamma\left(\alpha_{\boldsymbol{\nu}} + P/2, \beta_{\boldsymbol{\nu}} + \frac{1}{2}\boldsymbol{\nu}_j^\intercal\mathbf{P}\boldsymbol{\nu}_j\right)$$

and

$$\tau_{\boldsymbol{\eta}_{jd}}|\boldsymbol{\Theta}_{-\tau_{\boldsymbol{\eta}_{jd}}}, \mathbf{Y}_1,\dots,\mathbf{Y}_N,\mathbf{X} \sim \Gamma\left(\alpha_{\boldsymbol{\eta}} + P/2, \beta_{\boldsymbol{\eta}} + \frac{1}{2}\boldsymbol{\eta}_{jd}^\intercal\mathbf{P}\boldsymbol{\eta}_{jd}\right),$$

for $j = 1,\dots,K$ and $d = 1,\dots,R$. The parameter σ^2 can be updated by using a Gibbs update. If we let

$$\beta_\sigma = \frac{1}{2}\sum_{i=1}^{N}\sum_{l=1}^{n_i}\left(y_i(t_{il}) - \sum_{k=1}^{K} Z_{ik}\left((\boldsymbol{\nu}_k + \boldsymbol{\eta}_k\mathbf{x}_i^\intercal)^\intercal B(t_{il}) + \sum_{n=1}^{M}\chi_{in}\boldsymbol{\phi}_{kn}^\intercal B(t_{il})\right)\right)^2,$$

then we have

$$\sigma^2|\boldsymbol{\Theta}_{-\sigma^2}, \mathbf{Y}_1,\dots,\mathbf{Y}_N,\mathbf{X} \sim IG\left(\alpha_0 + \frac{\sum_{i=1}^N n_i}{2}, \beta_0 + \beta_\sigma\right).$$

Lastly, we can update the χ_{im} parameters, for $i = 1,\dots,N$ and $m = 1,\dots,M$, using a Gibbs update. If we let

$$\begin{aligned}\mathbf{w}_{im} =&\frac{1}{\sigma^2}\left[\sum_{l=1}^{n_i}\left(\sum_{k=1}^{K} Z_{ik}\boldsymbol{\phi}_{km}^\intercal B(t_{il})\right)\right.\\ &\left.\left(y_i(t_{il}) - \sum_{k=1}^{K} Z_{ik}\left((\boldsymbol{\nu}_k + \boldsymbol{\eta}_k\mathbf{x}_i^\intercal)^\intercal B(t_{il}) + \sum_{n\neq m}\chi_{in}\boldsymbol{\phi}_{kn}^\intercal B(t_{il})\right)\right)\right]\end{aligned}$$

and

$$\mathbf{W}_{im}^{-1} = 1 + \frac{1}{\sigma^2}\sum_{l=1}^{n_i}\left(\sum_{k=1}^{K} Z_{ik}\boldsymbol{\phi}_{km}^\intercal B(t_{il})\right)^2,$$

then we have that

$$\chi_{im}|\boldsymbol{\zeta}_{-\chi_{im}}, \mathbf{Y}_1,\dots,\mathbf{Y}_N,\mathbf{X} \sim \mathcal{N}(\mathbf{W}_{im}\mathbf{w}_{im}, \mathbf{W}_{im}).$$

2.2 Tempered Transitions

One of the main computational problems we face in these flexible, unsupervised models is a multi-modal posterior distribution. In order to help the Markov chain move across modes, or traverse areas of low posterior probability, we can utilize tempered transitions.

In this paper, we will follow the work of Behrens et al. (2012) and Pritchard et al. (2000) and only temper the likelihood. The target distribution that we want to temper is usually assumed to be written as

$$p(x) \propto \pi(x) exp\left(-\beta_h h(x)\right),$$

where β_h controls how much the distribution is tempered ($1 = \beta_0 < \cdots < \beta_h < \cdots < \beta_{N_t}$). In this setting, we will assume that the hyperparameters N_t and β_{N_t} are user-specified, and will depend on the complexity of the model. For more complex or larger models, we will need to set N_t relatively high. In this implementation, we assume the β_h parameters to follow a geometric scheme, but in more complex models, β_{N_t} may need to be relatively small.

We can rewrite our likelihood for the functional covariate adjusted model to fit the above form:

$$\begin{aligned} p_h(y_i(t)|\boldsymbol{\Theta}, \mathbf{X}) \propto & exp\left\{-\beta_h\left(\frac{1}{2}log(\sigma^2) + \frac{1}{2\sigma^2}\left(y_i(t) - \sum_{k=1}^{K} Z_{ik}\left((\boldsymbol{\nu}_k + \boldsymbol{\eta}_k \mathbf{x}_i^\intercal)^\intercal B(t)\right.\right.\right.\right. \\ & \left.\left.\left.\left. + \sum_{n=1}^{M} \chi_{in}\boldsymbol{\phi}_{k'n}^\intercal B(t)\right)\right)^2\right)\right\} \\ = & \left(\sigma^2\right)^{-\beta_h/2} exp\left\{-\frac{\beta_h}{2\sigma^2}\left(y_i(t) - \sum_{k=1}^{K} Z_{ik}\left((\boldsymbol{\nu}_k + \boldsymbol{\eta}_k \mathbf{x}_i^\intercal)^\intercal B(t)\right.\right.\right. \\ & \left.\left.\left. + \sum_{n=1}^{M} \chi_{in}\boldsymbol{\phi}_{k'n}^\intercal B(t)\right)\right)^2\right\}. \end{aligned}$$

Let $\boldsymbol{\Theta}_h$ be the set of parameters generated from the model using the tempered likelihood associated with β_h. The tempered transition algorithm can be summarized by the following steps:

1. Start with initial state $\boldsymbol{\Theta}_0$.

2. Transition from $\boldsymbol{\Theta}_0$ to $\boldsymbol{\Theta}_1$ using the tempered likelihood associated with β_1.

3. Continue in this manner until we transition from $\mathbf{\Theta}_{N_t-1}$ to $\mathbf{\Theta}_{N_t}$ using the tempered likelihood associated with β_{N_t}.

4. Transition from $\mathbf{\Theta}_{N_t}$ to $\mathbf{\Theta}_{N_t+1}$ using the tempered likelihood associated with β_{N_t}.

5. Continue in this manner until we transition from $\mathbf{\Theta}_{2N_t-1}$ to $\mathbf{\Theta}_{2N_t}$ using β_1.

6. Accept transition from $\mathbf{\Theta}_0$ to $\mathbf{\Theta}_{2N_t}$ with probability

$$\min\left\{1, \prod_{h=0}^{N_t-1} \frac{\prod_{i=1}^N \prod_{l=1}^{n_i} p_{h+1}(y_i(t_{il})|\mathbf{\Theta}_h, \mathbf{X}_i)}{\prod_{i=1}^N \prod_{l=1}^{n_i} p_h(y_i(t_{il})|\mathbf{\Theta}_h, \mathbf{X}_i)} \prod_{h=N_t+1}^{2N_t} \frac{\prod_{i=1}^N \prod_{l=1}^{n_i} p_h(y_i(t_{il})|\mathbf{\Theta}_h, \mathbf{X}_i)}{\prod_{i=1}^N \prod_{l=1}^{n_i} p_{h+1}(y_i(t_{il})|\mathbf{\Theta}_h, \mathbf{X}_i)}\right\}$$

in the functional case, or

$$\min\left\{1, \prod_{h=0}^{N_t-1} \frac{\prod_{i=1}^N \prod_{l=1}^{n_i} p_{h+1}(\mathbf{y}_i|\mathbf{\Theta}_h, \mathbf{X}_i)}{\prod_{i=1}^N \prod_{l=1}^{n_i} p_h(\mathbf{y}_i)|\mathbf{\Theta}_h, \mathbf{X}_i)} \prod_{h=N_t+1}^{2N_t} \frac{\prod_{i=1}^N \prod_{l=1}^{n_i} p_h(\mathbf{y}_i|\mathbf{\Theta}_h, \mathbf{X}_i)}{\prod_{i=1}^N \prod_{l=1}^{n_i} p_{h+1}(\mathbf{y}_i|\mathbf{\Theta}_h, \mathbf{X}_i)}\right\}$$

in the multivariate case.

Since we only temper the likelihood, many of the posterior distributions derived in section 2.1 can be utilized. Thus the following posteriors are the only ones that change due to the tempering of the likelihood. Starting with the $\mathbf{\Phi}$ parameters, we have

$$\begin{aligned}\left(\mathbf{m}_{\phi_{jm}}\right)_h =& \frac{\beta_h}{(\sigma^2)_h} \sum_{i=1}^N \sum_{l=1}^{n_i} \Bigg(B(t_{il})(\chi_{im})_h \Big(y_i(t_{il})(Z_{ij})_h - (Z_{ij})_h^2 \left((\boldsymbol{\nu}_j)_h + (\boldsymbol{\eta}_j)_h \mathbf{x}_i^\intercal\right)^\intercal B(t_{il}) \\ & -(Z_{ij})_h^2 \sum_{n \neq m} (\chi_{in})_h (\boldsymbol{\phi}_{jn})_h^\intercal B(t_{il}) \\ & - \sum_{k \neq j} Z_{ij} Z_{ik} \left[\left((\boldsymbol{\nu}_k)_h + (\boldsymbol{\eta}_k)_h \mathbf{x}_i^\intercal\right)^\intercal B(t_{il}) + \sum_{n=1}^M \chi_{in} (\boldsymbol{\phi}_{kn})_h^\intercal B(t_{il})\right]\Big)\Bigg),\end{aligned}$$

and

$$\left(\mathbf{M}_{\phi_{jm}}\right)_h^{-1} = \frac{\beta_h}{(\sigma^2)_h} \sum_{i=1}^N \sum_{l=1}^{n_i} \left((Z_{ij})_h^2 (\chi_{im})_h^2 B(t_{il}) B^\intercal(t_{il})\right) + \left(\mathbf{D}_{\phi_{jm}}\right)_h^{-1},$$

we have that

$$\left(\boldsymbol{\phi}_{jm}\right)_h | \mathbf{\Theta}_{-\left(\phi_{jm}\right)_h}, \mathbf{Y}_1, \dots, \mathbf{Y}_N, \mathbf{X} \sim \mathcal{N}\left(\left(\mathbf{M}_{\phi_{jm}}\right)_h \left(\mathbf{m}_{\phi_{jm}}\right)_h, \left(\mathbf{M}_{\phi_{jm}}\right)_h\right).$$

As in the untempered case, we have that the posterior distribution $\mathbf{Z}$ parameters under the tempered likelihood are not a commonly known distributions. Therefore, we will use the Metropolis-Hastings

algorithm. We have that

$$
\begin{aligned}
p((\mathbf{z}_i)_h|\boldsymbol{\Theta}_{-(\mathbf{z}_i)_h}, \mathbf{Y}_1, \dots, \mathbf{Y}_N, \mathbf{X}) \propto & \prod_{k=1}^{K} (Z_{ik})_h^{(\alpha_3)_h(\pi_k)_h - 1} \\
& \times \prod_{l=1}^{n_i} exp\left\{ -\frac{\beta_h}{2(\sigma^2)_h} \left(y_i(t_{il}) - \sum_{k=1}^{K} (Z_{ik})_h \left(((\boldsymbol{\nu}_k)_h + (\boldsymbol{\eta}_k)_h \mathbf{x}_i^\intercal)^\intercal B(t_{il}) \right.\right.\right. \\
& \left.\left.\left. + \sum_{m=1}^{M} (\chi_{im})_h (\boldsymbol{\phi}_{km})_h^\intercal B(t_{il}) \right) \right)^2 \right\}.
\end{aligned}
$$

We will use $Q((\mathbf{z}_i)_h'|(\mathbf{z}_i)_h) = Dir(a_{\mathbf{z}}(\mathbf{z}_i)_h)$ for some large $a_{\mathbf{z}} \in \mathbb{R}^+$ as the proposal distribution. Thus the probability of accepting a proposed step is

$$
A((\mathbf{z}_i)_h', (\mathbf{z}_i)_h) = \min\left\{ 1, \frac{P\left((\mathbf{z}_i)_h'|\boldsymbol{\Theta}_{-(\mathbf{z}_i)_h'}, \mathbf{Y}_1, \dots, \mathbf{Y}_N, \mathbf{X}\right)}{P\left((\mathbf{z}_i)_h|\boldsymbol{\Theta}_{-(\mathbf{z}_i)_h}, \mathbf{Y}_1, \dots, \mathbf{Y}_N, \mathbf{X}\right)} \frac{Q\left((\mathbf{z}_i)_h|(\mathbf{z}_i)_h'\right)}{Q\left((\mathbf{z}_i)_h'|(\mathbf{z}_i)_h\right)} \right\}.
$$

Letting

$$
\left(\mathbf{B}_{\boldsymbol{\nu}_j}\right)_h = \left(\left(\tau_{\boldsymbol{\nu}_j}\right)_h \mathbf{P} + \frac{\beta_h}{(\sigma^2)_h} \sum_{i=1}^{N} \sum_{l=1}^{n_i} (Z_{ij})_h^2 B(t_{il}) B^\intercal(t_{il}) \right)^{-1}
$$

and

$$
\begin{aligned}
\left(\mathbf{b}_{\boldsymbol{\nu}_j}\right)_h = & \frac{\beta_h}{(\sigma^2)_h} \sum_{i=1}^{N} \sum_{l=1}^{n_i} (Z_{ij})_h B(t_{il}) \left[y_i(t_{il}) - \left(\sum_{k \neq j} (Z_{ik})_h (\boldsymbol{\nu}_k^\intercal)_h B(t_{il}) \right) \right. \\
& \left. - \left(\sum_{k=1}^{K} (Z_{ik}) \left[\mathbf{x}_i (\boldsymbol{\eta}_k)_h^\intercal B(t_{il}) + \sum_{m=1}^{M} (\chi_{im})_h (\boldsymbol{\phi}_{kn})_h^\intercal B(t_{il}) \right] \right) \right],
\end{aligned}
$$

we have that

$$
(\boldsymbol{\nu}_j)_h \, | \boldsymbol{\Theta}_{-(\boldsymbol{\nu}_j)_h}, \mathbf{Y}_1, \dots, \mathbf{Y}_N, \mathbf{X} \sim \mathcal{N}\left(\left(\mathbf{B}_{\boldsymbol{\nu}_j}\right)_h \left(\mathbf{b}_{\boldsymbol{\nu}_j}\right)_h, \left(\mathbf{B}_{\boldsymbol{\nu}_j}\right)_h \right).
$$

Let $\left(\boldsymbol{\eta}_{jd}\right)_h$ denote the dth column of the matrix $(\boldsymbol{\eta}_j)_h$. Thus, letting

$$
\left(\mathbf{B}_{\boldsymbol{\eta}_{jd}}\right)_h = \left(\left(\tau_{\boldsymbol{\eta}_{jd}}\right)_h \mathbf{P} + \frac{\beta_h}{(\sigma^2)_h} \sum_{i=1}^{N} \sum_{l=1}^{n_i} (Z_{ij})_h^2 x_{id}^2 B(t_{il}) B^\intercal(t_{il}) \right)^{-1}
$$

and

$$
\begin{aligned}
\left(\mathbf{b}_{\boldsymbol{\eta}_{jd}}\right)_h = & \frac{\beta_h}{(\sigma^2)_h} \sum_{i=1}^{N} \sum_{l=1}^{n_i} (Z_{ij})_h x_{id} B(t_{il}) \left[y_i(t_{il}) - \left(\sum_{r \neq d} (Z_{ij})_h x_{ir} (\boldsymbol{\eta}_{jr})_h^\intercal B(t_{il}) \right) \right. \\
& - \left(\sum_{k \neq j} (Z_{ik})_h \mathbf{x}_i (\boldsymbol{\eta}_k)_h^\intercal B(t_{il}) \right) \\
& \left. - \left(\sum_{k=1}^{K} (Z_{ik})_h \left[(\boldsymbol{\nu}_k)_h^\intercal B(t_{il}) + \sum_{m=1}^{M} (\chi_{im})_h (\boldsymbol{\phi}_{kn})_h^\intercal B(t_{il}) \right] \right) \right],
\end{aligned}
$$

we have that

$$\left(\boldsymbol{\eta}_{jd}\right)_h | \boldsymbol{\Theta}_{-\left(\boldsymbol{\eta}_{jd}\right)_h}, \mathbf{Y}_1, \dots, \mathbf{Y}_N, \mathbf{X} \sim \mathcal{N}\left(\left(\mathbf{B}_{\boldsymbol{\eta}_{jd}}\right)_h \left(\mathbf{b}_{\boldsymbol{\eta}_{jd}}\right)_h, \left(\mathbf{B}_{\boldsymbol{\eta}_{jd}}\right)_h\right).$$

If we let

$$\left(\beta_\sigma\right)_h = \frac{\beta_h}{2}\sum_{i=1}^N\sum_{l=1}^{n_i}\left(y_i(t_{il}) - \sum_{k=1}^K (Z_{ik})_h\left(\left((\boldsymbol{\nu}_k)_h + (\boldsymbol{\eta}_k)_h\mathbf{x}_i^\intercal\right)^\intercal B(t_{il}) + \sum_{n=1}^M (\chi_{in})_h(\boldsymbol{\phi}_{kn})_h^\intercal B(t_{il})\right)\right)^2,$$

then we have

$$(\sigma^2)_h | \boldsymbol{\Theta}_{-(\sigma^2)_h}, \mathbf{Y}_1, \dots, \mathbf{Y}_N, \mathbf{X} \sim IG\left(\alpha_0 + \frac{\beta_h \sum_{i=1}^N n_i}{2}, \beta_0 + (\beta_\sigma)_h\right).$$

Lastly, we can update the χ_{im} parameters, for $i = 1, \dots, N$ and $m = 1, \dots, M$, using a Gibbs update. If we let

$$(\mathbf{w}_{im})_h = \frac{\beta_h}{(\sigma^2)_h}\left[\sum_{l=1}^{n_i}\left(\sum_{k=1}^K (Z_{ik})_h(\boldsymbol{\phi}_{km})_h^\intercal B(t_{il})\right)\left(y_i(t_{il}) - \sum_{k=1}^K (Z_{ik})_h\left(\left((\boldsymbol{\nu}_k)_h + (\boldsymbol{\eta}_k)_h\mathbf{x}_i^\intercal\right)^\intercal B(t_{il}) + \sum_{n\neq m}(\chi_{in})_h(\boldsymbol{\phi}_{kn})_h^\intercal B(t_{il})\right)\right)\right]$$

and

$$(\mathbf{W}_{im})_h^{-1} = 1 + \frac{\beta_h}{\sigma^2}\sum_{l=1}^{n_i}\left(\sum_{k=1}^K (Z_{ik})_h(\boldsymbol{\phi}_{km})_h^\intercal B(t_{il})\right)^2,$$

then we have that

$$(\chi_{im})_h | \boldsymbol{\zeta}_{-(\chi_{im})_h}, \mathbf{Y}_1, \dots, \mathbf{Y}_N, \mathbf{X} \sim \mathcal{N}\left((\mathbf{W}_{im})_h(\mathbf{w}_{im})_h, (\mathbf{W}_{im})_h\right).$$

3 Simulation Study and Case Studies

3.1 Simulation Study 1

This subsection contains detailed information on how the simulation study in Section 3 of the main text was conducted. This simulation study primarily looked at how well we could recover the true mean structure, the covariance structure, and the allocation structure. In this simulation

study, we simulated datasets from 3 scenarios at 3 different sample sizes for each scenario. Once the datasets were generated, we fit a variety of covariate adjusted functional mixed membership models, as well as unadjusted functional mixed membership models, on the datasets to see how well we could recover the mean, covariance, and allocation structures.

The first scenario we considered was a covariate adjusted functional mixed membership model with 2 true covariates. To generate all of the datasets, we assumed that the observations were in the span of B-spline basis with 8 basis functions. For this scenario, we generated 3 datasets with sample sizes of 60, 120, and 240 functional observations, all observed on a grid of 25 time points. The data were generated by first generating the model parameters (as discussed below) and then generating data from the likelihood specified in Equation 8 of the main text. The model parameters for this dataset were generated as follows:

$$\boldsymbol{\nu}_1 \sim \mathcal{N}\left((6, 4, \dots, -6, -8)^\intercal, 4\mathbf{P}\right),$$

$$\boldsymbol{\nu}_2 \sim \mathcal{N}\left((-8, -6, \dots, 4, 6)^\intercal, 4\mathbf{P}\right),$$

$$\boldsymbol{\eta}_{k1} \sim \mathcal{N}\left(\mathbf{1}, \mathbf{P}\right) \quad k = 1, 2,$$

$$\boldsymbol{\eta}_{k2} \sim \mathcal{N}\left((3, 2, \dots, -4)^\intercal, \mathbf{P}\right) \quad k = 1, 2.$$

The $\boldsymbol{\Phi}$ parameters were drawn according to the following distributions:

$$\boldsymbol{\phi}_{km} = \mathbf{q}_{km} \quad k = 1, 2 \quad m = 1, 2, 3,$$

where $\mathbf{q}_{k1} \sim \mathcal{N}(\mathbf{0}_8, 2.25\mathbf{I}_8)$, $\mathbf{q}_{k2} \sim \mathcal{N}(\mathbf{0}_8, \mathbf{I}_8)$, $\mathbf{q}_{k3} \sim \mathcal{N}(\mathbf{0}_8, 0.49\mathbf{I}_8)$. The χ_{im} parameters were drawn from a standard normal distribution. The $\mathbf{z}_i$ parameters were drawn from a mixture of Dirichlet distributions. Roughly 30% of the $\mathbf{z}_i$ parameters were drawn from a Dirichlet distribution with $\alpha_1 = 10$ and $\alpha_2 = 1$. Another roughly 30% of the $\mathbf{z}_i$ parameters were drawn from a Dirichlet distribution where $\alpha_1 = 1$ and $\alpha_2 = 10$. The rest of the $\mathbf{z}_i$ parameters were drawn from a Dirichlet distribution with $\alpha_1 = \alpha_2 = 1$. The covariates, $\mathbf{X}$, were drawn from a standard normal distribution. The models in this scenario were run for 500,000 MCMC iterations.

For the second scenario, we considered data drawn from a covariate adjusted functional mixed membership model with one covariate. We considered three sample sizes of 50, 100, and 200 functional samples observed on a grid of 25 time points. The model parameters for this dataset were generated as follows:

$$\boldsymbol{\nu}_1 \sim \mathcal{N}\left((6, 4, \dots, -6, -8)^\intercal, 4\mathbf{P}\right),$$

$$\boldsymbol{\nu}_2 \sim \mathcal{N}\left((-8, -6, \dots, 4, 6)^\intercal, 4\mathbf{P}\right),$$

$$\boldsymbol{\eta}_{11} \sim \mathcal{N}\left(\mathbf{2}, \mathbf{P}\right),$$

$$\boldsymbol{\eta}_{21} \sim \mathcal{N}\left(-\mathbf{2}, \mathbf{P}\right).$$

The $\boldsymbol{\Phi}$ parameters were drawn according to the following distributions:

$$\boldsymbol{\phi}_{km} = \mathbf{q}_{km} \quad k = 1, 2 \quad m = 1, 2, 3,$$

where $\mathbf{q}_{k1} \sim \mathcal{N}(\mathbf{0}_8, 2.25\mathbf{I}_8)$, $\mathbf{q}_{k2} \sim \mathcal{N}(\mathbf{0}_8, \mathbf{I}_8)$, $\mathbf{q}_{k3} \sim \mathcal{N}(\mathbf{0}_8, 0.49\mathbf{I}_8)$. The χ_{im} parameters were drawn from a standard normal distribution. The $\mathbf{z}_i$ parameters were drawn from a mixture of Dirichlet distributions. Roughly 30% of the $\mathbf{z}_i$ parameters were drawn from a Dirichlet distribution with $\alpha_1 = 10$ and $\alpha_2 = 1$. Another roughly 30% of the $\mathbf{z}_i$ parameters were drawn from a Dirichlet distribution where $\alpha_1 = 1$ and $\alpha_2 = 10$. The rest of the $\mathbf{z}_i$ parameters were drawn from a Dirichlet distribution with $\alpha_1 = \alpha_2 = 1$. The covariates, $\mathbf{X}$, were drawn from a normal distribution with variance of nine and mean of zero. The models in this scenario were run for 500,000 MCMC iterations.

When calculating the R-MISE for the under-specified model (i.e. the model with no covariates), we use the marginal mean structure and the marginal covariance structures as the generating truth. In this case, since the covariates were drawn from a standard normal distribution, we have that the marginal mean structures will take the form $\mu^{(k)}(t) = \boldsymbol{\nu}_k^\intercal \mathbf{B}(t)$, and the marginal covariance structure will take the form $C^{(k,k')}(s,t) = \mathbf{B}^\intercal(s)\left(\sum_{m=1}^{KP} \boldsymbol{\phi}_{km}\boldsymbol{\phi}_{k'm}^\intercal\right)\mathbf{B}(t)$ for $k \neq k'$ and $C^{(k,k)}(s,t) = \mathbf{B}^\intercal(s)\left(\boldsymbol{\eta}_{k1}\boldsymbol{\eta}_{k1}^\intercal + \sum_{m=1}^{KP} \boldsymbol{\phi}_{km}\boldsymbol{\phi}_{km}^\intercal\right)\mathbf{B}(t)$ for $k = 1, 2$.

For the third scenario, we generated data from an unadjusted functional mixed membership model. We considered three sample sizes of 40, 80, and 160 functional samples observed on a grid of 25 time points. The model parameters for this dataset were generated as follows:

$$\boldsymbol{\nu}_1 \sim \mathcal{N}\left((6, 4, \dots, -6, -8)^\intercal, 4\mathbf{P}\right),$$

$$\boldsymbol{\nu}_2 \sim \mathcal{N}\left((-8, -6, \dots, 4, 6)^\intercal, 4\mathbf{P}\right),$$

The $\boldsymbol{\Phi}$ parameters were drawn according to the following distributions:

$$\boldsymbol{\phi}_{km} = \mathbf{q}_{km} \quad k = 1, 2 \quad m = 1, 2,$$

where $\mathbf{q}_{k1} \sim \mathcal{N}(\mathbf{0}_8, 2.25\mathbf{I}_8)$, $\mathbf{q}_{k2} \sim \mathcal{N}(\mathbf{0}_8, \mathbf{I}_8)$, $\mathbf{q}_{k3} \sim \mathcal{N}(\mathbf{0}_8, 0.49\mathbf{I}_8)$. The χ_{im} parameters were drawn from a standard normal distribution. The $\mathbf{z}_i$ parameters were drawn from a mixture of Dirichlet distributions. Approximately 30% of the $\mathbf{z}_i$ parameters were drawn from a Dirichlet distribution with $\alpha_1 = 10$ and $\alpha_2 = 1$. Another roughly 30% of the $\mathbf{z}_i$ parameters were drawn from a Dirichlet distribution where $\alpha_1 = 1$ and $\alpha_2 = 10$. The rest of the $\mathbf{z}_i$ parameters were drawn from a Dirichlet distribution with $\alpha_1 = \alpha_2 = 1$. The models in this scenario were run for 500,000 MCMC iterations. The code for running this simulation study can be found on Github.

The following plots are more detailed visualizations of the results obtained in the first simulation study.

3.2 Simulation Study 2

In this simulation study, we evaluated the performance of AIC, BIC, DIC, and the elbow method in choosing the number of features in a covariate adjusted mixed membership model. In this simulation study, we considered a covariate adjusted functional mixed membership model with only one continuous covariate. We generated 50 different data sets with 150 functional observations, observed along a uniform grid of 25 time points. The data was generated from a covariate adjusted mixed membership model with model parameters generated as follows:

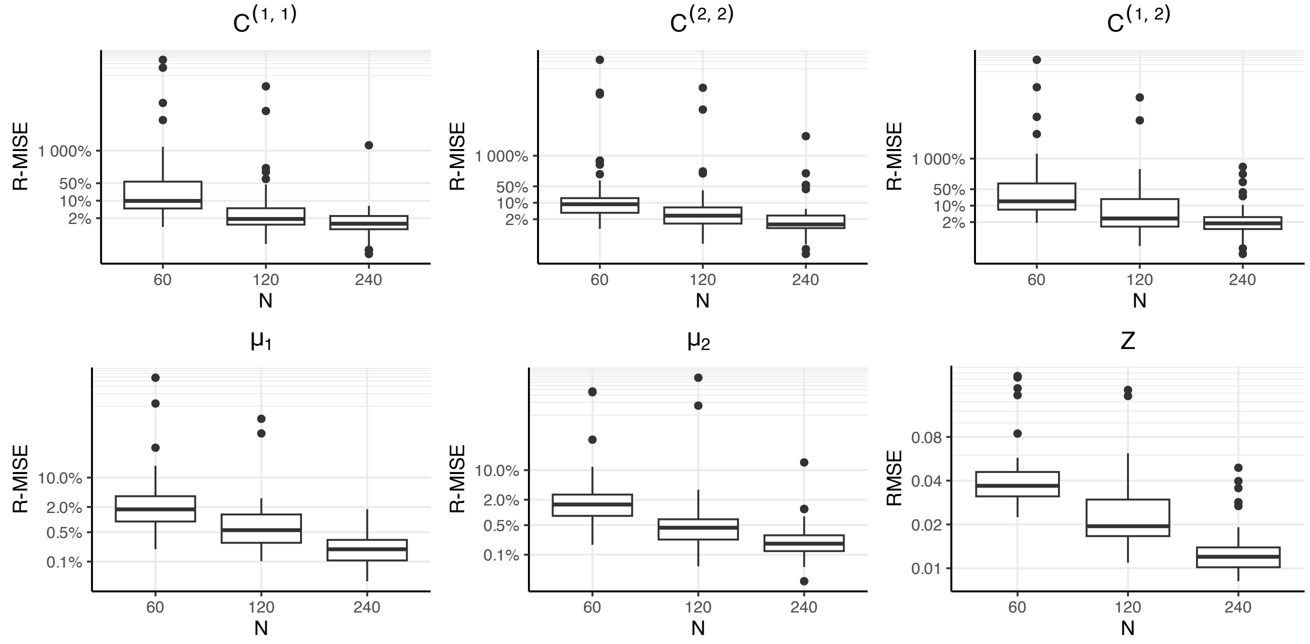

Figure 1: RMISE and RMSE results from the simulations models fit with two covariates, where the true data was generated with two covariates.

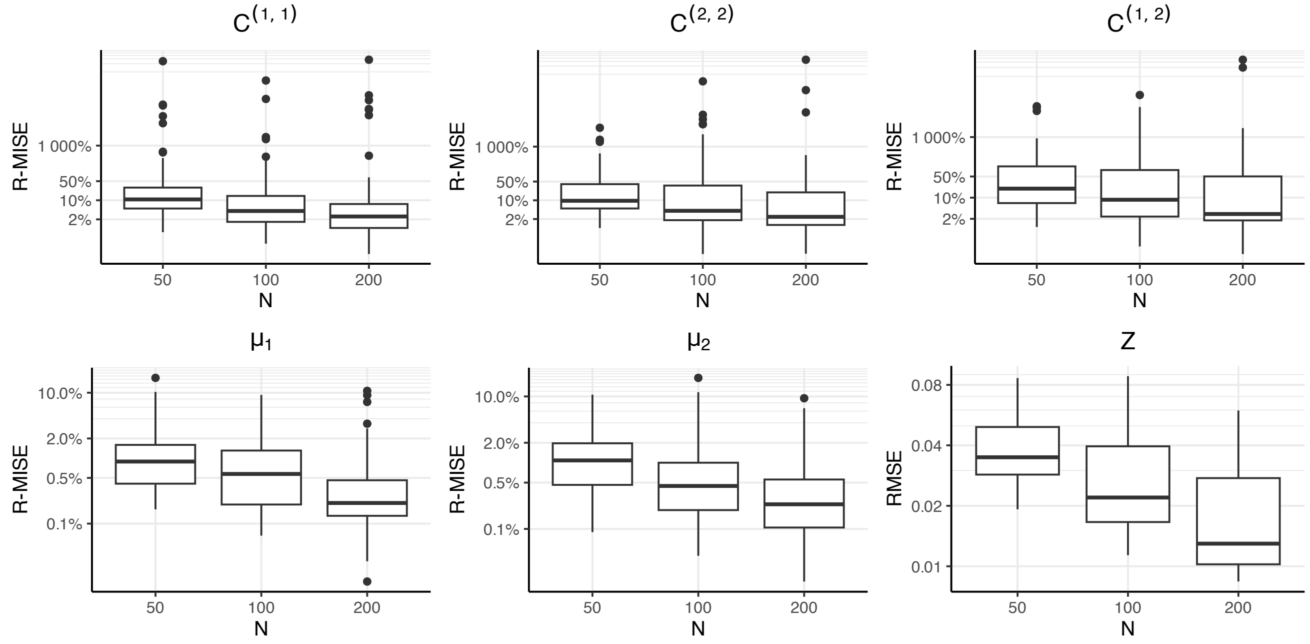

Figure 2: RMISE and RMSE results from the simulations models fit with one covariate, where the true data was generated with one covariate.

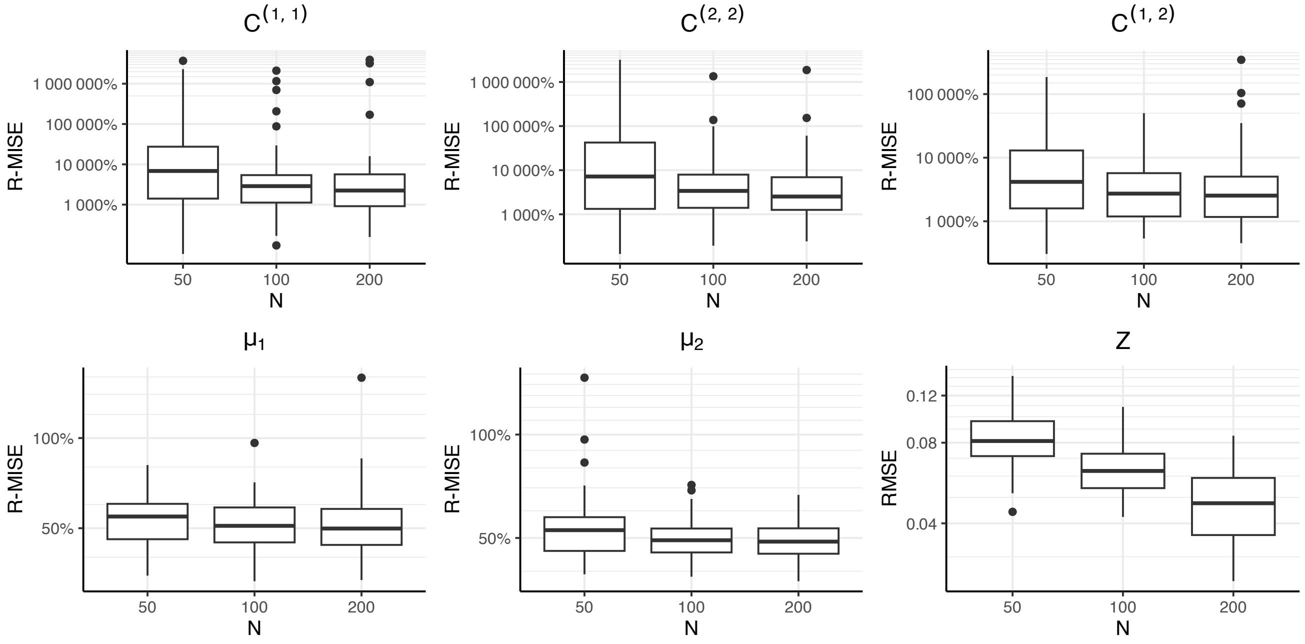

Figure 3: RMISE and RMSE results from the simulations models fit with no covariates, where the true data was generated with one covariate.

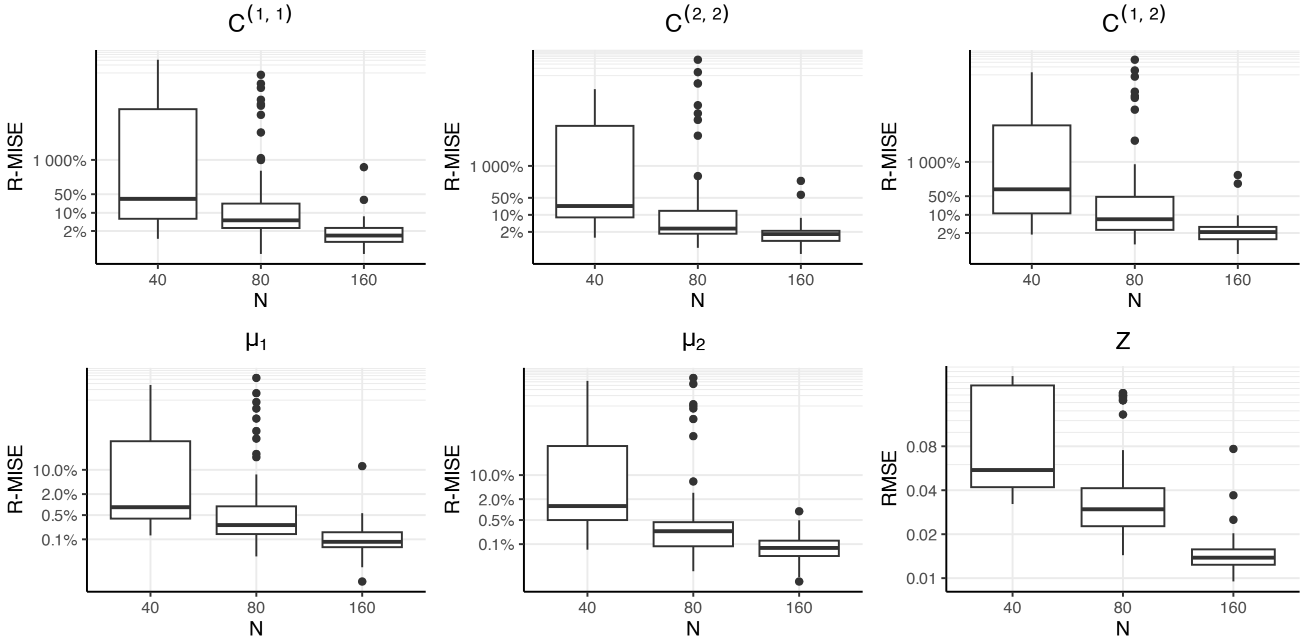

Figure 4: RMISE and RMSE results from the simulations models fit with one covariate, where the true data was generated with no covariates.

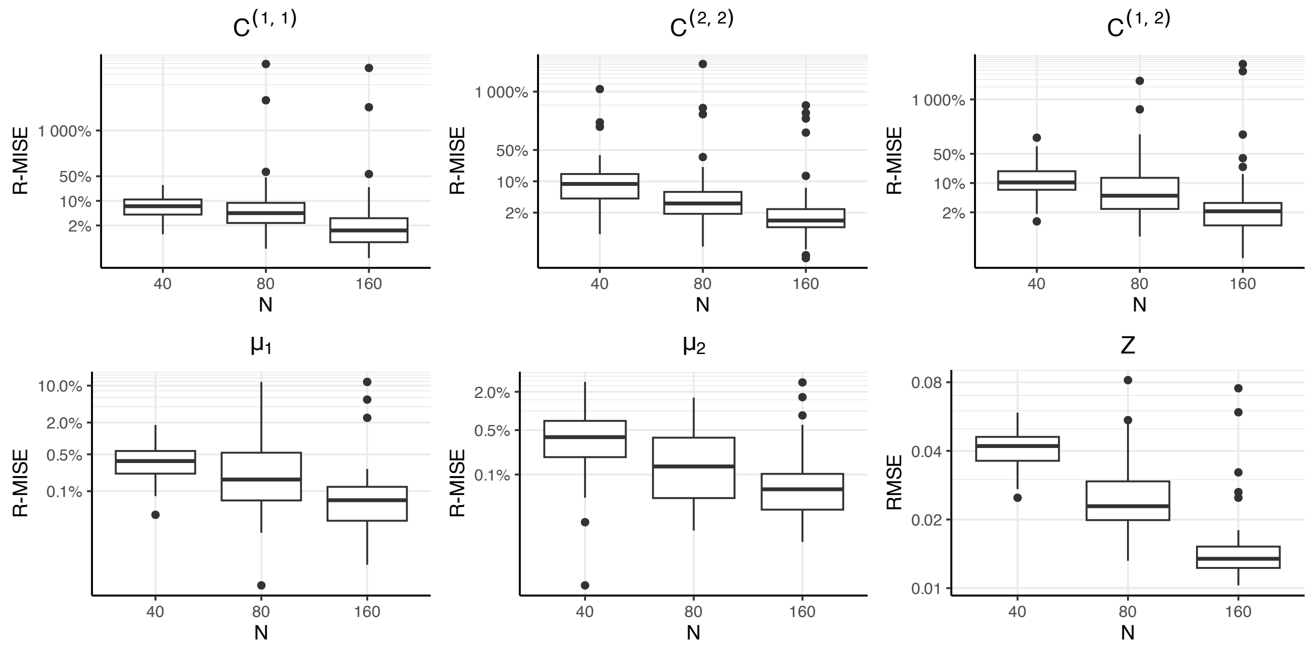

Figure 5: RMISE and RMSE results from the simulations models fit with no covariates, where the true data was generated with no covariates.

$$\boldsymbol{\nu}_1 \sim \mathcal{N}\left((6, 4, \ldots, -6, -8)^\intercal, 4\mathbf{P}\right),$$

$$\boldsymbol{\nu}_2 \sim \mathcal{N}\left((-8, -6, \ldots, 4, 6)^\intercal, 4\mathbf{P}\right),$$

$$\boldsymbol{\eta}_{11} \sim \mathcal{N}\left(\mathbf{2}, \mathbf{P}\right),$$

$$\boldsymbol{\eta}_{21} \sim \mathcal{N}\left(-\mathbf{2}, \mathbf{P}\right),$$

$$\boldsymbol{\eta}_{21} \sim \mathcal{N}\left(\mathbf{1}, \mathbf{P}\right).$$

The $\mathbf{\Phi}$ parameters were drawn according to the following distributions:

$$\boldsymbol{\phi}_{km} = \mathbf{q}_{km} \quad k = 1, 2 \quad m = 1, 2,$$

where $\mathbf{q}_{k1} \sim \mathcal{N}(\mathbf{0}_8, 2.25\mathbf{I}_8)$, $\mathbf{q}_{k2} \sim \mathcal{N}(\mathbf{0}_8, \mathbf{I}_8)$, $\mathbf{q}_{k3} \sim \mathcal{N}(\mathbf{0}_8, 0.49\mathbf{I}_8)$. The χ_{im} parameters were drawn from a standard normal distribution. The $\mathbf{z}_i$ parameters were drawn from a mixture of Dirichlet distributions. Approximately 20% of the $\mathbf{z}_i$ parameters were drawn from a Dirichlet distribution with $\alpha_1 = 30$, $\alpha_2 = 1$, and $\alpha_3 = 1$. Another roughly 20% of the $\mathbf{z}_i$ parameters were drawn from

a Dirichlet distribution where $\alpha_1 = 1$, $\alpha_2 = 30$, and $\alpha_3 = 1$. Another roughly 20% of the $\mathbf{z}_i$ parameters were drawn from a Dirichlet distribution where $\alpha_1 = 1$, $\alpha_2 = 1$, and $\alpha_3 = 30$. The rest of the $\mathbf{z}_i$ parameters were drawn from a Dirichlet distribution with $\alpha_1 = \alpha_2 = \alpha_3 = 1$. Four models were then fit for each dataset, with $K = 2, 3, 4, 5$. The models in this scenario were run for 200,000 MCMC iterations.

The Bayesian Information Criterion (BIC), proposed by Schwarz (1978), is defined as:

$$\text{BIC} = 2\text{log}P\left(\mathbf{Y}|\hat{\boldsymbol{\Theta}}, \mathbf{X}\right) - d\text{log}(n)$$

where d is the number of parameters and $\hat{\boldsymbol{\Theta}}$ are the maximum likelihood estimators (MLE) of our parameters. In the case of our proposed model, we have that

$$\text{BIC} = 2\text{log}P\left(\mathbf{Y}|\hat{\boldsymbol{\nu}}_{1:K}, \hat{\boldsymbol{\eta}}_{1:K}, \hat{\boldsymbol{\Phi}}_{1:KM}, \hat{\sigma}^2, \hat{\mathbf{Z}}, \hat{\boldsymbol{\chi}}, \mathbf{X}\right) - d\text{log}(\tilde{N}) \tag{5}$$

where $\tilde{N} = \sum_i n_i$ (where n_i is the number of observed time points observed for the ith function), and $d = (N + P)K + 2MKP + 4K + (N + K)M + 2 + PRK + KR$.

Similarly, the AIC, proposed by Akaike (1974), can be written as

$$\text{AIC} = -2\text{log}P\left(\mathbf{Y}|\hat{\boldsymbol{\nu}}_{1:K}, \hat{\boldsymbol{\eta}}_{1:K}, \hat{\boldsymbol{\Phi}}_{1:KM}, \hat{\sigma}^2, \hat{\mathbf{Z}}, \hat{\boldsymbol{\chi}}, \mathbf{X}\right) + 2d. \tag{6}$$

Following the work of Roeder & Wasserman (1997), we will use the posterior mean instead of the MLE for our estimates of BIC and AIC.

The modified DIC, proposed by Celeux et al. (2006), is advantageous to the original DIC proposed by Spiegelhalter et al. (2002) when we have a posterior distribution with multiple modes and when identifiability may be a problem. The modified DIC (referred to as DIC_3 in Celeux et al. (2006)) is specified as

$$\text{DIC} = -4\mathbb{E}_{\boldsymbol{\Theta}}[\text{log}f(\mathbf{Y}|\boldsymbol{\Theta}), \mathbf{X}|\mathbf{Y}] + 2log\hat{f}(\mathbf{Y}) \tag{7}$$

where $\hat{f}(y_{ij}) = \frac{1}{N_{MC}}\sum_{l=1}^{N_{MC}} P\left(y_{ij}|\boldsymbol{\nu}_{1:K}^{(l)}, \boldsymbol{\eta}_{1:K}^{(l)}, \boldsymbol{\Phi}_{1:KM}^{(l)}, (\sigma^2)^{(l)}, \mathbf{Z}^{(l)}, \mathbf{x}_i\right)$, $\hat{f}(\mathbf{Y}) = \prod_{i=1}^{N}\prod_{j=1}^{n_i}\hat{f}(y_{ij})$, and N_{MC} is the number of MCMC samples used for estimating $\hat{f}(y_{ij})$. We can approximate

$\mathbb{E}_{\boldsymbol{\Theta}}[\log f(\mathbf{Y}|\boldsymbol{\Theta})|\mathbf{Y}]$ by using the MCMC samples, such that

$$\mathbb{E}_{\boldsymbol{\Theta}}[\log f(\mathbf{Y}|\boldsymbol{\Theta},\mathbf{X})|\mathbf{Y}] \approx \frac{1}{N_{MC}}\sum_{l=1}^{N_{MC}}\sum_{i=1}^{N}\sum_{j=1}^{n_i}\log\left[P\left(y_{ij}|\boldsymbol{\nu}_{1:K}^{(l)},\boldsymbol{\eta}_{1:K}^{(l)},\boldsymbol{\Phi}_{1:KM}^{(l)},\left(\sigma^2\right)^{(l)},\mathbf{Z}^{(l)},\mathbf{x}_i\right)\right].$$

3.3 Case Study

As discussed in the main manuscript, we fit two covariate adjusted functional mixed membership models using resting-state EEG data from Dickinson et al. (2018). The first model used only the log transformation of age as the covariate, and the second model used the log transformation of age, diagnostic group (ASD vs TD), and an interaction between the log transformation of age and diagnostic group as the covariates.

While the gender of the child and clinical measures, such as verbal IQ (VIQ) and Non-verbal IQ (NVIQ), were not formally included in the analysis, here we provide visualizations of the correlation between these variables and the allocation parameter estimates in the age-adjusted functional mixed membership model in Section 4.1 of the main manuscript (Figure 6). Crucially, we can see that individuals that were estimated to load higher on feature 2 were more likely to have a higher VIQ and NVIQ. However, we can see that there was no noticeable difference between the females and males in the study. We note that due to the limited sample size, we were unable to formally include these variables in an analysis.

While the results from the covariate adjusted functional mixed membership model were insightful and provided novel insight into the subject-level heterogeneity of developmental trajectories, one may be interested if similar insight could be gained from a simpler model, such as the finite mixture of regressions model. Thus, we used the MoEClust package Murphy & Murphy (2020) to fit a mixture of expert (or finite mixture of regressions) model. We note that this package is for general multivariate data and does not make any of the common assumptions used in functional data analysis (such as smoothness). The results of the model can be seen in Figure 7. We can see that cluster 1 represents individuals with a distinct alpha peak (periodic signal) and cluster 2 represents individuals with a $1/f$ signal (aperiodic signal). While cluster 1 captures the more attenuated peak

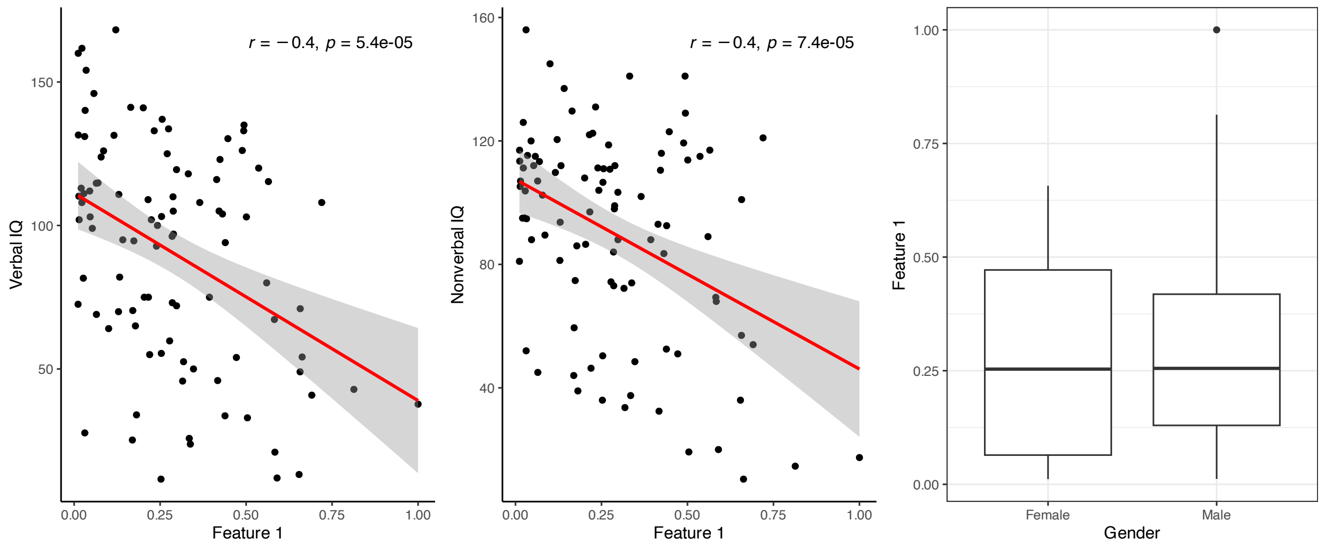

Figure 6: Association between allocation parameters estimated in the age-adjusted functional mixed membership model and gender, VIQ, and NVIQ.

that develops as children age, the model fails to capture the shift in PAF to higher frequencies, which was found in previous studies and in our analysis. Furthermore, what we obtain from these models are the probabilities of inclusion in one of the two clusters. This is conceptually different from *partial* or *mixed* membership, as we can no longer view individuals on a spectrum but instead from coming from one of the two clusters. This also contradicts the scientific literature, which posits that the EEG spectra contain a *continuous* mixture of aperiodic and periodic signal.

The group-specific mean structures for each of the two features found in Section 4.2 of the main manuscript can be visualized in Figure 8. As stated in the manuscript, these feature means cannot be interpreted as the expected trajectories of the most extreme observations and are thus harder to directly interpret.

3.4 Comparison Between Mixed Membership Models

In the main manuscript, we extended the analysis of alpha oscillations conducted by Marco et al. (2024) to allow for a covariate-dependent mixed membership model. While previous studies (Haegens et al. 2014, Rodríguez-Martínez et al. 2017, Scheffler et al. 2019) have shown that alpha

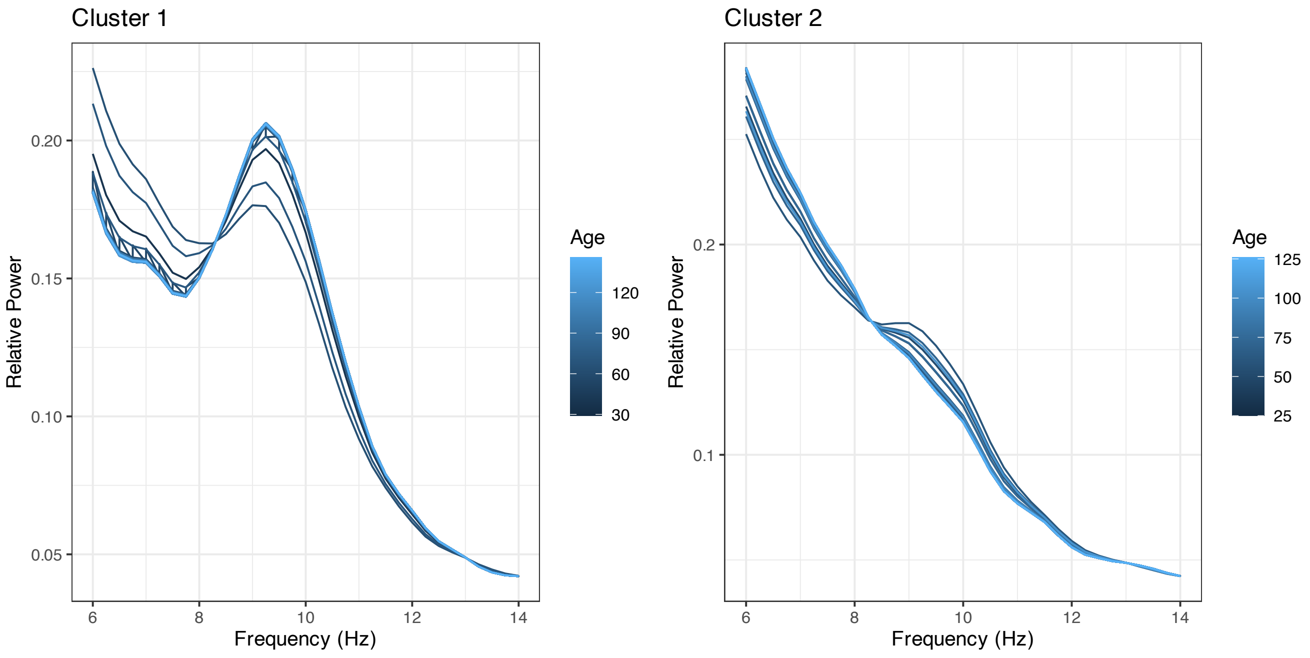

Figure 7: Results from a finite mixture of regressions model, with the logarithm of age as the covariate.

oscillations depend on age, little is known about how alpha oscillations differ between children with ASD and TD children conditional on age. Although it is attractive to add more covariates to our model, the small sample sizes often found in neurodevelopmental studies limit our ability to fit models with a large amount of covariates. Thus, to avoid having overfit models, we can perform cross-validated methods such as conditional predictive ordinates (CPO) (Pettit 1990, Chen et al. 2012, Lewis et al. 2014). CPO for our model can be defined as $P\left(\mathbf{Y}_i(\mathbf{t}_i) \mid \{\mathbf{Y}_j(\mathbf{t}_j)\}_{j\neq i}\right)$. Unlike traditional cross-validation methods, CPO requires no additional sampling to be conducted. Following Chen et al. (2012) and Lewis et al. (2014), an estimate of CPO for our model can be obtained using the following MCMC approximation:

$$\hat{CPO}_i = \left(\frac{1}{N_{MC}}\sum_{r=1}^{N_{MC}}\frac{1}{P\left(\mathbf{Y}_i(\mathbf{t}_i) \mid \hat{\boldsymbol{\Theta}}^r_{-\chi}, \mathbf{x}_i\right)}\right)^{-1}, \tag{8}$$

where $\hat{\boldsymbol{\Theta}}^r_{-\chi}$ are the samples from the rth MCMC iteration, N_{MC} are the number of MCMC iterations (not including burn-in), and $P\left(\mathbf{Y}_i(\mathbf{t}_i) \mid \hat{\boldsymbol{\Theta}}^r_{-\chi}, \mathbf{x}_i\right)$ is specified in Equation 9 in the main manuscript. While CPO is a measure of how well the model fits each individual observation, the pseudomarginal

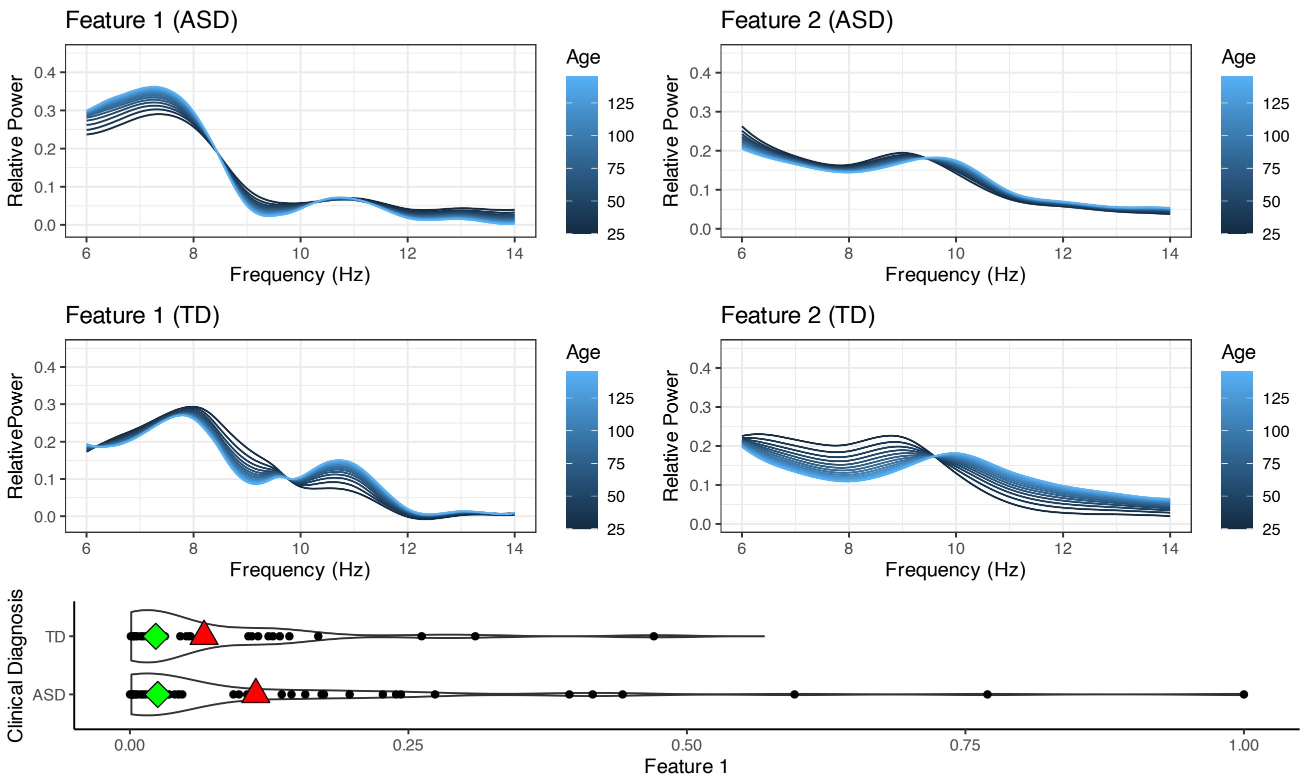

Figure 8: (Top 4 Panels)Estimated mean structures for ASD and TD individuals, for each of the two features used in the covariate adjusted functional mixed membership model fit in Section 4.2 in the main manuscript. (Bottom Panel) Estimates of the allocation parameters stratified by clinical Diagnosis, where the red triangles depict the group level means and the green diamonds depict the group level medians.

likelihood (PML), defined as $P\hat{M}L = \prod_{i=1}^{N} C\hat{P}O_i$, is an overall measure of how well the model fits the entire dataset. Using CPO and PML, we will compare the two covariate adjusted functional mixed membership models fit in this section.

In this section, we will let M_0 denote the covariate adjusted model with age as the covariate, M_1 denote the covariate adjusted functional mixed membership model using the log transform of age as the covariate, and M_2 denote the covariate adjusted functional mixed membership model using the log transform of age, diagnostic group, and the interaction between the log transform of age and diagnostic group as the covariates. Figure 9 contains the CPO values from all 3 models considered. Although the fit is similar between M_0 and M_1, both the pseudomarginal likelihood and the likelihood were higher in the log transformed age model ($M_1 : \log(\mathrm{P\hat{M}L}) = 7391.9$, $\log L = 7833.2$; $M_0 : \log(\mathrm{P\hat{M}L}) = 7390.6$, $\log L = 7826.3$). Thus, the analysis in the main manuscript was performed using the log of age as the covariate. From Figure 9, we can see that M_1 tends to fit the data slightly better than M_2 ($M_1 : \log(\mathrm{P\hat{M}L}) = 7391.9$, $M_2 : \log(\mathrm{P\hat{M}L}) = 7303.9$). Although the fit may be slightly worse for the covariate adjusted model with age and diagnostic group as covariates, this model gives us useful information about how the two features differ between children with ASD and TD children.

4 Mean and Covariance Covariate-dependent Mixed Membership Model

4.1 Model Specification

In this section, we completely specify a mixed membership model where the mean and covariance structures depend on the covariates of interest. As in the main text of this manuscript, we will let $\{\mathbf{Y}_i(.)\}_{i=1}^{N}$ be the observed sample paths and $\mathbf{t}_i = [t_{i1}, \dots, t_{in_i}]^\intercal$ denote the time points at which the ith function was observed. We will also let $\mathbf{X} \in \mathbb{R}^{N \times R}$ denote the design matrix and $\mathbf{x}_i = [X_{i1} \dots X_{iR}]$ denote the ith row of the design matrix (or the covariates associated with the ith

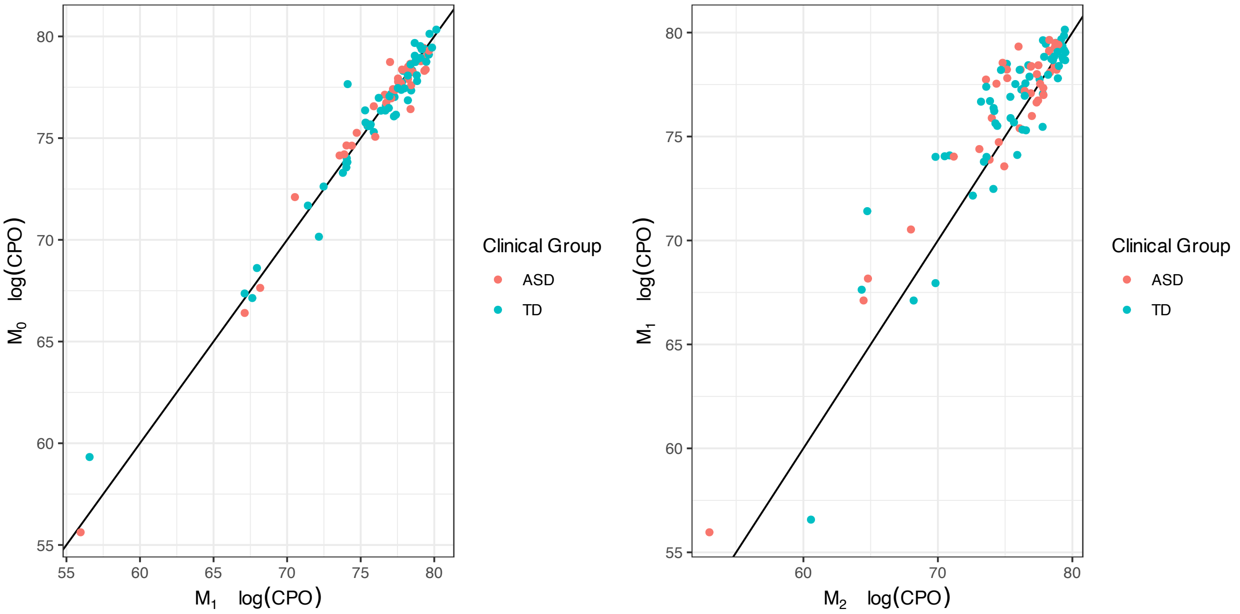

Figure 9: CPO comparisons between different models on the log scale. M_0 denotes the covariate adjusted model with age as the covariate, M_1 denotes the covariate adjusted functional mixed membership model using the log transform of age as the covariate, and M_2 denotes the covariate adjusted functional mixed membership model using the log transform of age, diagnostic group, and the interaction between the log transform of age and diagnostic group as the covariates.

observation). By introducing covariate-dependent pseudo-eigenfunctions, we arrive at the likelihood of our mixed membership model where the mean and covariance structures are dependent on the covariates of interest:

$$\mathbf{Y}_i(\mathbf{t}_i) \mid \boldsymbol{\Theta}, \mathbf{X} \sim \mathcal{N}\left\{\textstyle\sum_{k=1}^{K} Z_{ik}\left(\mathbf{S}^{\intercal}(\mathbf{t}_i)\left(\boldsymbol{\nu}_k + \boldsymbol{\eta}_k \mathbf{x}_i^{\intercal}\right) + \sum_{m=1}^{M} \chi_{im} \mathbf{S}^{\intercal}(\mathbf{t}_i)\left(\boldsymbol{\phi}_{km} + \boldsymbol{\xi}_{km}\mathbf{x}_i^{\intercal}\right)\right),\ \sigma^2 \mathbf{I}_{n_i}\right\}. \quad (9)$$

From Equation 9, we can see that $\boldsymbol{\xi}_{km} \in \mathbb{R}^{P\times R}$, directly controls the effect that the covariates have on the pseudo-eigenfunctions for $k = 1, \dots, K$ and $m = 1, \dots, M$. By integrating out the χ_{im} parameters ($i = 1, \dots, N$ and $m = 1, \dots, M$), we get a model of the following form:

$$\mathbf{Y}_i(\mathbf{t}_i) \mid \boldsymbol{\Theta}_{-\chi}, \mathbf{X} \sim \mathcal{N}\left\{\sum_{k=1}^{K} Z_{ik} \mathbf{S}^{\intercal}(\mathbf{t}_i)\left(\boldsymbol{\nu}_k + \boldsymbol{\eta}_k \mathbf{x}_i^{\intercal}\right),\ \mathbf{V}(\mathbf{t}_i, \mathbf{z}_i) + \sigma^2 \mathbf{I}_{n_i}\right\}, \quad (10)$$

where $\boldsymbol{\Theta}_{-\chi}$ is the collection of our model parameters excluding the χ_{im} variables, and the error-free mixed membership covariance is

$$\mathbf{V}(\mathbf{t}_i, \mathbf{z}_i) = \sum_{k=1}^{K}\sum_{k'=1}^{K} Z_{ik} Z_{ik'}\left\{\mathbf{S}^{\intercal}(\mathbf{t}_i)\sum_{m=1}^{M}\left[\left(\boldsymbol{\phi}_{km} + \boldsymbol{\xi}_{km}\mathbf{x}_i^{\intercal}\right)\left(\boldsymbol{\phi}_{k'm} + \boldsymbol{\xi}_{k'm}\mathbf{x}_i^{\intercal}\right)^{\intercal}\right]\mathbf{S}(\mathbf{t}_i)\right\}. \quad (11)$$

As with the pseudo-eigenfunctions in the unadjusted model, we will utilize the multiplicative gamma process prior as our prior on the $\boldsymbol{\xi}_{km}$ variables. Letting $\xi_{(krm)_p}$ denote the element in the pth row and r^{th} column of $\boldsymbol{\xi}_{km}$. Thus we have:

$$\xi_{(krm)_p} \mid \gamma_{\boldsymbol{\xi}_{krmp}}, \tilde{\tau}_{\boldsymbol{\xi}_{mkr}} \sim \mathcal{N}\left(0, \gamma_{\boldsymbol{\xi}_{krmp}}^{-1}\tilde{\tau}_{\boldsymbol{\xi}_{mkr}}^{-1}\right), \quad \gamma_{\boldsymbol{\xi}_{krmp}} \sim \Gamma\left(\nu_\gamma/2, \nu_\gamma/2\right), \quad \tilde{\tau}_{\boldsymbol{\xi}_{mkr}} = \prod_{n=1}^{m} \delta_{\boldsymbol{\xi}_{nkr}},$$
$$\delta_{\boldsymbol{\xi}_{1kr}} \mid a_{\boldsymbol{\xi}_{1kr}} \sim \Gamma(a_{\boldsymbol{\xi}_{1kr}}, 1), \quad \delta_{\boldsymbol{\xi}_{jkr}} \mid a_{\boldsymbol{\xi}_{2kr}} \sim \Gamma(a_{\boldsymbol{\xi}_{2kr}}, 1), \quad a_{\boldsymbol{\xi}_{1kr}} \sim \Gamma(\alpha_1, \beta_1), \quad a_{\boldsymbol{\xi}_{2kr}} \sim \Gamma(\alpha_2, \beta_2),$$

for $k = 1, \dots, K$, $r = 1, \dots, R$, $m = 1, \dots, M$, and $p = 1, \dots P$. The rest of the parameters in the model have the same prior distributions as the model with the covariate-dependence on the mean structure only in the main text. Specifically, we have

$$\phi_{kpm} | \gamma_{kpm}, \tilde{\tau}_{mk} \sim \mathcal{N}\left(0, \gamma_{kpm}^{-1}\tilde{\tau}_{mk}^{-1}\right), \quad \gamma_{kpm} \sim \Gamma\left(\nu_\gamma/2, \nu_\gamma/2\right), \quad \tilde{\tau}_{mk} = \prod_{n=1}^{m} \delta_{nk},$$

$$\delta_{1k} | a_{1k} \sim \Gamma(a_{1k}, 1), \quad \delta_{jk} | a_{2k} \sim \Gamma(a_{2k}, 1), \quad a_{1k} \sim \Gamma(\alpha_1, \beta_1), \quad a_{2k} \sim \Gamma(\alpha_2, \beta_2),$$

for $k = 1, \ldots, K$, $m = 1, \ldots, M$, and $p = 1, \ldots P$. Similarly, we have

$$P(\boldsymbol{\nu}_k|\tau_{\boldsymbol{\nu}_k}) \propto exp\left(-\frac{\tau_{\boldsymbol{\nu}_k}}{2}\sum_{p=1}^{P-1}\left(\nu_{pk}^\intercal - \nu_{(p+1)k}\right)^2\right),$$

for $k = 1, \ldots, K$, where $\tau_{\boldsymbol{\nu}_k} \sim \Gamma(\alpha_{\boldsymbol{\nu}}, \beta_{\boldsymbol{\nu}})$ and ν_{pk} is the pth element of $\boldsymbol{\nu}_k$. Likewise, we have that

$$P(\{\eta_{prk}\}_{p=1}^P|\tau_{\boldsymbol{\eta}_{rk}}) \propto exp\left(-\frac{\tau_{\boldsymbol{\eta}_{rk}}}{2}\sum_{p=1}^{P-1}\left(\eta_{prk}^\intercal - \eta_{(p+1)rk}\right)^2\right),$$

for $k = 1, \ldots, K$ and $r = 1, \ldots, R$, where $\tau_{\boldsymbol{\eta}_{rk}} \sim \Gamma(\alpha_{\boldsymbol{\eta}}, \beta_{\boldsymbol{\eta}})$ and η_{prk} is the pth row and r^{th} column of $\boldsymbol{\eta}_k$. Lastly, we assume that $\mathbf{z}_i \mid \boldsymbol{\pi}, \alpha_3 \sim_{iid} Dir(\alpha_3\boldsymbol{\pi})$, $\boldsymbol{\pi} \sim Dir(\mathbf{c}_\pi)$, $\alpha_3 \sim Exp(b)$, and $\sigma^2 \sim IG(\alpha_0, \beta_0)$.

4.2 Posterior Distributions

In this subsection, we will specify the posterior distributions specifically for the functional covariate adjusted mixed membership model where the covariance is covariate-dependent. We will first start with the $\boldsymbol{\phi}_{km}$ parameters, for $j = 1, \ldots, K$ and $m = 1, \ldots, M$. Let $\mathbf{D}_{\boldsymbol{\phi}_{jm}} = \tilde{\tau}_{\boldsymbol{\phi}_{mj}}^{-1} diag\left(\gamma_{\boldsymbol{\phi}_{j1m}}^{-1}, \ldots, \gamma_{\boldsymbol{\phi}_{jPm}}^{-1}\right)$. By letting

$$\begin{aligned}\mathbf{m}_{\boldsymbol{\phi}_{jm}} =& \frac{1}{\sigma^2}\sum_{i=1}^{N}\sum_{l=1}^{n_i}\Bigg(B(t_{il})\chi_{im}\Bigg(y_i(t_{il})Z_{ij} - Z_{ij}^2\left(\boldsymbol{\nu}_j + \boldsymbol{\eta}_j\mathbf{x}_i^\intercal\right)^\intercal B(t_{il}) - Z_{ij}^2\sum_{n\neq m}\chi_{in}\boldsymbol{\phi}_{jn}^\intercal B(t_{il}) \\ &- Z_{ij}^2\sum_{n=1}^{M}\chi_{in}\mathbf{x}_i\boldsymbol{\xi}_{jn}^\intercal B(t_{il}) - \sum_{k\neq j} Z_{ij}Z_{ik}\left[(\boldsymbol{\nu}_k + \boldsymbol{\eta}_k\mathbf{x}_i^\intercal)^\intercal B(t_{il}) + \sum_{n=1}^{M}\chi_{in}\left(\boldsymbol{\phi}_{kn} + \boldsymbol{\xi}_{kn}\mathbf{x}_i^\intercal\right)^\intercal B(t_{il})\right]\Bigg)\Bigg),\end{aligned}$$

and

$$\mathbf{M}_{\boldsymbol{\phi}_{jm}}^{-1} = \frac{1}{\sigma^2}\sum_{i=1}^{N}\sum_{l=1}^{n_i}\left(Z_{ij}^2\chi_{im}^2 B(t_{il})B^\intercal(t_{il})\right) + \mathbf{D}_{\boldsymbol{\phi}_{jm}}^{-1},$$

we have that

$$\boldsymbol{\phi}_{jm}|\boldsymbol{\Theta}_{-\boldsymbol{\phi}_{jm}}, \mathbf{Y}_1, \ldots, \mathbf{Y}_N, \mathbf{X} \sim \mathcal{N}\left(\mathbf{M}_{\boldsymbol{\phi}_{jm}}\mathbf{m}_{\boldsymbol{\phi}_{jm}}, \mathbf{M}_{\boldsymbol{\phi}_{jm}}\right).$$

Let $\boldsymbol{\xi}_{krm}$ be the rth column of the matrix $\boldsymbol{\xi}_{km}$. We will let $\mathbf{D}_{\boldsymbol{\xi}_{krm}} = \tilde{\tau}_{\boldsymbol{\xi}_{mjr}}^{-1} diag\left(\gamma_{\boldsymbol{\xi}_{jrm1}}^{-1}, \ldots, \gamma_{\boldsymbol{\phi}_{\boldsymbol{\xi}_{jrmP}}}^{-1}\right)$. We will also let x_{ir} denote the rth element of $\mathbf{x}_i$. Thus, letting

$$\begin{aligned}\mathbf{m}_{\boldsymbol{\xi}_{kdm}} =& \frac{1}{\sigma^2}\sum_{i=1}^{N}\sum_{l=1}^{n_i}\Bigg(B(t_{il})\chi_{im}x_{id}Z_{ik}\Bigg(y_i(t_{il}) - \sum_{j=1}^{K}Z_{ij}\left[\left(\boldsymbol{\nu}_j + \boldsymbol{\eta}_j\mathbf{x}_i^\intercal\right)^\intercal B(t_{il}) + \sum_{n=1}^{M}\chi_{in}\boldsymbol{\phi}_{jn}^\intercal B(t_{il})\right] \\ &- \sum_{(j,n,r)\neq(k,m,d)} Z_{ij}\chi_{in}x_{ir}\boldsymbol{\xi}_{krn}^\intercal B(t_{il})\Bigg)\Bigg)\end{aligned}$$

$$\mathbf{M}_{\boldsymbol{\xi}_{kdm}}^{-1} = \frac{1}{\sigma^2}\sum_{i=1}^{N}\sum_{l=1}^{n_i}\left(Z_{ik}^2\chi_{im}^2 x_{id}^2 B(t_{il})B^\intercal(t_{il})\right) + \mathbf{D}_{\boldsymbol{\xi}_{kdm}}^{-1},$$

we have that

$$\boldsymbol{\xi}_{kdm}|\boldsymbol{\Theta}_{-\boldsymbol{\xi}_{kdm}}, \mathbf{Y}_1, \dots, \mathbf{Y}_N, \mathbf{X} \sim \mathcal{N}\left(\mathbf{M}_{\boldsymbol{\xi}_{kdm}}\mathbf{m}_{\boldsymbol{\xi}_{kdm}}, \mathbf{M}_{\boldsymbol{\xi}_{kdm}}\right).$$

The posterior distribution of $\delta_{\boldsymbol{\phi}_{1k}}$, for $k = 1, \dots, K$, is

$$\begin{aligned}\delta_{\boldsymbol{\phi}_{1k}}|\boldsymbol{\Theta}_{-\delta_{\boldsymbol{\phi}_{1k}}}, \mathbf{Y}_1, \dots, \mathbf{Y}_N, \mathbf{X} \sim &\Gamma\left(a_{\boldsymbol{\phi}_{1k}} + (PM/2), 1 + \frac{1}{2}\sum_{r=1}^{P}\gamma_{\boldsymbol{\phi}_{k,r,1}}\phi_{k,r,1}^2\right. \\ &\left. + \frac{1}{2}\sum_{m=2}^{M}\sum_{r=1}^{P}\gamma_{\boldsymbol{\phi}_{k,r,m}}\phi_{k,r,m}^2\left(\prod_{j=2}^{m}\delta_{\boldsymbol{\phi}_{jk}}\right)\right).\end{aligned}$$

The posterior distribution for $\delta_{\boldsymbol{\phi}_{ik}}$, for $i = 2, \dots, M$ and $k = 1, \dots, K$, is

$$\begin{aligned}\delta_{\boldsymbol{\phi}_{ik}}|\boldsymbol{\Theta}_{-\delta_{\boldsymbol{\phi}_{ik}}}, \mathbf{Y}_1, \dots, \mathbf{Y}_N, \mathbf{X} \sim &\Gamma\left(a_{\boldsymbol{\phi}_{2k}} + (P(M-i+1)/2), 1\right. \\ &\left. + \frac{1}{2}\sum_{m=i}^{M}\sum_{r=1}^{P}\gamma_{\boldsymbol{\xi}_{k,r,m}}\phi_{k,r,m}^2\left(\prod_{j=1;j\neq i}^{m}\delta_{\boldsymbol{\phi}_{jk}}\right)\right).\end{aligned}$$

The posterior distribution of $\delta_{\boldsymbol{\xi}_{1kd}}$, for $k = 1, \dots, K$ and $d = 1, \dots, R$, is

$$\begin{aligned}\delta_{\boldsymbol{\xi}_{1kd}}|\boldsymbol{\Theta}_{-\delta_{\boldsymbol{\xi}_{1kd}}}, \mathbf{Y}_1, \dots, \mathbf{Y}_N, \mathbf{X} \sim &\Gamma\left(a_{\boldsymbol{\xi}_{1kd}} + (PM/2), 1 + \frac{1}{2}\sum_{r=1}^{P}\gamma_{\boldsymbol{\xi}_{kdr1}}\xi_{kdr1}^2\right. \\ &\left. + \frac{1}{2}\sum_{m=2}^{M}\sum_{r=1}^{P}\gamma_{\boldsymbol{\xi}_{kdrm}}\xi_{kdrm}^2\left(\prod_{j=2}^{m}\delta_{\boldsymbol{\xi}_{jkd}}\right)\right).\end{aligned}$$

The posterior distribution for $\delta_{\boldsymbol{\xi}_{ikd}}$, for $i = 2, \dots, M$, $k = 1, \dots, K$, and $d = 1, \dots, D$ is

$$\begin{aligned}\delta_{\boldsymbol{\xi}_{ikd}}|\boldsymbol{\Theta}_{-\delta_{\boldsymbol{\xi}_{ikd}}}, \mathbf{Y}_1, \dots, \mathbf{Y}_N, \mathbf{X} \sim &\Gamma\left(a_{\boldsymbol{\xi}_{2kd}} + (P(M-i+1)/2), 1\right. \\ &\left. + \frac{1}{2}\sum_{m=i}^{M}\sum_{r=1}^{P}\gamma_{\boldsymbol{\xi}_{kdrm}}\phi_{kdrm}^2\left(\prod_{j=1;j\neq i}^{m}\delta_{\boldsymbol{\xi}_{jkd}}\right)\right).\end{aligned}$$

The posterior distribution for $a_{\boldsymbol{\phi}_{1k}}$ $(k = 1, \dots, K)$ is not a commonly known distribution, however we have that

$$P(a_{\boldsymbol{\phi}_{1k}}|\boldsymbol{\Theta}_{-a_{\boldsymbol{\phi}_{1k}}}, \mathbf{Y}_1, \dots, \mathbf{Y}_N, \mathbf{X}) \propto \frac{1}{\Gamma(a_{\boldsymbol{\phi}_{1k}})}\delta_{\boldsymbol{\phi}_{1k}}^{a_{\boldsymbol{\phi}_{1k}}-1}a_{\boldsymbol{\phi}_{1k}}^{\alpha_1-1}exp\left\{-a_{\boldsymbol{\phi}_{1k}}\beta_1\right\}.$$

Since this is not a known kernel of a distribution, we will have to use Metropolis-Hastings algorithm. Consider the proposal distribution $Q(a'_{\phi_{1k}}|a_{\phi_{1k}}) = \mathcal{N}\left(a_{\phi_{1k}}, \epsilon_1\beta_1^{-1}, 0, +\infty\right)$ (Truncated Normal) for some small $\epsilon_1 > 0$. Thus, the probability of accepting any step is

$$A(a'_{\phi_{1k}}, a_{\phi_{1k}}) = \min\left\{1, \frac{P\left(a'_{\phi_{1k}}|\mathbf{\Theta}_{-a'_{\phi_{1k}}}, \mathbf{Y}_1, \ldots, \mathbf{Y}_N, \mathbf{X}\right)}{P\left(a_{\phi_{1k}}|\mathbf{\Theta}_{-a_{\phi_{1k}}}, \mathbf{Y}_1, \ldots, \mathbf{Y}_N, \mathbf{X}\right)} \frac{Q\left(a_{\phi_{1k}}|a'_{\phi_{1k}}\right)}{Q\left(a'_{\phi_{1k}}|a_{\phi_{1k}}\right)}\right\}.$$

Similarly for $a_{\phi_{2k}}$ $(k = 1, \ldots, K)$, we have

$$P(a_{\phi_{2k}}|\mathbf{\Theta}_{-a_{\phi_{2k}}}, \mathbf{Y}_1, \ldots, \mathbf{Y}_N, \mathbf{X}) \propto \frac{1}{\Gamma(a_{\phi_{2k}})^{M-1}} \left(\prod_{i=2}^{M} \delta_{\phi_{ik}}^{a_{\phi_{2k}}-1}\right) a_{\phi_{2k}}^{\alpha_{\phi_{2k}}-1} exp\left\{-a_{\phi_{2k}}\beta_2\right\}.$$

We will use a similar proposal distribution, such that $Q(a'_{\phi_{2k}}|a_{\phi_{2k}}) = \mathcal{N}\left(a_{\phi_{2k}}, \epsilon_2\beta_2^{-1}, 0, +\infty\right)$ for some small $\epsilon_2 > 0$. Thus, the probability of accepting any step is

$$A(a'_{\phi_{2k}}, a_{\phi_{2k}}) = \min\left\{1, \frac{P\left(a'_{\phi_{2k}}|\mathbf{\Theta}_{-a'_{\phi_{2k}}}, \mathbf{Y}_1, \ldots, \mathbf{Y}_N, \mathbf{X}\right)}{P\left(a_{\phi_{2k}}|\mathbf{\Theta}_{-a_{\phi_{2k}}}, \mathbf{Y}_1, \ldots, \mathbf{Y}_N, \mathbf{X}\right)} \frac{Q\left(a_{\phi_{2k}}|a'_{\phi_{2k}}\right)}{Q\left(a'_{\phi_{2k}}|a_{\phi_{2k}}\right)}\right\}.$$

Similarly, the posterior distribution for $a_{\boldsymbol{\xi}_{1kd}}$ $(k = 1, \ldots, K$ and $d = 1, \ldots, R)$ is not a commonly known distribution, however we have that

$$P(a_{\boldsymbol{\xi}_{1kd}}|\mathbf{\Theta}_{-a_{\boldsymbol{\xi}_{1kd}}}, \mathbf{Y}_1, \ldots, \mathbf{Y}_N, \mathbf{X}) \propto \frac{1}{\Gamma(a_{\boldsymbol{\xi}_{1kd}})} \delta_{\boldsymbol{\xi}_{1kd}}^{a_{\boldsymbol{\xi}_{1kd}}-1} a_{\boldsymbol{\xi}_{1kd}}^{\alpha_1-1} exp\left\{-a_{\boldsymbol{\xi}_{1kd}}\beta_1\right\}.$$

We will use a similar proposal distribution, such that $Q(a'_{\boldsymbol{\xi}_{1kd}}|a_{\boldsymbol{\xi}_{1kd}}) = \mathcal{N}\left(a_{\boldsymbol{\xi}_{1kd}}, \epsilon_1\beta_1^{-1}, 0, +\infty\right)$ for some small $\epsilon_1 > 0$. Thus the probability of accepting any step is

$$A(a'_{\boldsymbol{\xi}_{1kd}}, a_{\boldsymbol{\xi}_{1kd}}) = \min\left\{1, \frac{P\left(a'_{\boldsymbol{\xi}_{1kd}}|\mathbf{\Theta}_{-a'_{\boldsymbol{\xi}_{1kd}}}, \mathbf{Y}_1, \ldots, \mathbf{Y}_N, \mathbf{X}\right)}{P\left(a_{\boldsymbol{\xi}_{1kd}}|\mathbf{\Theta}_{-a_{\phi_{1k}}}, \mathbf{Y}_1, \ldots, \mathbf{Y}_N, \mathbf{X}\right)} \frac{Q\left(a_{\boldsymbol{\xi}_{1kd}}|a'_{\boldsymbol{\xi}_{1kd}}\right)}{Q\left(a'_{\boldsymbol{\xi}_{1kd}}|a_{\boldsymbol{\xi}_{1kd}}\right)}\right\}.$$

Similarly for $a_{\boldsymbol{\xi}_{2kd}}$ $(k = 1, \ldots, K$ and $d = 1, \ldots, R)$, we have

$$P(a_{\boldsymbol{\xi}_{2kd}}|\mathbf{\Theta}_{-a_{\boldsymbol{\xi}_{2kd}}}, \mathbf{Y}_1, \ldots, \mathbf{Y}_N, \mathbf{X}) \propto \frac{1}{\Gamma(a_{\boldsymbol{\xi}_{2kd}})^{M-1}} \left(\prod_{i=2}^{M} \delta_{\boldsymbol{\xi}_{ikd}}^{a_{\boldsymbol{\xi}_{2kd}}-1}\right) a_{\boldsymbol{\xi}_{2kd}}^{\alpha_{\boldsymbol{\xi}_{2kd}}-1} exp\left\{-a_{\boldsymbol{\xi}_{2kd}}\beta_2\right\}.$$

We will use a similar proposal distribution, such that $Q(a'_{\boldsymbol{\xi}_{2kd}}|a_{\boldsymbol{\xi}_{2kd}}) = \mathcal{N}\left(a_{\boldsymbol{\xi}_{2kd}}, \epsilon_2\beta_2^{-1}, 0, +\infty\right)$ for some small $\epsilon_2 > 0$. Thus the probability of accepting any step is

$$A(a'_{\boldsymbol{\xi}_{2kd}}, a_{\boldsymbol{\xi}_{2kd}}) = \min\left\{1, \frac{P\left(a'_{\boldsymbol{\xi}_{2kd}}|\mathbf{\Theta}_{-a'_{\boldsymbol{\xi}_{2kd}}}, \mathbf{Y}_1, \ldots, \mathbf{Y}_N, \mathbf{X}\right)}{P\left(a_{\boldsymbol{\xi}_{2kd}}|\mathbf{\Theta}_{-a_{\boldsymbol{\xi}_{2kd}}}, \mathbf{Y}_1, \ldots, \mathbf{Y}_N, \mathbf{X}\right)} \frac{Q\left(a_{\boldsymbol{\xi}_{2kd}}|a'_{\boldsymbol{\xi}_{2kd}}\right)}{Q\left(a'_{\boldsymbol{\xi}_{2kd}}|a_{\boldsymbol{\xi}_{2kd}}\right)}\right\}.$$

For the $\gamma_{\phi_{jrm}}$ parameters, for $j = 1, \ldots K$, $p = 1, \ldots, P$, and $m = 1, \ldots, M$, we have

$$\gamma_{\phi_{jpm}} | \boldsymbol{\Theta}_{-\gamma_{\phi_{jpm}}}, \mathbf{Y}_1, \ldots, \mathbf{Y}_N, \mathbf{X} \sim \Gamma\left(\frac{\nu_\gamma + 1}{2}, \frac{\phi_{jpm}^2 \tilde{\tau}_{\phi_{mj}} + \nu_\gamma}{2}\right).$$

Similarly, for the $\gamma_{\boldsymbol{\xi}_{jdpm}}$ parameters, we have

$$\gamma_{\xi_{jrpm}} | \boldsymbol{\Theta}_{-\gamma_{\xi_{jrpm}}}, \mathbf{Y}_1, \ldots, \mathbf{Y}_N, \mathbf{X} \sim \Gamma\left(\frac{\nu_\gamma + 1}{2}, \frac{\xi_{jrpm}^2 \tilde{\tau}_{\boldsymbol{\xi}_{mjr}} + \nu_\gamma}{2}\right),$$

for $j = 1, \ldots, K$, $r = 1, \ldots, R$, $p = 1, \ldots, P$, and $m = 1, \ldots, M$. The posterior distribution for the $\mathbf{z}_i$ parameters are not a commonly known distribution, so we will use the Metropolis-Hastings algorithm. We know that

$$\begin{aligned} p(\mathbf{z}_i | \boldsymbol{\Theta}_{-\mathbf{z}_i}, \mathbf{Y}_1, \ldots, \mathbf{Y}_N, \mathbf{X}) \propto & \prod_{k=1}^{K} Z_{ik}^{\alpha_3 \pi_k - 1} \\ & \times \prod_{l=1}^{n_i} exp\left\{-\frac{1}{2\sigma^2}\left(y_i(t_{il}) - \sum_{k=1}^{K} Z_{ik}\left((\boldsymbol{\nu}_k + \boldsymbol{\eta}_k \mathbf{x}_i^\intercal)^\intercal B(t_{il})\right.\right.\right. \\ & \left.\left.\left. + \sum_{m=1}^{M} \chi_{im}\left(\boldsymbol{\phi}_{km} + \boldsymbol{\xi}_{km}\mathbf{x}_i^\intercal\right)^\intercal B(t_{il})\right)\right)^2\right\}. \end{aligned}$$

We will use $Q(\mathbf{z}_i'|\mathbf{z}_i) = Dir(a_{\mathbf{z}}\mathbf{z}_i)$ for some large $a_{\mathbf{z}} \in \mathbb{R}^+$ as the proposal distribution. Thus the probability of accepting a proposed step is

$$A(\mathbf{z}_i', \mathbf{z}_i) = \min\left\{1, \frac{P\left(\mathbf{z}_i' | \boldsymbol{\Theta}_{-\mathbf{z}_i}, \mathbf{Y}_1, \ldots, \mathbf{Y}_N, \mathbf{X}\right)}{P\left(\mathbf{z}_i | \boldsymbol{\Theta}_{-\mathbf{z}_i}, \mathbf{Y}_1, \ldots, \mathbf{Y}_N, \mathbf{X}\right)} \frac{Q\left(\mathbf{z}_i | \mathbf{z}_i'\right)}{Q\left(\mathbf{z}_i' | \mathbf{z}_i\right)}\right\}.$$

Similarly, a Gibbs update is not available for an update of the $\boldsymbol{\pi}$ parameters. We have that

$$\begin{aligned} p(\boldsymbol{\pi} | \boldsymbol{\Theta}_{-\boldsymbol{\pi}}, \mathbf{Y}_1, \ldots, \mathbf{Y}_N, \mathbf{X}) \propto & \prod_{k=1}^{K} \pi_k^{c_k - 1} \\ & \times \prod_{i=1}^{N} \frac{1}{B(\alpha_3 \boldsymbol{\pi})} \prod_{k=1}^{K} Z_{ik}^{\alpha_3 \pi_k - 1}. \end{aligned}$$

Letting out proposal distribution be such that $Q(\boldsymbol{\pi}'|\boldsymbol{\pi}) = Dir(a_{\boldsymbol{\pi}}\boldsymbol{\pi})$, for some large $a_{\boldsymbol{\pi}} \in \mathbb{R}^+$, we have that our probability of accepting any proposal is

$$A(\boldsymbol{\pi}', \boldsymbol{\pi}) = \min\left\{1, \frac{P\left(\boldsymbol{\pi}' | \boldsymbol{\Theta}_{-\boldsymbol{\pi}'}, \mathbf{Y}_1, \ldots, \mathbf{Y}_N, \mathbf{X}\right)}{P\left(\boldsymbol{\pi} | \boldsymbol{\Theta}_{-\boldsymbol{\pi}}, \mathbf{Y}_1, \ldots, \mathbf{Y}_N, \mathbf{X}\right)} \frac{Q\left(\boldsymbol{\pi} | \boldsymbol{\pi}'\right)}{Q\left(\boldsymbol{\pi}' | \boldsymbol{\pi}\right)}\right\}.$$

The posterior distribution of α_3 is also not a commonly known distribution, so we will use the Metropolis-Hastings algorithm to sample from the posterior distribution. We have that

$$\begin{aligned} p(\alpha_3 | \boldsymbol{\Theta}_{-\alpha_3}, \mathbf{Y}_1, \ldots, \mathbf{Y}_N, \mathbf{X}) \propto & \, e^{-b\alpha_3} \\ & \times \prod_{i=1}^{N} \frac{1}{B(\alpha_3 \boldsymbol{\pi})} \prod_{k=1}^{K} Z_{ik}^{\alpha_3 \pi_k - 1}. \end{aligned}$$

Using a proposal distribution such that $Q(\alpha_3'|\alpha_3) = \mathcal{N}(\alpha_3, \sigma^2_{\alpha_3}, 0, +\infty)$ (Truncated Normal), we are left with the probability of accepting a proposed state as

$$A(\alpha_3', \alpha_3) = \min\left\{1, \frac{P\left(\alpha_3'|\boldsymbol{\Theta}_{-\alpha_3'}, \mathbf{Y}_1, \ldots, \mathbf{Y}_N, \mathbf{X}\right)}{P\left(\alpha_3|\boldsymbol{\Theta}_{-\alpha_3}, \mathbf{Y}_1, \ldots, \mathbf{Y}_N, \mathbf{X}\right)} \frac{Q\left(\alpha_3|\alpha_3'\right)}{Q\left(\alpha_3'|\alpha_3\right)}\right\}.$$

Let $\mathbf{P}$ be the following tridiagonal matrix:

$$\mathbf{P} = \begin{bmatrix} 1 & -1 & 0 & & \\ -1 & 2 & -1 & & \\ & \ddots & \ddots & \ddots & \\ & & -1 & 2 & -1 \\ & & 0 & -1 & 1 \end{bmatrix}.$$

Thus, letting

$$\mathbf{B}_{\boldsymbol{\nu}_j} = \left(\tau_{\boldsymbol{\nu}_j}\mathbf{P} + \frac{1}{\sigma^2}\sum_{i=1}^{N}\sum_{l=1}^{n_i} Z_{ij}^2 B(t_{il})B^\intercal(t_{il})\right)^{-1}$$

and

$$\begin{aligned}\mathbf{b}_{\boldsymbol{\nu}_j} = \frac{1}{\sigma^2}\sum_{i=1}^{N}\sum_{l=1}^{n_i} Z_{ij}B(t_{il})&\left[y_i(t_{il}) - \left(\sum_{k\neq j} Z_{ik}\boldsymbol{\nu}_k^\intercal B(t_{il})\right)\right. \\ &\left. - \left(\sum_{k=1}^{K} Z_{ik}\left[\mathbf{x}_i\boldsymbol{\eta}_k^\intercal B(t_{il}) + \sum_{m=1}^{M}\chi_{im}\left(\boldsymbol{\phi}_{kn} + \boldsymbol{\xi}_{kn}\mathbf{x}_i^\intercal\right)^\intercal B(t_{il})\right]\right)\right],\end{aligned}$$

we have that

$$\boldsymbol{\nu}_j|\boldsymbol{\Theta}_{-\boldsymbol{\nu}_j}, \mathbf{Y}_1, \ldots, \mathbf{Y}_N, \mathbf{X} \sim \mathcal{N}\left(\mathbf{B}_{\boldsymbol{\nu}_j}\mathbf{b}_{\boldsymbol{\nu}_j}, \mathbf{B}_{\boldsymbol{\nu}_j}\right).$$

Let $\boldsymbol{\eta}_{jd}$ denote the dth column of the matrix $\boldsymbol{\eta}_j$. Thus, letting

$$\mathbf{B}_{\boldsymbol{\eta}_{jd}} = \left(\tau_{\boldsymbol{\eta}_{jd}}\mathbf{P} + \frac{1}{\sigma^2}\sum_{i=1}^{N}\sum_{l=1}^{n_i} Z_{ij}^2 x_{id}^2 B(t_{il})B^\intercal(t_{il})\right)^{-1}$$

and

$$\begin{aligned}\mathbf{b}_{\boldsymbol{\eta}_{jd}} =& \frac{1}{\sigma^2}\sum_{i=1}^{N}\sum_{l=1}^{n_i} Z_{ij}x_{id}B(t_{il})\left[y_i(t_{il}) - \left(\sum_{r\neq d} Z_{ij}x_{ir}\boldsymbol{\eta}_{jr}^\intercal B(t_{il})\right) - \left(\sum_{k\neq j} Z_{ik}\mathbf{x}_i\boldsymbol{\eta}_k^\intercal B(t_{il})\right)\right. \\ &\left. - \left(\sum_{k=1}^{K} Z_{ik}\left[\boldsymbol{\nu}_k^\intercal B(t_{il}) + \sum_{m=1}^{M}\chi_{im}\left(\boldsymbol{\phi}_{kn} + \boldsymbol{\xi}_{kn}\mathbf{x}_i^\intercal\right)^\intercal B(t_{il})\right]\right)\right],\end{aligned}$$

we have that

$$\boldsymbol{\eta}_{jd}|\boldsymbol{\Theta}_{-\boldsymbol{\eta}_{jd}}, \mathbf{Y}_1, \ldots, \mathbf{Y}_N, \mathbf{X} \sim \mathcal{N}\left(\mathbf{B}_{\boldsymbol{\eta}_{jd}}\mathbf{b}_{\boldsymbol{\eta}_{jd}}, \mathbf{B}_{\boldsymbol{\eta}_{jd}}\right).$$

Thus we can see that we can draw samples from the posterior of the parameters controlling the mean structure using a Gibbs sampler. Similarly, we can use a Gibbs sampler to draw samples from the posterior distribution of $\tau_{\boldsymbol{\eta}_{jd}}$ and $\tau_{\boldsymbol{\nu}_j}$. We have that the posterior distributions are

$$\tau_{\boldsymbol{\nu}_j} | \boldsymbol{\Theta}_{-\tau_{\boldsymbol{\nu}_j}}, \mathbf{Y}_1, \dots, \mathbf{Y}_N, \mathbf{X} \sim \Gamma\left(\alpha_{\boldsymbol{\nu}} + P/2, \beta_{\boldsymbol{\nu}} + \frac{1}{2}\boldsymbol{\nu}_j^\intercal \mathbf{P} \boldsymbol{\nu}_j\right)$$

and

$$\tau_{\boldsymbol{\eta}_{jd}} | \boldsymbol{\Theta}_{-\tau_{\boldsymbol{\eta}_{jd}}}, \mathbf{Y}_1, \dots, \mathbf{Y}_N, \mathbf{X} \sim \Gamma\left(\alpha_{\boldsymbol{\eta}} + P/2, \beta_{\boldsymbol{\eta}} + \frac{1}{2}\boldsymbol{\eta}_{jd}^\intercal \mathbf{P} \boldsymbol{\eta}_{jd}\right),$$

for $j = 1, \dots, K$ and $d = 1, \dots, R$. The parameter σ^2 can be updated using a Gibbs update. If we let

$$\beta_\sigma = \frac{1}{2}\sum_{i=1}^{N}\sum_{l=1}^{n_i}\left(y_i(t_{il}) - \sum_{k=1}^{K} Z_{ik}\left((\boldsymbol{\nu}_k + \boldsymbol{\eta}_k \mathbf{x}_i^\intercal)^\intercal B(t_{il}) + \sum_{n=1}^{M} \chi_{in}\left(\boldsymbol{\phi}_{kn} + \boldsymbol{\xi}_{kn}\mathbf{x}_i^\intercal\right)^\intercal B(t_{il})\right)\right)^2,$$

then we have

$$\sigma^2 | \boldsymbol{\Theta}_{-\sigma^2}, \mathbf{Y}_1, \dots, \mathbf{Y}_N, \mathbf{X} \sim IG\left(\alpha_0 + \frac{\sum_{i=1}^{N} n_i}{2}, \beta_0 + \beta_\sigma\right).$$

Lastly, we can update the χ_{im} parameters, for $i = 1, \dots, N$ and $m = 1, \dots, M$, using a Gibbs update. If we let

$$\begin{aligned}\mathbf{w}_{im} =& \frac{1}{\sigma^2}\left[\sum_{l=1}^{n_i}\left(\sum_{k=1}^{K} Z_{ik}\left(\boldsymbol{\phi}_{km} + \boldsymbol{\xi}_{km}\mathbf{x}_i^\intercal\right)^\intercal B(t_{il})\right)\right.\\ &\left.\left(y_i(t_{il}) - \sum_{k=1}^{K} Z_{ik}\left((\boldsymbol{\nu}_k + \boldsymbol{\eta}_k\mathbf{x}_i^\intercal)^\intercal B(t_{il}) + \sum_{n\neq m}\chi_{in}\left(\boldsymbol{\phi}_{kn} + \boldsymbol{\xi}_{kn}\mathbf{x}_i^\intercal\right)^\intercal B(t_{il})\right)\right)\right]\end{aligned}$$

and

$$\mathbf{W}_{im}^{-1} = 1 + \frac{1}{\sigma^2}\sum_{l=1}^{n_i}\left(\sum_{k=1}^{K} Z_{ik}\left(\boldsymbol{\phi}_{km} + \boldsymbol{\xi}_{km}\mathbf{x}_i^\intercal\right)^\intercal B(t_{il})\right)^2,$$

then we have that

$$\chi_{im} | \boldsymbol{\zeta}_{-\chi_{im}}, \mathbf{Y}_1, \dots, \mathbf{Y}_N, \mathbf{X} \sim \mathcal{N}(\mathbf{W}_{im}\mathbf{w}_{im}, \mathbf{W}_{im}).$$

4.3 Tempered Transitions

Since we only temper the likelihood, many of the posterior distributions derived in Section 4.2 can be utilized. Starting with the $\mathbf{\Phi}$ parameters, we have

$$\begin{aligned}\left(\mathbf{m}_{\phi_{jm}}\right)_h =& \frac{\beta_h}{(\sigma^2)_h}\sum_{i=1}^{N}\sum_{l=1}^{n_i}\Big(B(t_{il})(\chi_{im})_h\Big(y_i(t_{il})(Z_{ij})_h - (Z_{ij})_h^2\left((\boldsymbol{\nu}_j)_h + (\boldsymbol{\eta}_j)_h\mathbf{x}_i^\intercal\right)^\intercal B(t_{il})\\ &-(Z_{ij})_h^2\sum_{n\neq m}(\chi_{in})_h(\boldsymbol{\phi}_{jn})_h^\intercal B(t_{il}) - (Z_{ij})_h^2\sum_{n=1}^{M}(\chi_{in})\mathbf{x}_i(\boldsymbol{\xi}_{jn})_h^\intercal B(t_{il})\\ &-\sum_{k\neq j}Z_{ij}Z_{ik}\left[((\boldsymbol{\nu}_k)_h + (\boldsymbol{\eta}_k)_h\mathbf{x}_i^\intercal)^\intercal B(t_{il}) + \sum_{n=1}^{M}\chi_{in}\left((\boldsymbol{\phi}_{kn})_h + (\boldsymbol{\xi}_{kn})_h\mathbf{x}_i^\intercal\right)^\intercal B(t_{il})\right]\Big)\Big),\end{aligned}$$

and

$$\left(\mathbf{M}_{\phi_{jm}}\right)_h^{-1} = \frac{\beta_h}{(\sigma^2)_h}\sum_{i=1}^{N}\sum_{l=1}^{n_i}\left((Z_{ij})_h^2(\chi_{im})_h^2 B(t_{il})B^\intercal(t_{il})\right) + \left(\mathbf{D}_{\phi_{jm}}\right)_h^{-1},$$

we have that

$$\left(\boldsymbol{\phi}_{jm}\right)_h|\mathbf{\Theta}_{-\left(\phi_{jm}\right)_h}, \mathbf{Y}_1,\dots,\mathbf{Y}_N,\mathbf{X}\sim\mathcal{N}\left(\left(\mathbf{M}_{\phi_{jm}}\right)_h\left(\mathbf{m}_{\phi_{jm}}\right)_h, \left(\mathbf{M}_{\phi_{jm}}\right)_h\right).$$

Letting

$$\begin{aligned}\left(\mathbf{m}_{\boldsymbol{\xi}_{kdm}}\right)_h =& \frac{\beta_h}{(\sigma^2)_h}\sum_{i=1}^{N}\sum_{l=1}^{n_i}\Bigg(B(t_{il})(\chi_{im})_h x_{id}(Z_{ik})_h\Bigg(y_i(t_{il}) -\\ &\sum_{j=1}^{K}(Z_{ij})_h\left[\left((\boldsymbol{\nu}_j)_h + (\boldsymbol{\eta}_j)_h\mathbf{x}_i^\intercal\right)^\intercal B(t_{il}) + \sum_{n=1}^{M}(\chi_{in})_h(\boldsymbol{\phi}_{jn})_h^\intercal B(t_{il})\right]\\ &-\sum_{(j,n,r)\neq(k,m,d)}(Z_{ij})_h(\chi_{in})_h x_{ir}(\boldsymbol{\xi}_{krn})_h^\intercal B(t_{il})\Bigg)\Bigg)\end{aligned}$$

$$\left(\mathbf{M}_{\boldsymbol{\xi}_{kdm}}\right)_h^{-1} = \frac{\beta_h}{(\sigma^2)_h}\sum_{i=1}^{N}\sum_{l=1}^{n_i}\left((Z_{ik})_h^2(\chi_{im})_h^2 x_{id}^2 B(t_{il})B^\intercal(t_{il})\right) + \left(\mathbf{D}_{\boldsymbol{\xi}_{kdm}}\right)_h^{-1},$$

we have that

$$(\boldsymbol{\xi}_{kdm})_h|\mathbf{\Theta}_{-(\boldsymbol{\xi}_{kdm})_h}, \mathbf{Y}_1,\dots,\mathbf{Y}_N,\mathbf{X}\sim\mathcal{N}\left(\left(\mathbf{M}_{\boldsymbol{\xi}_{kdm}}\right)_h\left(\mathbf{m}_{\boldsymbol{\xi}_{kdm}}\right)_h, \left(\mathbf{M}_{\boldsymbol{\xi}_{kdm}}\right)_h\right).$$

As in the untempered case, we have that the posterior distribution $\mathbf{Z}$ parameters under the tempered likelihood is not a commonly known distribution. Therefore, we will use the Metropolis-Hastings

algorithm. We have that

$$p((\mathbf{z}_i)_h|\boldsymbol{\Theta}_{-(\mathbf{z}_i)_h}, \mathbf{Y}_1, \dots, \mathbf{Y}_N, \mathbf{X}) \propto \prod_{k=1}^{K} (Z_{ik})_h^{(\alpha_3)_h(\pi_k)_h - 1}$$
$$\times \prod_{l=1}^{n_i} exp\left\{ -\frac{\beta_h}{2(\sigma^2)_h} \left(y_i(t_{il}) - \sum_{k=1}^{K} (Z_{ik})_h \left(((\boldsymbol{\nu}_k)_h + (\boldsymbol{\eta}_k)_h \mathbf{x}_i^\intercal)^\intercal B(t_{il}) \right.\right.\right.$$
$$\left.\left.\left. + \sum_{m=1}^{M} (\chi_{im})_h \left((\boldsymbol{\phi}_{km})_h + (\boldsymbol{\xi}_{km})_h \mathbf{x}_i^\intercal \right)^\intercal B(t_{il}) \right) \right)^2 \right\}.$$

We will use $Q((\mathbf{z}_i)'_h|(\mathbf{z}_i)_h) = Dir(a_{\mathbf{z}}(\mathbf{z}_i)_h)$ for some large $a_{\mathbf{z}} \in \mathbb{R}^+$ as the proposal distribution. Thus, the probability of accepting a proposed step is

$$A((\mathbf{z}_i)'_h, (\mathbf{z}_i)_h) = \min\left\{ 1, \frac{P\left((\mathbf{z}_i)'_h|\boldsymbol{\Theta}_{-(\mathbf{z}_i)'_h}, \mathbf{Y}_1, \dots, \mathbf{Y}_N, \mathbf{X}\right)}{P\left((\mathbf{z}_i)_h|\boldsymbol{\Theta}_{-(\mathbf{z}_i)_h}, \mathbf{Y}_1, \dots, \mathbf{Y}_N, \mathbf{X}\right)} \frac{Q\left((\mathbf{z}_i)_h|(\mathbf{z}_i)'_h\right)}{Q\left((\mathbf{z}_i)'_h|(\mathbf{z}_i)_h\right)} \right\}.$$

Letting

$$\left(\mathbf{B}_{\boldsymbol{\nu}_j}\right)_h = \left(\left(\tau_{\boldsymbol{\nu}_j}\right)_h \mathbf{P} + \frac{\beta_h}{(\sigma^2)_h} \sum_{i=1}^{N} \sum_{l=1}^{n_i} (Z_{ij})_h^2 B(t_{il}) B^\intercal(t_{il}) \right)^{-1}$$

and

$$\left(\mathbf{b}_{\boldsymbol{\nu}_j}\right)_h = \frac{\beta_h}{(\sigma^2)_h} \sum_{i=1}^{N} \sum_{l=1}^{n_i} (Z_{ij})_h B(t_{il}) \left[y_i(t_{il}) - \left(\sum_{k \neq j} (Z_{ik})_h (\boldsymbol{\nu}_k^\intercal)_h B(t_{il}) \right) \right.$$
$$\left. - \left(\sum_{k=1}^{K} (Z_{ik}) \left[\mathbf{x}_i (\boldsymbol{\eta}_k)_h^\intercal B(t_{il}) + \sum_{m=1}^{M} (\chi_{im})_h \left((\boldsymbol{\phi}_{kn})_h + (\boldsymbol{\xi}_{kn})_h \mathbf{x}_i^\intercal \right)^\intercal B(t_{il}) \right] \right) \right],$$

we have that

$$(\boldsymbol{\nu}_j)_h | \boldsymbol{\Theta}_{-(\boldsymbol{\nu}_j)_h}, \mathbf{Y}_1, \dots, \mathbf{Y}_N, \mathbf{X} \sim \mathcal{N}\left(\left(\mathbf{B}_{\boldsymbol{\nu}_j}\right)_h \left(\mathbf{b}_{\boldsymbol{\nu}_j}\right)_h, \left(\mathbf{B}_{\boldsymbol{\nu}_j}\right)_h \right).$$

Let $\left(\boldsymbol{\eta}_{jd}\right)_h$ denote the dth column of the matrix $(\boldsymbol{\eta}_j)_h$. Thus, letting

$$\left(\mathbf{B}_{\boldsymbol{\eta}_{jd}}\right)_h = \left(\left(\tau_{\boldsymbol{\eta}_{jd}}\right)_h \mathbf{P} + \frac{\beta_h}{(\sigma^2)_h} \sum_{i=1}^{N} \sum_{l=1}^{n_i} (Z_{ij})_h^2 x_{id}^2 B(t_{il}) B^\intercal(t_{il}) \right)^{-1}$$

and

$$\left(\mathbf{b}_{\boldsymbol{\eta}_{jd}}\right)_h = \frac{\beta_h}{(\sigma^2)_h} \sum_{i=1}^{N} \sum_{l=1}^{n_i} (Z_{ij})_h x_{id} B(t_{il}) \left[y_i(t_{il}) - \left(\sum_{r \neq d} (Z_{ij})_h x_{ir} (\boldsymbol{\eta}_{jr})_h^\intercal B(t_{il}) \right) \right.$$
$$- \left(\sum_{k \neq j} (Z_{ik})_h \mathbf{x}_i (\boldsymbol{\eta}_k)_h^\intercal B(t_{il}) \right)$$
$$\left. - \left(\sum_{k=1}^{K} (Z_{ik})_h \left[(\boldsymbol{\nu}_k)_h^\intercal B(t_{il}) + \sum_{m=1}^{M} (\chi_{im})_h \left((\boldsymbol{\phi}_{kn})_h + (\boldsymbol{\xi}_{kn})_h \mathbf{x}_i^\intercal \right)^\intercal B(t_{il}) \right] \right) \right],$$

we have that

$$\left(\boldsymbol{\eta}_{jd}\right)_h|\boldsymbol{\Theta}_{-\left(\boldsymbol{\eta}_{jd}\right)_h}, \mathbf{Y}_1, \ldots, \mathbf{Y}_N, \mathbf{X} \sim \mathcal{N}\left(\left(\mathbf{B}_{\boldsymbol{\eta}_{jd}}\right)_h \left(\mathbf{b}_{\boldsymbol{\eta}_{jd}}\right)_h, \left(\mathbf{B}_{\boldsymbol{\eta}_{jd}}\right)_h\right).$$

If we let

$$\begin{aligned}(\beta_\sigma)_h = \frac{\beta_h}{2}\sum_{i=1}^{N}\sum_{l=1}^{n_i}\Bigg(y_i(t_{il}) - \sum_{k=1}^{K}(Z_{ik})_h\Bigg(&((\boldsymbol{\nu}_k)_h + (\boldsymbol{\eta}_k)_h\mathbf{x}_i^\intercal)^\intercal B(t_{il}) \\ &+ \sum_{n=1}^{M}(\chi_{in})_h\left((\boldsymbol{\phi}_{kn})_h + (\boldsymbol{\xi}_{kn})_h\mathbf{x}_i^\intercal\right)^\intercal B(t_{il})\Bigg)\Bigg)^2,\end{aligned}$$

then we have

$$(\sigma^2)_h|\boldsymbol{\Theta}_{-(\sigma^2)_h}, \mathbf{Y}_1, \ldots, \mathbf{Y}_N, \mathbf{X} \sim IG\left(\alpha_0 + \frac{\beta_h \sum_{i=1}^N n_i}{2}, \beta_0 + (\beta_\sigma)_h\right).$$

Lastly, we can update the χ_{im} parameters, for $i = 1, \ldots, N$ and $m = 1, \ldots, M$, using a Gibbs update. If we let

$$\begin{aligned}(\mathbf{w}_{im})_h =& \frac{\beta_h}{(\sigma^2)_h}\left[\sum_{l=1}^{n_i}\left(\sum_{k=1}^{K}(Z_{ik})_h\left((\boldsymbol{\phi}_{km})_h + (\boldsymbol{\xi}_{km})_h\mathbf{x}_i^\intercal\right)^\intercal B(t_{il})\right)\Bigg(y_i(t_{il})\right. \\ &\left. - \sum_{k=1}^{K}(Z_{ik})_h\left(((\boldsymbol{\nu}_k)_h + (\boldsymbol{\eta}_k)_h\mathbf{x}_i^\intercal)^\intercal B(t_{il}) + \sum_{n\neq m}(\chi_{in})_h\left((\boldsymbol{\phi}_{kn})_h + (\boldsymbol{\xi}_{kn})_h\mathbf{x}_i^\intercal\right)^\intercal B(t_{il})\right)\Bigg)\right]\end{aligned}$$

and

$$(\mathbf{W}_{im})_h^{-1} = 1 + \frac{\beta_h}{\sigma^2}\sum_{l=1}^{n_i}\left(\sum_{k=1}^{K}(Z_{ik})_h\left((\boldsymbol{\phi}_{km})_h + (\boldsymbol{\xi}_{km})_h\mathbf{x}_i^\intercal\right)^\intercal B(t_{il})\right)^2,$$

then we have that

$$(\chi_{im})_h|\boldsymbol{\zeta}_{-(\chi_{im})_h}, \mathbf{Y}_1, \ldots, \mathbf{Y}_N, \mathbf{X} \sim \mathcal{N}\left((\mathbf{W}_{im})_h(\mathbf{w}_{im})_h, (\mathbf{W}_{im})_h\right).$$

5 Relationship to Function-on-Scalar Regression

Function-on-scalar regression is a common method in FDA that allows the mean structure of a continuous stochastic process to be dependent on scalar covariates. In function-on-scalar regression, we often assume that the response is a GP, and that the covariates of interest are vector-valued. A comprehensive review of the broader area of functional regression can be found in Ramsay & Silverman (2005) and Morris (2015). While there have been many advancements and generalizations

(Krafty et al. 2008, Reiss et al. 2010, Goldsmith et al. 2015, Kowal & Bourgeois 2020) of function-on-scalar regression since the initial papers of Faraway (1997) and Brumback & Rice (1998), the general form of function-on-scalar regression can be expressed as follows:

$$Y(t) = \mu(t) + \sum_{r=1}^{R} X_r \beta_r(t) + \epsilon(t); \quad t \in \mathcal{T}, \tag{12}$$

where $Y(t)$ is the response function evaluated at t, $\beta_r(\cdot)$ is the functional coefficient representing the effect that the rth covariate (X_r) has on the mean structure, and ϵ is a mean-zero Gaussian process with covariance function $\mathcal{C}$. The function $\mu : \mathcal{T} \rightarrow \mathbb{R}$ in Equation (12) represents the mean of the GP when all of the covariates, X_{ir}, are set to zero. Unlike the traditional setting for multiple linear regression in finite-dimensional vector spaces, function-on-scalar regression requires the estimation of the infinite-dimensional functions μ and $\beta_1, \dots, \beta_R$ from a finite number of observed sample paths at a finite number of points ($\mathbf{Y}_i(\mathbf{t}_i)$ for $i = 1, \dots, N$ and $\mathbf{t}_i = [t_{i1}, \dots, t_{in_i}]^\intercal$).

To make inference tractable, we assume that the data lie in the span of a finite set of basis functions, which will allow us to expand μ and $\beta_1, \dots, \beta_R$ as a finite sum of the basis functions. The set of basis functions can be specified using data-driven basis functions or by specifying the basis functions *a-priori*. If the basis functions are specified *a-priori*, common choices of basis functions are B-splines and wavelets due to their flexibility, as well as Fourier series for periodic functions. Alternatively, if the use of data-driven basis functions is desired, a common choice is to use the eigenfunctions of the covariance operator as basis functions. In order to estimate the eigenfunctions, functional principal component analysis (Shang 2014) is often performed and the obtained estimates of the eigenfunctions are used. Functional principal component analysis faces a similar problem in that the objective is to estimate eigenfunctions using only a finite number of sample paths observed at a finite number of points. To solve this problem, Rice & Silverman (1991) proposes using a splines basis to estimate smooth eigenfunctions, while Yao et al. (2005) proposes using local linear smoothers to estimate the smooth eigenfunctions. Therefore, even using data-driven basis functions requires smoothing assumptions, suggesting similar results between data-driven basis functions and a reasonable set of *a-priori* specified basis functions paired with a penalty to

prevent overfitting.

Specifying the basis functions *a-priori* $(b_1(t), \dots, b_P(t))$, and letting $\mathbf{Y}_i(\mathbf{t}_i)$ be the observed sample paths at points $\mathbf{t}_i = [t_{i1}, \dots, t_{in_i}]^\intercal$ $(i = 1, \dots, N)$, we can simplify Equation (12) to get

$$\mathbf{Y}_i(\mathbf{t}_i) = \mathbf{S}^\intercal(\mathbf{t}_i)\tilde{\boldsymbol{\nu}} + \mathbf{S}^\intercal(\mathbf{t}_i)\tilde{\boldsymbol{\eta}}\mathbf{x}_i^\intercal + \boldsymbol{\epsilon}_i(\mathbf{t}_i), \tag{13}$$

where $\tilde{\boldsymbol{\nu}} \in \mathbb{R}^P$, $\tilde{\boldsymbol{\eta}} \in \mathbb{R}^{P\times R}$, and $\mathbf{S}(\mathbf{t}_i) = [\mathbf{B}(t_1)\cdots\mathbf{B}(t_{n_i})] \in \mathbb{R}^{P\times n_i}$ are the set of basis functions evaluated at the time points of interest. As specified in the previous sections, $\mathbf{B}^\intercal(t) := [b_1(t), b_2(t), \dots, b_P(t)]$. Equation (13) shows that the function $\mu(.)$ evaluated at the points $\mathbf{t}_i$ can be represented by $\mathbf{S}^\intercal(\mathbf{t}_i)\tilde{\boldsymbol{\nu}}$, and similarly the functional coefficients $\beta_1(\cdot), \dots, \beta_R(\cdot)$ can be represented by $\mathbf{S}^\intercal(\mathbf{t}_i)\tilde{\boldsymbol{\eta}}$. Therefore, we are left to estimate $\tilde{\boldsymbol{\nu}}$, $\tilde{\boldsymbol{\eta}}$, and the parameters associated with the covariance function of $\epsilon(\cdot)$, denoted $\mathcal{C}$.

The covariance function $\mathcal{C}$ represents the within-function covariance structure of the data. In the simplest case, we often assume that $\boldsymbol{\epsilon}_i(\mathbf{t}_i) \sim \mathcal{N}(\mathbf{0}, \tilde{\sigma}^2\mathbf{I}_{n_i})$, which means that we only need $\tilde{\sigma}^2$ to specify $\mathcal{C}$. In more complex models (Faraway 1997, Krafty et al. 2008), one may make less restrictive assumptions and assume that the covariance $\boldsymbol{\epsilon}_i(\mathbf{t}_i) \sim \mathcal{N}(\mathbf{0}_{n_i}, \tilde{V}(\mathbf{t}_i) + \tilde{\sigma}^2\mathbf{I}_{n_i})$, where $\tilde{V}(\cdot)$ is a low-dimensional approximation of a smooth covariance surface using a truncated eigendecomposition. Although functional regression usually assumes that the functions are independent, functional regression models have been proposed to model between-function variation, or cases where observations can be correlated (Morris & Carroll 2006, Staicu et al. 2010).

Assuming a relatively general covariance structure as in Krafty et al. (2008), the function-on-scalar model assumes the following distributional assumptions on our sample paths:

$$\mathbf{Y}_i(\mathbf{t}_i) \mid \tilde{\boldsymbol{\nu}}, \tilde{\boldsymbol{\eta}}, \tilde{\mathbf{V}}(\mathbf{t}_i), \tilde{\sigma}^2, \mathbf{X} \sim \mathcal{N}\left\{\mathbf{S}^\intercal(\mathbf{t}_i)\left(\tilde{\boldsymbol{\nu}} + \tilde{\boldsymbol{\eta}}\mathbf{x}_i^\intercal\right),\ \tilde{\mathbf{V}}(\mathbf{t}_i) + \tilde{\sigma}^2\mathbf{I}_{n_i}\right\}. \tag{14}$$

From Equation (14) it is apparent that the proposed covariate adjusted mixed membership model specified in Equation 9 in the main text is closely related to function-on-scalar regression. The key difference between the two models is that the covariate adjusted mixed membership model does not assume a common mean and covariance structure across all observations conditionally on

the covariates of interest. Instead, the covariate adjusted mixed membership model allows each observation to be modeled as a convex combination of the K underlying features. Each feature is allowed to have different mean and covariance structures, meaning that the covariates are not assumed to have the same affect on all observations. By allowing this type of heterogeneity in our model, we can conduct a more granular analysis and identify subgroups that interact differently with the covariates of interest. Alternatively, if there are subgroups of the population that interact differently with the covariates of interest, then the results from a function-on-scalar regression model will be confounded, as the effects will likely be averaged out in the analysis. The finite mixture of experts model and the mixture of regressions model offer a reasonably granular level of insight, permitting inference at the sub-population level. However, they do not provide the individual-level inference achievable with the proposed covariate adjusted functional mixed membership model.

References

Akaike, H. (1974), 'A new look at the statistical model identification', *IEEE transactions on automatic control* **19**(6), 716–723.

Behrens, G., Friel, N. & Hurn, M. (2012), 'Tuning tempered transitions', *Statistics and computing* **22**(1), 65–78.

Brumback, B. A. & Rice, J. A. (1998), 'Smoothing spline models for the analysis of nested and crossed samples of curves', *Journal of the American Statistical Association* **93**(443), 961–976.

Celeux, G., Forbes, F., Robert, C. P. & Titterington, D. M. (2006), 'Deviance information criteria for missing data models', *Bayesian analysis* **1**(4), 651–673.

Chen, M.-H., Shao, Q.-M. & Ibrahim, J. G. (2012), *Monte Carlo methods in Bayesian computation*, Springer Science & Business Media.

Chen, Y., He, S., Yang, Y. & Liang, F. (2022), 'Learning topic models: Identifiability and finite-sample analysis', *Journal of the American Statistical Association* pp. 1–16.

Dickinson, A., DiStefano, C., Senturk, D. & Jeste, S. S. (2018), 'Peak alpha frequency is a neural marker of cognitive function across the autism spectrum', *European Journal of Neuroscience* **47**(6), 643–651.

Faraway, J. J. (1997), 'Regression analysis for a functional response', *Technometrics* **39**(3), 254–261.

Goldsmith, J., Zipunnikov, V. & Schrack, J. (2015), 'Generalized multilevel function-on-scalar regression and principal component analysis', *Biometrics* **71**(2), 344–353.

Haegens, S., Cousijn, H., Wallis, G., Harrison, P. J. & Nobre, A. C. (2014), 'Inter-and intra-individual variability in alpha peak frequency', *Neuroimage* **92**, 46–55.

Kowal, D. R. & Bourgeois, D. C. (2020), 'Bayesian function-on-scalars regression for high-dimensional data', *Journal of Computational and Graphical Statistics* **29**(3), 629–638.

Krafty, R. T., Gimotty, P. A., Holtz, D., Coukos, G. & Guo, W. (2008), 'Varying coefficient model with unknown within-subject covariance for analysis of tumor growth curves', *Biometrics* **64**(4), 1023–1031.

Lewis, P. O., Xie, W., Chen, M.-H., Fan, Y. & Kuo, L. (2014), 'Posterior predictive bayesian phylogenetic model selection', *Systematic biology* **63**(3), 309–321.

Marco, N., Şentürk, D., Jeste, S., DiStefano, C., Dickinson, A. & Telesca, D. (2024), 'Functional mixed membership models', *Journal of Computational and Graphical Statistics* pp. 1–11.

Morris, J. S. (2015), 'Functional regression', *Annual Review of Statistics and Its Application* **2**, 321–359.

Morris, J. S. & Carroll, R. J. (2006), 'Wavelet-based functional mixed models', *Journal of the Royal Statistical Society: Series B (Statistical Methodology)* **68**(2), 179–199.

Murphy, K. & Murphy, T. B. (2020), 'Gaussian parsimonious clustering models with covariates and a noise component', *Advances in Data Analysis and Classification* **14**(2), 293–325.

Pettit, L. (1990), ‘The conditional predictive ordinate for the normal distribution’, *Journal of the Royal Statistical Society: Series B (Methodological)* **52**(1), 175–184.

Pritchard, J. K., Stephens, M. & Donnelly, P. (2000), ‘Inference of population structure using multilocus genotype data’, *Genetics* **155**(2), 945–959.

Ramsay, J. & Silverman, B. (2005), *Functional Data Analysis*, Springer Series in Statistics, Springer. **URL:** *https://books.google.com/books?id=mU3dop5wY_4C*

Reiss, P. T., Huang, L. & Mennes, M. (2010), ‘Fast function-on-scalar regression with penalized basis expansions’, *The international journal of biostatistics* **6**(1).

Rice, J. A. & Silverman, B. W. (1991), ‘Estimating the mean and covariance structure nonparametrically when the data are curves’, *Journal of the Royal Statistical Society: Series B (Methodological)* **53**(1), 233–243.

Rodríguez-Martínez, E., Ruiz-Martínez, F., Paulino, C. B. & Gómez, C. M. (2017), ‘Frequency shift in topography of spontaneous brain rhythms from childhood to adulthood’, *Cognitive neurodynamics* **11**(1), 23–33.

Roeder, K. & Wasserman, L. (1997), ‘Practical bayesian density estimation using mixtures of normals’, *Journal of the American Statistical Association* **92**(439), 894–902.

Scheffler, A. W., Telesca, D., Sugar, C. A., Jeste, S., Dickinson, A., DiStefano, C. & Şentürk, D. (2019), ‘Covariate-adjusted region-referenced generalized functional linear model for eeg data’, *Statistics in medicine* **38**(30), 5587–5602.

Schwarz, G. (1978), ‘Estimating the dimension of a model’, *The annals of statistics* pp. 461–464.

Shang, H. L. (2014), ‘A survey of functional principal component analysis’, *AStA Advances in Statistical Analysis* **98**, 121–142.

Spiegelhalter, D. J., Best, N. G., Carlin, B. P. & Van Der Linde, A. (2002), ‘Bayesian measures

of model complexity and fit', *Journal of the royal statistical society: Series B (statistical methodology)* **64**(4), 583–639.

Staicu, A.-M., Crainiceanu, C. M. & Carroll, R. J. (2010), 'Fast methods for spatially correlated multilevel functional data', *Biostatistics* **11**(2), 177–194.

Yao, F., Müller, H.-G. & Wang, J.-L. (2005), 'Functional data analysis for sparse longitudinal data', *Journal of the American statistical association* **100**(470), 577–590.
